# Current-Density Calculations on Zn-Porphyrin_40_ Nanorings

**DOI:** 10.1021/acs.jpca.3c03564

**Published:** 2023-09-04

**Authors:** Atif Mahmood, Maria Dimitrova, Dage Sundholm

**Affiliations:** Department of Chemistry, University of Helsinki, P.O. Box 55, A. I. Virtasen Aukio 1, FIN-00014 Helsinki, Finland

## Abstract

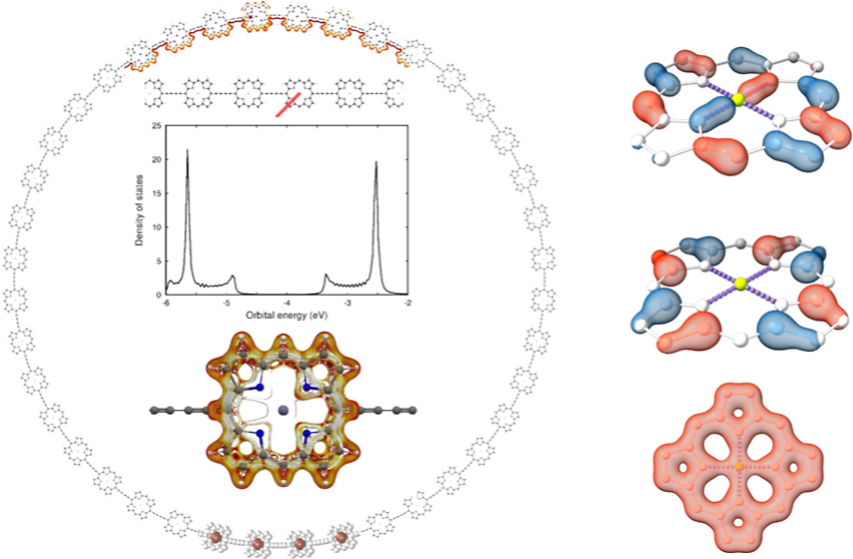

Two porphyrinoid nanorings have been studied computationally. They were built by linking 40 Zn-porphyrin units with butadiyne bridges. The molecular structures belonging to the *D*_40*h*_ point group were fully optimized with the Turbomole program at the density functional theory (DFT) level using the B3LYP functional and the def2-SVP basis sets. The aromatic character was studied at the DFT level by calculating the magnetically induced current-density (MICD) susceptibility using the GIMIC program. The neutral molecules are globally non-aromatic with aromatic Zn-porphyrin units. Charged nanorings could not be studied because almost degenerate frontier orbitals led to vanishing optical gaps for the cations. Since DFT calculations of the MICD are computationally expensive, we also calculated the MICD using three pseudo-π models. Appropriate pseudo-π models were constructed by removing the outer hydrogen atoms and replacing all carbon and nitrogen atoms with hydrogen atoms. The central Zn atom was either replaced with a beryllium atom or with two inner hydrogen atoms. Calculations with the computationally inexpensive pseudo-π models yielded qualitatively the same magnetic response as obtained in the all-electron calculations.

## 1 Introduction

Aromaticity is an old but a very important concept in chemistry.^1^ It was originally introduced for explaining properties of planar molecular rings of organic molecules like benzene.^2−4^ The aromaticity concept has been generalized to annulene rings of different size, to polycyclic aromatic hydrocarbons, and to multiring heterocylces like porphyrins.^5−11^ It has been extended to antiaromatic molecules.^12^ The degree of aromaticity has more lately been investigated to understand electronic properties of more exotic molecular structures.^13−37^

Aromaticity increases the thermodynamic stability of molecules. It leads to smaller bond-length alternation and affects chemical reactivity. There is no consensus on a single definition of aromaticity.^1^ Aromaticity can be identified by measuring ^1^H NMR chemical shifts^38^ because aromatic rings sustain magnetically induced ring currents that flow in the classical direction around the ring when the molecule is exposed to an external magnetic field.^10,39−44^ Molecular rings sustaining magnetically induced ring currents in the non-classical direction are antiaromatic.^12,45^ The magnetic response due to the magnetically induced current density (MICD) is one of the more popular and powerful measures of molecular aromaticity.^38,42,46−48^ The degree of molecular aromaticity according to the ring-current criterion can be estimated by integrating the current density passing through a plane that cuts molecular rings.^10,46,49,50^ The magnetic criterion is currently probably the only means to determine the global aromatic nature of nanosized molecules like porphyrin nanostructures. The degree of aromaticity according to the magnetic criterion can be calculated for nanosized molecules^51−59^ and deduced from spectroscopic measurements.^55−57,60−65^

A Zn-porphyrin (ZnP) nanoring consisting of 40 units (P_40_) has been synthesized and transferred to an Ag(111) surface where it was characterized using scanning tunneling microscopy (STM) and scanning tunneling spectroscopy (STS).^66^ A combination of tight-binding and density function theory (DFT) calculations was used in studies of the spatial distribution of the electronic states of the P_40_ ring.^66^ The synthesis of the P_40_ ring triggered a discussion of how the degree of aromaticity and antiaromaticity depends on the size of the molecular ring and the number of electrons in nanosized rings.^57^ In computational studies of ring currents, the employed computational level must be chosen with care because calculated strengths of the magnetically induced ring current were found to depend on the employed computational level.^67−69^

Computational studies of the MICD susceptibility (MICD or current density) of nanostructures are demanding since the molecules can consist of hundreds or even thousands of atoms. The pseudo-π approximation, which was originally introduced for studies of the MICD of small hydrocarbons,^70^ has successfully been used in current-density studies of large aromatic hydrocarbons and nanosized all-carbon structures.^35,71,72^ Construction of a pseudo-π model for studies of the MICD of porphyrin nanostructures is not obvious because porphyrins contain nitrogen atoms, and a metal cation or eventually inner hydrogen atoms that have to be accounted for in the model because the orbitals of the inner hydrogen atoms or the valence electrons of the metal atom contribute to the conjugated bonding of the porphyrin.

Here, we have computationally studied two P_40_ rings. One has a vertical orientation of the Zn porphyrin rings with respect to the symmetry axis, whereas the horizontal P_40_ ring is planar as the one studied on the Ag surface.^66^ The strength of the ring currents of the two P_40_ molecules has been obtained by integrating the MICD passing through a plane cutting the molecular ring. The magnetic field was oriented along the *C*_40_ symmetry axis, that is perpendicular to the plane of one of the ZnP molecule. The MICD was obtained in an all-electron calculation at the density functional theory (DFT) level. We also have developed computationally cheaper pseudo-π models that were used in calculations of the MICD. The reliability of the pseudo-π models was tested on free-base porphyrin (H_2_P) and on ZnP. The employed computational methods are described in Section 2, where the results of the tested pseudo-π models are also presented. The obtained results for the vertical and horizontal P_40_ molecules are described in Section 3 and the main conclusions are drawn in Section 4.

## 2 Computational Methods

### 2.1 Molecular Structures

The initial molecular structures were constructed by using the symmetry tool in the define module of the TURBOMOLE program.^73,74^ One ZnP molecule and the bridging C≡C–C≡C unit were placed at an appropriate distance from what will become the center of molecule. The rest of the units were copied when assuming that the molecule belongs to the *D*_40*h*_ point group. The molecules were then fully optimized in the *D*_40*h*_ point group at the B3LYP/def2-SVP level of theory.^75 −80^ The Cartesian coordinates of the optimized molecular structures are given as Supporting Information (SI).

### 2.2 Current Density Calculations

Nuclear magnetic resonance (NMR) shielding constants were calculated at the B3LYP/def2-SVP level of theory using the mpshift module of TURBOMOLE.^81^ The same methodology has recently been used in studies of the current density of porphyrin nanostructures.^58,59^ Calculations of the NMR shielding constants yielded the density matrix and the magnetically perturbed density matrices, which together with basis-set information and structural data were used as input for calculations of the MICD susceptibility in the limit of vanishing magnetic field with the gauge-including magnetically induced current (GIMIC) method.^46,49,50,82−84^ The GIMIC program is freely available and interfaced to common quantum chemistry software packages including the employed TURBOMOLE program.^82^

MICD is a tensor function that is contracted to the current-density vector function by an external magnetic field of a given direction. Integration of the MICD passing through a plane that cuts a molecular ring or chemical bonds yields ring-current (susceptibility) strengths (in nA/T). Ring currents in the classical direction are called diatropic and those in the opposite direction are paratropic. The tropicity of the MICD in a given point in space is a global property that can be determined by following the vector field around the whole current-density vortex.^85^ However, for simple ring-shaped molecules, the local direction of the MICD is sufficient to identify its tropicity. Aromatic molecular rings sustain a net diatropic ring current, and for antiaromatic molecules, the net ring current is paratropic.

Current densities can also be investigated visually by using for example the Paraview program.^86^ A set of points in an inspection sphere with a given radius and point density are employed to trace the vector field. The current-density pictures were made with Paraview^86^ and the pictures of the molecular structures with VMD.^87^ The density-of-states graph was plotted with Gnuplot.^88^

### 2.3 Pseudo-π Model

Fowler and Steiner proposed in 2002 the pseudo-π model for computing the MICD of small hydrocarbon rings.^70^ The π orbitals are represented by the 1s orbitals of hydrogen atoms. The pseudo-π orbitals are obtained in a self-consistent-field (SCF) calculation for a system where the carbon atoms are replaced by hydrogen atoms and eventual hydrogen atoms of the original molecule are removed. The hypothetical pseudo-π systems mimic accurately the magnetic response of the molecules exposed to an external magnetic field. The pseudo-π model that has been applied to hydrocarbons and all-carbon structures reduces dramatically the computational costs without significantly affecting the magnetic response.^35,70−72^

**Figure 1:**
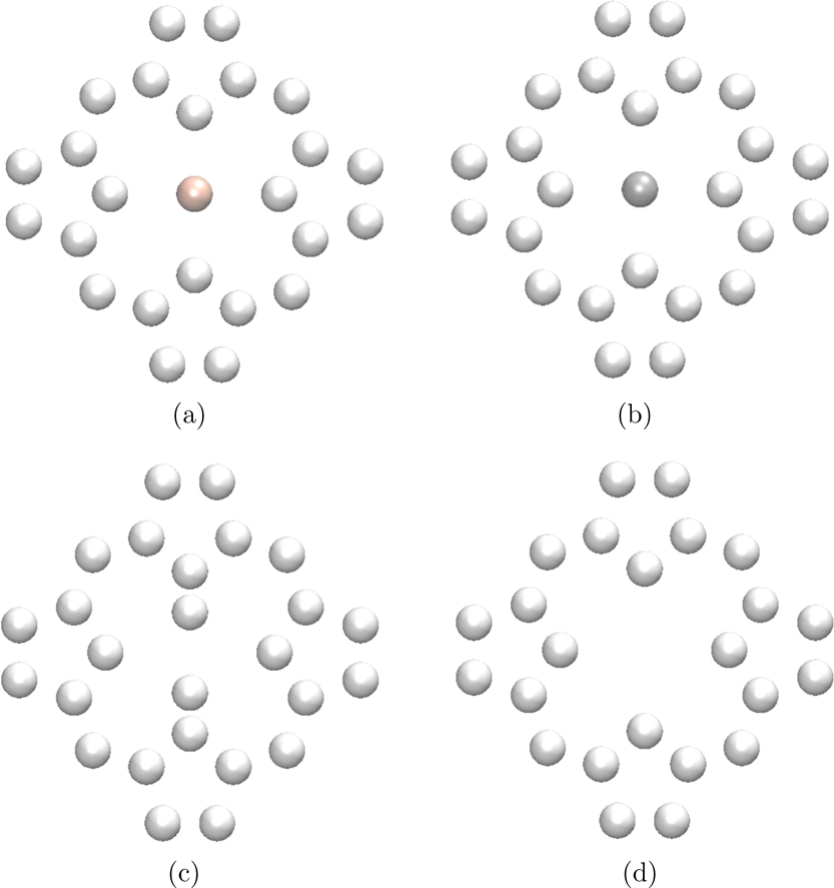
Pseudo-π models **1**, **2**, **3**, and **4** are shown in (a), (b), (c), and (d), respectively. The hydrogen atoms are shown in light gray, beryllium is reddish, and zinc is dark gray. The pictures have been made with VMD.^87^

Since we study ZnP-based porphyrin structures that consist of carbon, nitrogen, and zinc atoms in addition to the outer hydrogen atoms, we have adapted the original pseudo-π model for studies of the magnetic response of H_2_P and ZnP structures. We remove the outer hydrogen atoms. The carbon and nitrogen atoms are replaced with hydrogen atoms. For free-based porphyrins, we keep the inner hydrogen atoms. We also tested what happens when one removes the inner hydrogen atoms. However, the obtained results show that the dehydro pseudo-π model does not work. The Zn atoms in the center of the porphyrins were either kept as such or they were replaced with beryllium atoms, which have fewer electrons but donate two electrons to the surrounding porphyrin ring. The pseudo-π model was tested on H_2_P and ZnP. The pseudo-π models of the porphyrin molecules are shown in Figure 1. The calculations on the pseudo-π models do not suffer from any SCF convergence problems. They do not exhibit any singlet or triplet instabilities. Pictures of the electron density and frontier orbitals of the Be-based pseudo-π porphyrin in the Supporting Information show chemical bonds between the atoms. The obtained ring-current strengths for the porphyrins are summarized in Table 1.

**Table 1:** Ring-Current Strengths (in nA/T) and the Diatropic and Paratropic Contributions to the Global Ring-Current Strengths Obtained with the Pseudo-π Models are Compared to the Corresponding Values Obtained in the All-Electron Calculations on ZnP and H_2_P

molecular system	diatropic	paratropic	net
ZnP	35.11	–5.49	29.62
pseudo-π model **1** (BeP-based)	26.30	0.00	26.30
pseudo-π model **2** (ZnP-based)	24.96	0.00	24.96
free-base porphyrin	35.46	–8.77	26.69
pseudo-π model **3** (H_2_P-based)	22.03	0.00	22.03
pseudo-π model **4** (dehydro)	16.37	–135.3	–118.9

The aromatic character of molecular rings can be estimated from ring-current strengths, which correlate with the degree of aromaticity. Molecules sustaining a strong diatropic ring current are assumed to be aromatic, whereas strong paratropic ring currents are associated with antiaromaticity. A single H_2_P ring is aromatic sustaining a ring current of 26.7 nA/T according to the calculation at the B3LYP/def2-SVP level of theory. Current-density calculations on aromatic porphyrinoids generally yield accurate ring-current strengths, whereas B3LYP calculations overestimate the ring-current strength of strongly antiaromatic porphyrinoids.^89,90^ The ring-current strength of ZnP is 29.6 nA/T at the same level of theory. The ring-current strength of H_2_P and ZnP was also studied using four pseudo-π models. Model **1** is a Be-based pseudo-π model, where the central Zn atom is replaced with a beryllium atom and the carbon and nitrogen atoms are replaced with hydrogen atoms. The outer hydrogen atoms are removed. Model **2** is similar to model **1**. The only difference is that the Zn atom is not replaced with a beryllium atom. In model **3**, we used the modified pseudo-π model on H_2_P without removing the inner hydrogen atoms. Model **4** is a dehydro model of **3**, where the inner hydrogen atoms are removed.

Calculations using models **1** to **3** yielded ring-current strengths in close agreement with those obtained in the all-electron calculations on H_2_P and ZnP, whereas the calculation using model **4** yielded a very strong paratropic ring current implying that it does not simulate the magnetic response of H_2_P correctly. The pseudo-π calculations show that the Be-based model (**1**) can be used for simulating the magnetic response of ZnP nanostructures. The ring-current strength for ZnP calculated with model **1** is 26.3 nA/T, which is only 3.3 nA/T smaller than the one obtained in the all-electron calculation. The ring-current strength obtained with the pseudo-π models **1** and **2** are 11 and 16% weaker than the ones obtained in the all-electron calculations. The pyrrole-like rings of model **3** (pseudo-H_2_P) sustain a local paratropic ring current of 1.19 nA/T as compared to the local paratropic ring current of 5.07 nA/T inside the pyrrole rings of H_2_P. The **1** (pseudo-BeP) and **2** (pseudo-ZnP) models do not sustain any local nor global paratropic ring currents. The profiles of the current density are given in the Supporting Information. Calculations using the pseudo-π models **1** and **2** yielded very similar ring-current strengths implying that the Zn atoms can be replaced with Be. The net ring-current strength for H_2_P obtained with model **3** is 4.6 nA/T (17%) weaker than the one obtained in the all-electron calculation.

## 3 Current-Density Calculations on the P_40_ Rings

The P_40_ rings consist of 40 ZnPs linked with butadiyne (C≡C–C≡C) bridges implying that they have 1560 atoms. DFT calculations on such big molecules are computationally expensive. The molecular structures can be optimized and nuclear magnetic shielding constants can be calculated using basis sets of split-valence polarization quality thanks to the high symmetry; the molecular structure of the P_40_ rings are assumed to belong to the *D*_40*h*_ point group. Calculations and analysis of the MICD is computationally expensive because it has to be evaluated in a large number of grid points. MICD calculations using the pseudo-π models are much faster because the number of basis functions is only five times the number of hydrogen atoms. In the calculations on the P_40_ rings, we employed the pseudo-π model **1** with Zn replaced with Be and model **3** representing the free-base P_40_ rings in addition to the all-electron DFT calculations, which are used as reference calculations. The P_40_ rings can have two orientations. In the vertical P_40_ ring, the ZnP units are parallel to the *C*_40_ symmetry axis, whereas in the horizontal P_40_, they are perpendicular to it.

### 3.1 Vertical P_40_

**Figure 2:**
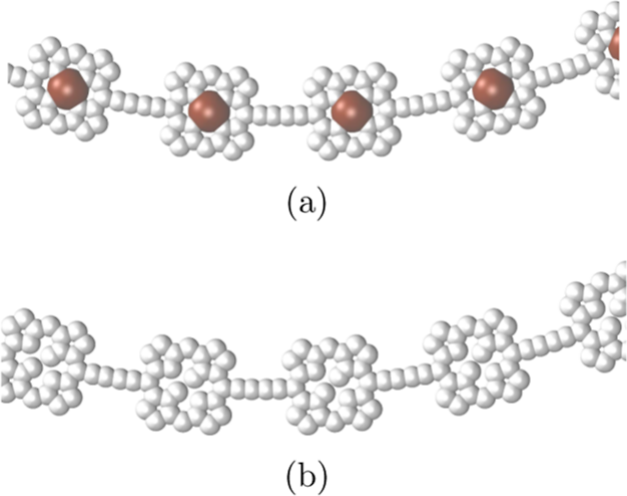
Pseudo-π models **1** and **3** of the vertical P_40_ are shown for a small part of the ring. The hydrogen atoms are light gray and the beryllium atoms are reddish. The pictures have been made with VMD.^87^

Comparison with the MICD calculated at the all-electron level shows that the pseudo-π models shown in Figure 2 work well for the vertical P_40_ ring.

**Figure 3:**
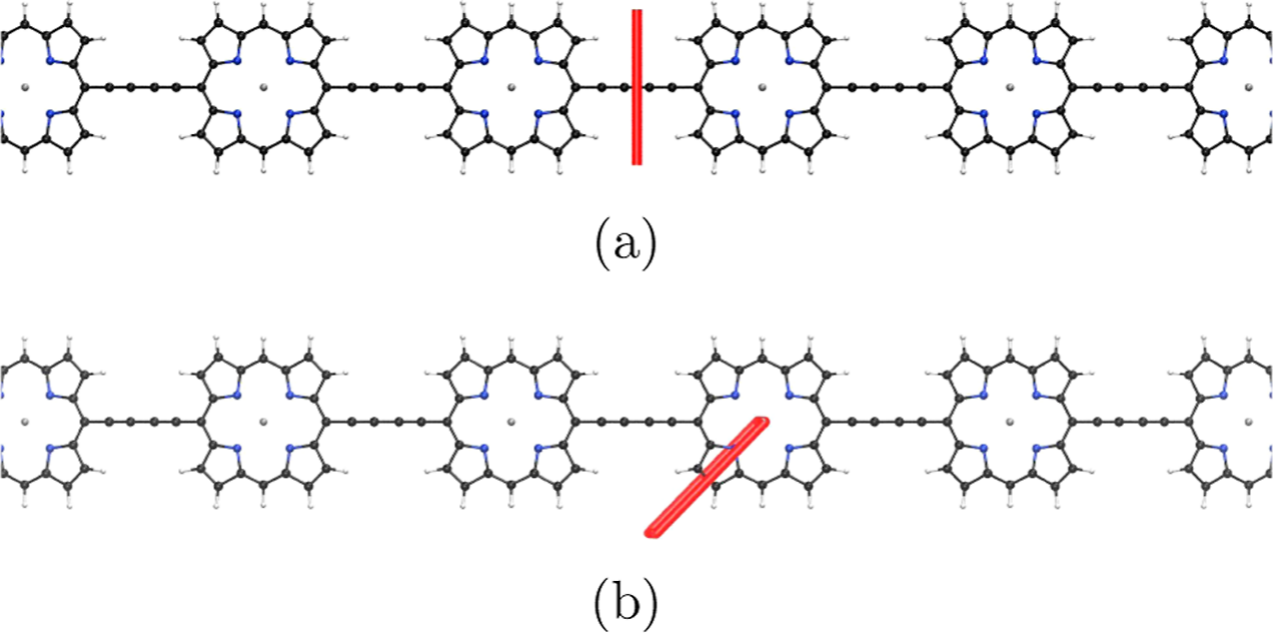
Positions of the integration planes 1A and 1B in the vertical P_40_ are shown in red in (a) and (b), respectively. The integrated current-density strengths passing through the planes are summarized in Table 2. The magnetic field vector is parallel to the integration planes and perpendicular to the picture of the molecule. The pictures have been made with VMD.^87^

Integration of the MICD passing through plane 1A and 1B of the vertical P_40_ at the pseudo-π level yielded net ring-current strengths in close agreement with the one obtained in the all-electron calculation. The integration planes are shown in red in Figure 3. The magnetic field is parallel to the integration plane and perpendicular to the picture of the molecule. The integration planes are generally placed through chemical bonds to avoid the strong MICD vortices near atoms. Plane 1A cuts the P_40_ ring in the middle of the C≡C–C≡C moiety between two porphyrin units. Plane 1B passes through the nitrogen atom of the pyrrole ring. However, with a dense integration grid the same net ring-current strength is obtained regardless of the position of the plane due to the charge-conservation condition. The integrated ring-current strengths show that the P_40_ nanoring is nonaromatic with aromatic ZnP rings. The profile of the current density passing through plane 1B is shown in the Supporting Information.

**Figure 4:**
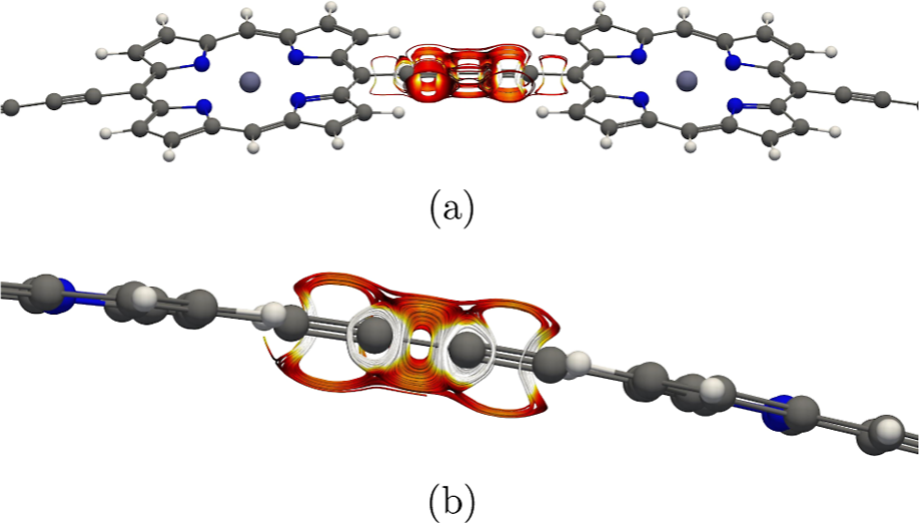
Local current-density vortex in a C≡C–C≡C unit of the vertical P_40_. The pictures have been made with Paraview.^86^

The net ring current around the vertical P_40_ nanoring consists of canceling diatropic and paratropic contributions. However, these contributions originate mainly from the local bond current in the C≡C–C≡C bridge as shown in Figure 4. The strength of the current-density vortex in the C≡C–C≡C moiety is much weaker in the pseudo-π calculations. However, the net ring current strength around the P_40_ nanoring vanishes at the two computational levels. The net strength of the local ring current around the ZnP rings of 26.18 nA/T is qualitatively the same at the all-electron level and at the pseudo-π levels. The Zn atom sustains an atomic current of 34.56 nA/T. The ring current of the ZnP rings is slightly weaker at the pseudo-π levels. The diatropic contribution to the ring current of the ZnP rings is larger at the all-electron level, whereas it is to some extent canceled by the local paratropic contribution in the pyrrole rings. At the pseudo-π levels, the paratropic contribution to the ring current of the ZnP rings of the vertical P_40_ is very weak. The calculated current-density strengths of the vertical P_40_ are summarized in Table 2. The ZnP ring current obtained in P_40_ with the pseudo-π model **1** is 10% weaker than for the all-electron system. The strength of the inner current-density pathway at the plane 1B is much stronger at the pseudo-π model **3** level than at the other levels of theory probably because model **3** has 18 hydrogen atoms along the inner pathway corresponding to Hückel aromaticity. The current-density profiles are shown in the Supporting Information.

**Table 2:** Strength (in nA/T) of the Diatropic and Paratropic Contributions to the Global MICD Passing the Integration Planes of Vertical P_40_^a^

	diatropic	paratropic	net
Plane 1A			
all-electron in the P_40_ ring	7.60	–7.65	–0.05
pseudo-π model **1**	1.49	–1.49	–0.00
pseudo-π model **3**	1.36	–1.66	–0.30
Plane 1B			
all-electron in ZnP	32.01	–5.83	26.18
pseudo-π model **1**	23.55	0.00	23.55
pseudo-π model **3**	20.84	0.00	20.84

a The strengths obtained using the pseudo-π models are compared to the ones calculated at the all-electron level.

### 3.2 Horizontal P_40_

The horizontal P_40_ nanoring has been synthesized and transferred to an Ag(111) surface where the molecule was studied by using a combination of STM and STS, which yielded information about the electronic structure and the spatial position of the electronic states.^66^ The P_40_ nanoring was found to be very flexible having an almost circular structure with a radius of 8.6 nm corresponding to a perimeter of 53.0 nm. It has also a more elongated nearly rectangular structure with side lengths of 13.0 and 18.4 nm as well as intermediate structures between the two.^66^ Here, we study only the circular one whose molecular structure belongs to the *D*_40*h*_ point group because the calculations are much faster for molecules with high symmetry.

Current-density calculations show that horizontal P_40_ does not sustain any net ring current around the nanoring when exposing it to a weak magnetic field. The calculated MICD is obtained in the limit of a vanishing magnetic field and it is approximately linearly dependent on the strength of the external magnetic field, at least as long as the magnetic field is weak. The magnetic response of large molecular rings might depend nonlinearly on the strength of the external magnetic field.^57^

**Figure 5:**
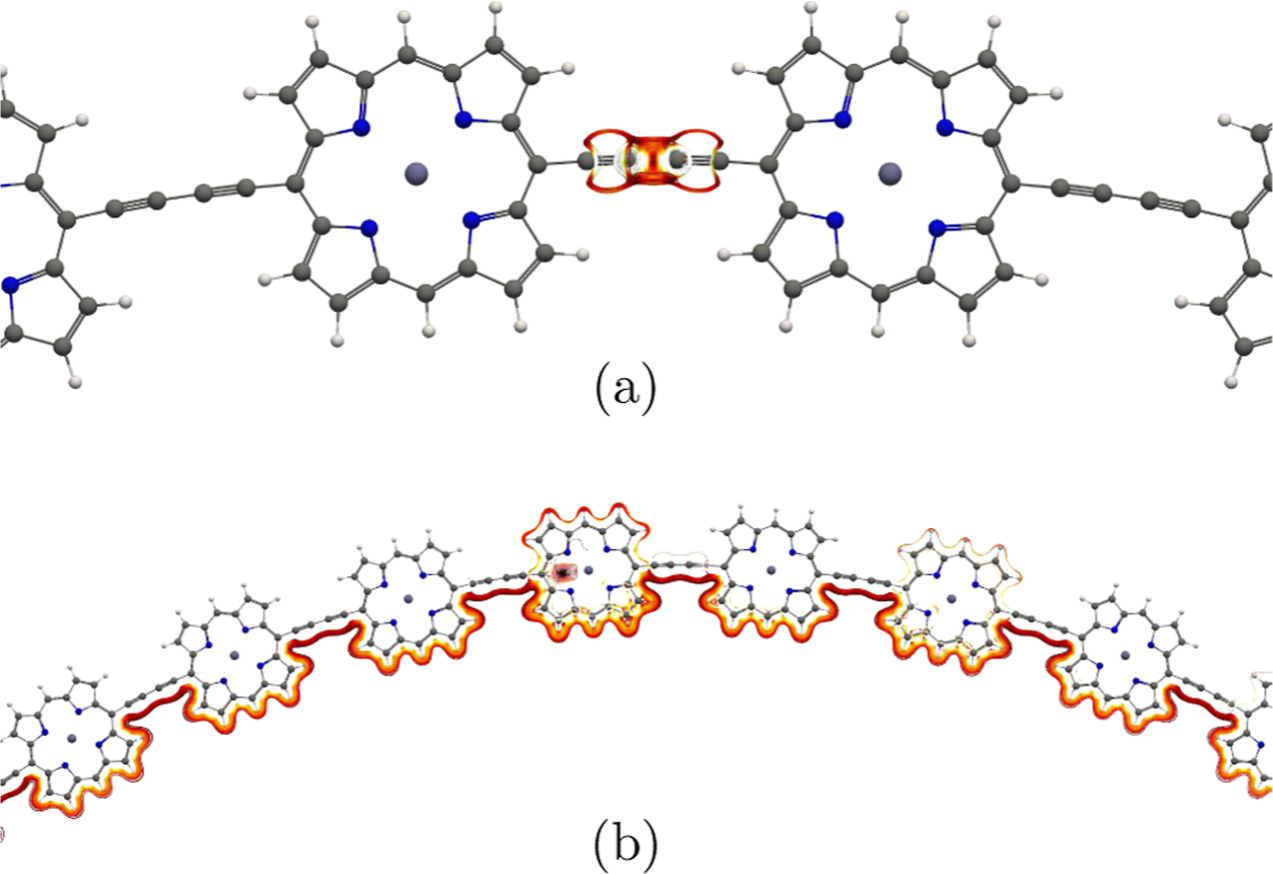
(a) The local current-density vortex in the C≡C–C≡C bridge of the horizontal P_40_ ring. (b) The global paratropic current-density pathway inside the horizontal P_40_ ring. The pictures have been made with Paraview.^86^

The external magnetic field induces a paratropic global ring current on the inside of the horizontal P_40_ ring and an equally strong diatropic ring current on the outside of the ring. Thus, the net ring current vanishes when the magnetic field is applied along the symmetry axis. The MICD at the C≡C–C≡C units has also a local current-density vortex. The global and local current-density pathways are shown in Figure 5.

**Figure 6:**
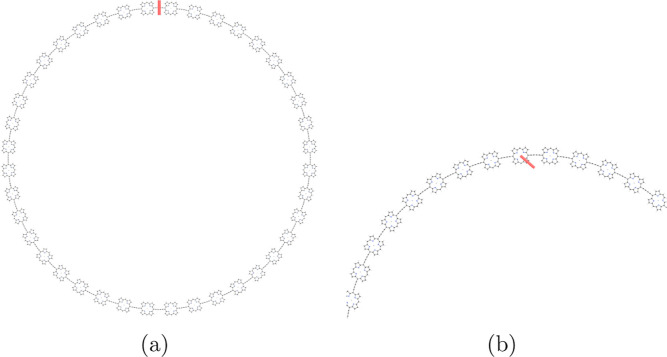
Positions of the integration planes 2A and 2B in horizontal P_40_ are shown in red in (a) and (b), respectively. The integrated current-density strengths are summarized in Table 3. The magnetic field vector is parallel to the integration planes and perpendicular to the picture of the molecule. The pictures have been made with VMD.^87^

Integration of the MICD passing through plane 2A and 2B of the horizontal P_40_ nanoring at the pseudo-π level yielded net ring-current strengths that agree well with the ones obtained in the all-electron calculation. The integration planes for horizontal P_40_ are shown in red in Figure 6. The profile of the current density passing through plane 2B is shown in the Supporting Information. The obtained current-density strengths are summarized in Table 3. The calculations using the pseudo-π models yield smaller paratropic contributions because the pseudo-π models cannot describe local current-density vortices well. The ring current of the porphyrin rings also prefers the inner pathway at the pyrrole rings in the pseudo-π calculations using model **3**. The magnetic field is applied along the *C*_40_ symmetry axis, which is perpendicular to the picture of the molecule and parallel to the integration planes in Figure 6. The calculations show that the horizontal P_40_ ring is nonaromatic with aromatic ZnP rings as for the vertical P_40_ ring. However, the horizontal P_40_ ring sustains canceling global diatropic and paratropic ring currents, which are not present in the vertical P_40_ ring. The calculated ring current around the ZnP rings of horizontal P_40_ is slightly stronger at the all-electron level than when using the pseudo-π models.

**Table 3:** Strength (in nA/T) of the Diatropic and Paratropic Contributions to the Global MICD Passing the Integration Planes of the Horizontal P_40_ Nanoring are Reported^a^

	diatropic	paratropic	net
Plane 2A			
all-electron in the P_40_ ring	7.60	–7.34	0.26
pseudo-π model **1**	1.70	–1.32	0.38
pseudo-π model **3**	1.66	–1.35	0.31
Plane 2B			
all-electron in ZnP	33.30	–5.63	27.67
pseudo-π model **1**	22.62	0.00	22.62
pseudo-π model **3**	19.12	0.00	19.12

a The strengths obtained using the pseudo-π models are compared to the ones calculated at the all-electron level.

**Figure 7:**
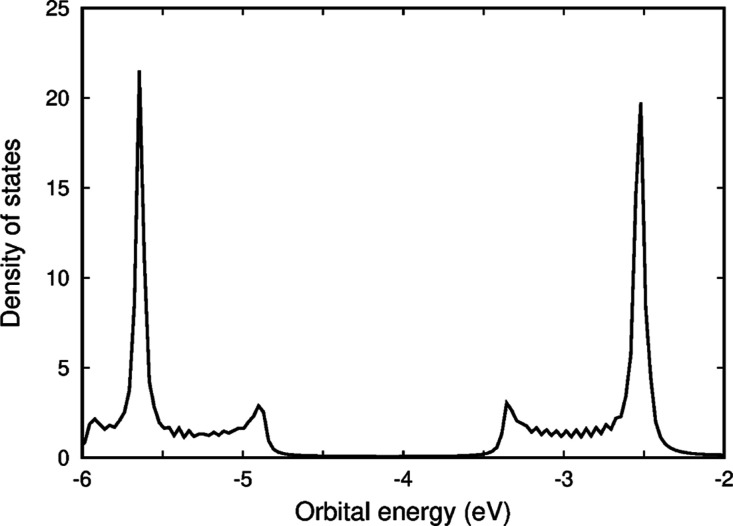
Density of states for the horizontal P_40_. The orbital energy of the HOMO is −4.88 eV and the orbital energy of the LUMO is −3.36 eV. The HOMO–LUMO gaps of the studied molecules are given in the Supporting Information. The picture has been made with Gnuplot.^88^

Previous studies have shown that neutral nonaromatic porphyrinoid structures can be made aromatic or antiaromatic by oxidation.^52,57−59^ The aromatic character might also change when reducing the ring, since the neutral molecule can be considered to be a semiconductor. Oxidation leads to holes in the valence band making the ring conducting or it can sustain a ring current in the present of an external magnetic field. Reduction of the ring leads to electrons in the conduction band, which might also lead to conductivity and a magnetically induced ring current. Electronic structure calculations on large molecular systems with holes in orbitals corresponding to the valence band in the range of [−6.0, −5.0] eV or electrons in orbitals corresponding to the conduction band in the range of [−3.5, −2.5] eV in Figure 7 are difficult due to the small energy difference between the orbitals in the bands. The gap between the highest-occupied molecular orbital (HOMO) and the lowest-unoccupied molecular orbital (LUMO) is very small for charged P_40_ rings and for other porphyrin-based nanostructures.

## 4 Conclusions

The vertical and horizontal P_40_ molecules consisting of 40 ZnPs are local aromatic molecules that do not sustain any significant net global ring current around the P_40_ ring. Calculations at the computationally cheaper pseudo-π levels yielded qualitatively the same magnetic response as obtained at the all-electron level. The porphyrin rings are as aromatic as a single ZnP molecule. The pseudo-π models slightly underestimate the aromatic character of the ZnP rings. Oxidizing the P_40_ rings leads to holes in the orbitals corresponding to the valence band and a small energy gap between the frontier orbitals. Reduction leads to electrons in the conduction band and a small HOMO–LUMO gap. The oxidized or reduced P_40_ rings might sustain ring currents in the presence of an external magnetic field. However, due to the small HOMO–LUMO gap, electronic structure calculations at the DFT level are difficult. Pseudo-π calculations on charged molecules are not reliable, whereas pseudo-π calculations of the magnetic response of neutral porphyrin nanostructures are a very cost-efficient approach.

## Supporting Information Available

Cartesian coordinates of the optimized molecular structures; and ring-current profiles of the studied molecules (PDF)
