# Supplementary material for: Current-Density Calculations on Zn-Porphyrin40 Nanorings
Source: J Phys Chem A. 2023 Sep 4;127(36):7452–9. doi: 10.1021/acs.jpca.3c03564 (PMC10510378; doi:10.1021/acs.jpca.3c03564)
Supplement: Supplementary file 1 — jp3c03564_si_001.pdf [file jp3c03564_si_001.pdf]

Current-Density Calculations on Zn-Porphyrin$_{40}$ Nanorings

Atif Mahmood[a], Maria Dimitrova[a], and Dage Sundholm[a*]

[a] *Department of Chemistry, University of Helsinki, P.O. Box 55, A. I. Virtasen aukio 1, FIN-00014 University of Helsinki, Finland*

E-mail: dage.sundholm@helsinki.fi

Ring-current profiles

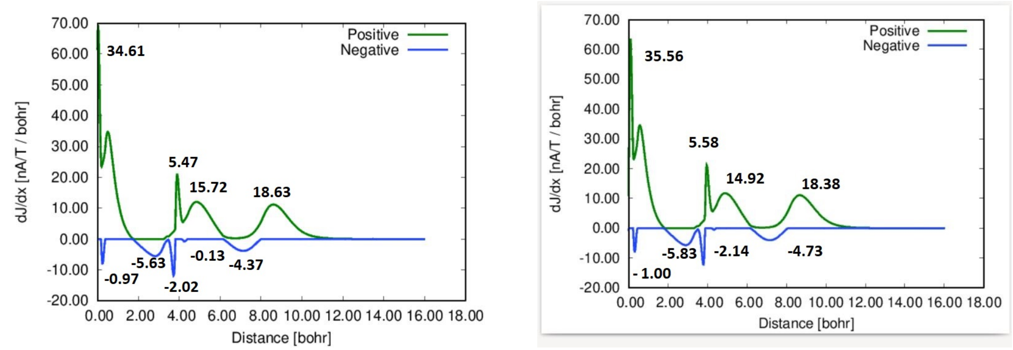

Figure S1: The current-density profile of the horizontal P40 (left) and the vertical P40 (right) obtained in the all-electron calculations. The strong peak is at Zn. The plane passes through the nitrogen atom of the pyrrole ring.

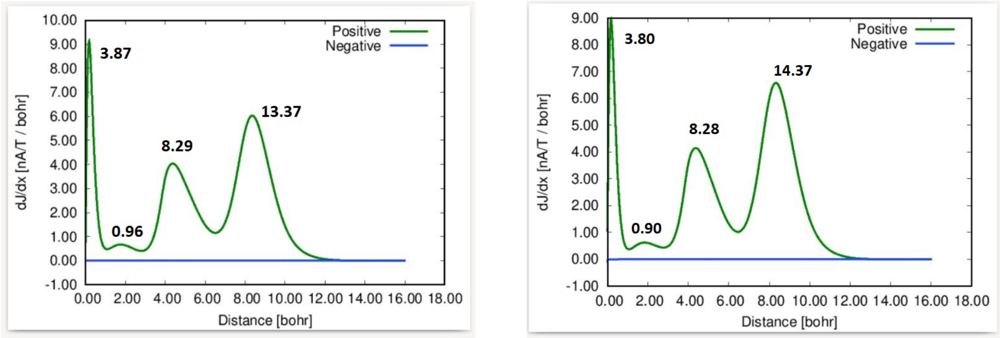

Figure S2: The current-density profile of the horizontal P40 (left) and the vertical P40 (right) obtained in the Be-based pseudo-π calculations. The plane begins at beryllium in the center and passes through the middle of the pyrrole-like ring.

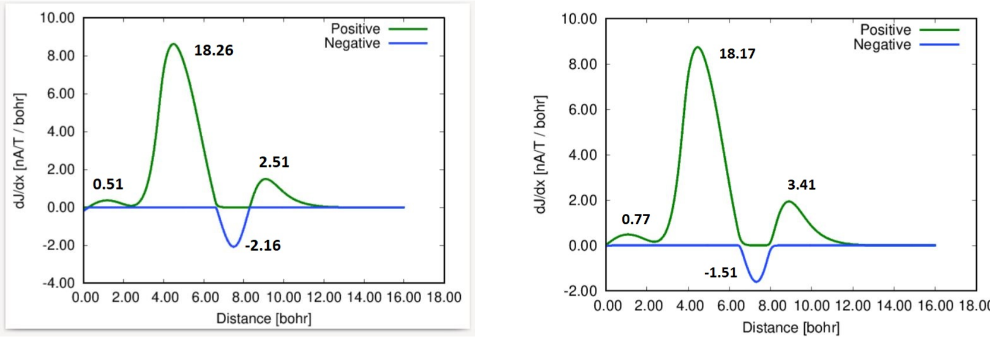

Figure S3: The current-density profile of the horizontal P40 (left) and the vertical P40 (right) obtained in the free-base pseudo-π calculations. The plane passes through the center of the pyrrole-like ring. The smallest peak is inside the porphyrin-like ring.

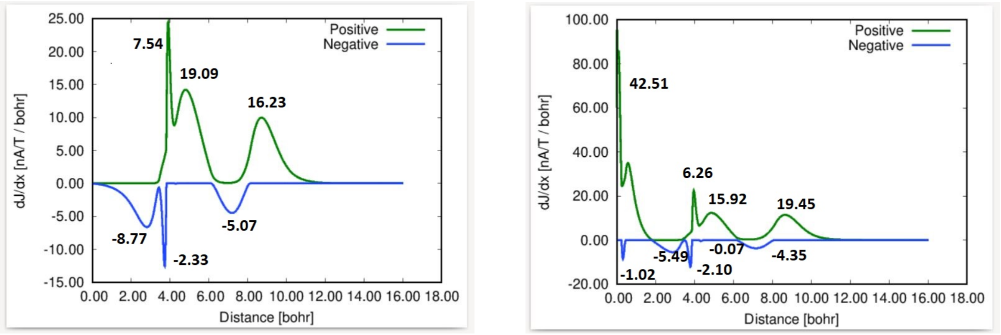

Figure S4: The current-density profile of free-base porphyrin (left) and Zn porphyrin (right) obtained in all-electron calculations. The plane begins in the middle of the porphyrin.

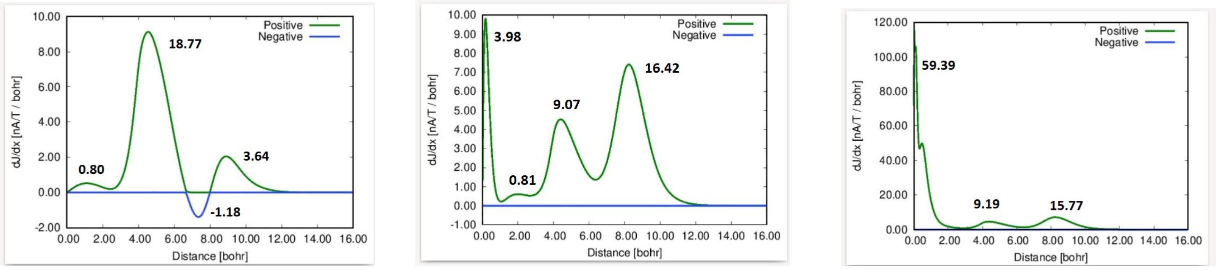

Figure S5: The current-density profile of free-base porphyrin (left), Be porphyrin (middle) and Zn porphyrin (right) obtained in pseudo-π calculations. The plane begins in the middle of the porphyrin-like ring.

Table S1: The energy gap (in eV) between the highest occupied molecular orbital (HOMO) and the lowest unoccupied molecular orbital (LUMO) of the studied molecules.

Molecule	HOMO-LUMO gap
P_{40} Horizontal	1.517
Pseudo-Pi-model 1 (BeP)	1.965
Pseudo-Pi-model 3 (H_2P)	1.871
P_{40} Vertical	1.519
Pseudo-π-model 1 (BeP)	1.967
Pseudo-π-model 3 (H_2P)	1.877
Zn-porphyrin	3.038
Pseudo-π-model 1 (BeP)	3.013
Pseudo-π-model 2 (ZnP)	3.149
Free-base-porphyrin	2.902
Pseudo-π-model 3 (H_2P)	2.503
Pseudo-π-model 4 (dehydro)	1.171

The electron density and frontier orbitals of pseudo-BeP

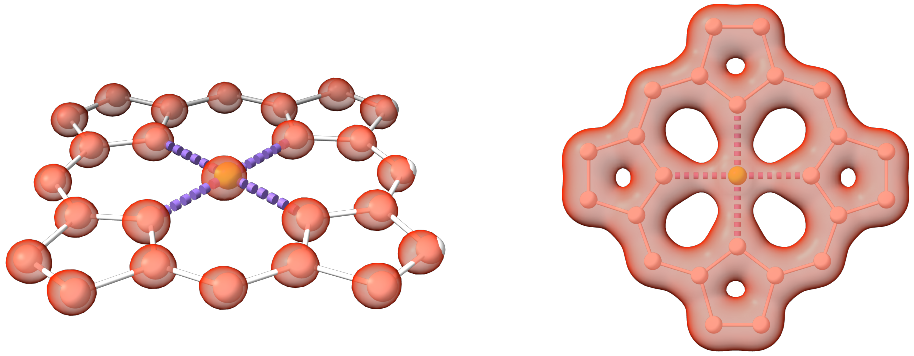

Figure S6: The electron density of pseudo-BeP with two isovalues. The isovalue in the right picture is $2 \cdot 10^{-2}$.

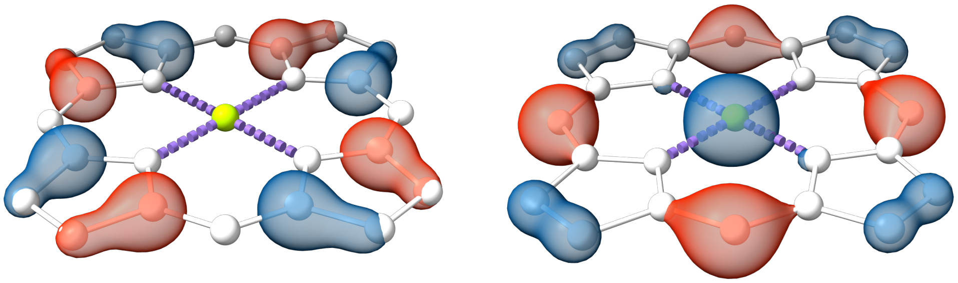

Figure S7: The HOMO (left) and HOMO-1 (right) orbitals of pseudo-BeP.

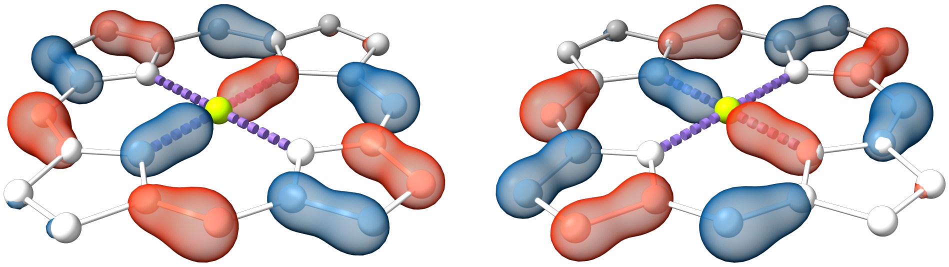

Figure S8: The LUMO (left) and LUMO+1 (right) orbitals of pseudo-BeP.

Optimized coordinates of the molecular structures

Vertical P_{40}

```
1560
Energy =
Zn   86.4691039   -6.8052661    0.0000000
C    86.1467862  -10.2783462   -0.0000000
C    86.2223526   -9.6210280    1.2577416
C    86.1723441  -10.2895167    2.5406830
C    86.2781935   -9.3164632    3.4960576
C    86.3878114   -8.0549440    2.7983138
N    86.3534691   -8.2734953    1.4413028
C    86.4988001   -6.8076032    3.4162659
H    86.5106341   -6.8085346    4.5085013
C    86.5843049   -5.5582567    2.7983138
C    86.6733817   -4.2951210    3.4960576
C    86.7210546   -3.3174889    2.5406830
C    86.6658727   -3.9855704    1.2577416
N    86.5845744   -5.3370239    1.4413028
H    86.0713287  -11.3634815    2.6782730
H    86.2801078   -9.4347363    4.5787072
H    86.6937744   -4.1786035    4.5787072
H    86.7892879   -2.2409441    2.6782730
C    86.6940639   -3.3245237   -0.0000000
C    86.7432775   -1.9085201    0.0000000
C    86.7623482   -0.6796062    0.0000000
C    86.7623482    0.6796062    0.0000000
C    86.7432775    1.9085201   -0.0000000
Zn   86.4691039    6.8052661    0.0000000
Zn   84.3399475   20.2482299    0.0000000
Zn   80.1340618   33.1926152    0.0000000
Zn   73.9550095   45.3196881    0.0000000
Zn   65.9549395   56.3308399    0.0000000
Zn   56.3308399   65.9549395   -0.0000000
Zn   45.3196881   73.9550095    0.0000000
Zn   33.1926152   80.1340618   -0.0000000
Zn   20.2482299   84.3399475   -0.0000000
Zn    6.8052661   86.4691039    0.0000000
Zn   -6.8052661   86.4691039   -0.0000000
Zn  -20.2482299   84.3399475    0.0000000
Zn  -33.1926152   80.1340618   -0.0000000
Zn  -45.3196881   73.9550095    0.0000000
Zn  -56.3308399   65.9549395   -0.0000000
Zn  -65.9549395   56.3308399   -0.0000000
Zn  -73.9550095   45.3196881    0.0000000
Zn  -80.1340618   33.1926152    0.0000000
Zn  -84.3399475   20.2482299    0.0000000
Zn  -86.4691039    6.8052661   -0.0000000
```

Zn	-86.4691039	-6.8052661	0.0000000
Zn	-84.3399475	-20.2482299	-0.0000000
Zn	-80.1340618	-33.1926152	0.0000000
Zn	-73.9550095	-45.3196881	-0.0000000
Zn	-65.9549395	-56.3308399	0.0000000
Zn	-56.3308399	-65.9549395	-0.0000000
Zn	-45.3196881	-73.9550095	-0.0000000
Zn	-33.1926152	-80.1340618	-0.0000000
Zn	-20.2482299	-84.3399475	0.0000000
Zn	-6.8052661	-86.4691039	0.0000000
Zn	6.8052661	-86.4691039	0.0000000
Zn	20.2482299	-84.3399475	-0.0000000
Zn	33.1926152	-80.1340618	-0.0000000
Zn	45.3196881	-73.9550095	0.0000000
Zn	56.3308399	-65.9549395	-0.0000000
Zn	65.9549395	-56.3308399	0.0000000
Zn	73.9550095	-45.3196881	0.0000000
Zn	80.1340618	-33.1926152	0.0000000
Zn	84.3399475	-20.2482299	0.0000000
C	86.6940639	3.3245237	-0.0000000
C	85.1066460	16.8455328	-0.0000000
C	81.4236200	29.9517490	-0.0000000
C	75.7356743	42.3204537	-0.0000000
C	68.1828650	53.6470884	-0.0000000
C	58.9511672	63.6527537	-0.0000000
C	48.2678960	72.0910770	-0.0000000
C	36.3961091	78.7542787	0.0000000
C	23.6281291	83.4782887	-0.0000000
C	10.2783462	86.1467862	-0.0000000
C	-3.3245237	86.6940639	0.0000000
C	-16.8455328	85.1066460	0.0000000
C	-29.9517490	81.4236200	0.0000000
C	-42.3204537	75.7356743	0.0000000
C	-53.6470884	68.1828650	-0.0000000
C	-63.6527537	58.9511672	0.0000000
C	-72.0910770	48.2678960	0.0000000
C	-78.7542787	36.3961091	-0.0000000
C	-83.4782887	23.6281291	-0.0000000
C	-86.1467862	10.2783462	-0.0000000
C	-86.6940639	-3.3245237	-0.0000000
C	-85.1066460	-16.8455328	0.0000000
C	-81.4236200	-29.9517490	0.0000000
C	-75.7356743	-42.3204537	0.0000000
C	-68.1828650	-53.6470884	-0.0000000
C	-58.9511672	-63.6527537	0.0000000
C	-48.2678960	-72.0910770	-0.0000000
C	-36.3961091	-78.7542787	0.0000000
C	-23.6281291	-83.4782887	-0.0000000
C	-10.2783462	-86.1467862	-0.0000000

C	3.3245237	-86.6940639	-0.0000000
C	16.8455328	-85.1066460	0.0000000
C	29.9517490	-81.4236200	-0.0000000
C	42.3204537	-75.7356743	-0.0000000
C	53.6470884	-68.1828650	0.0000000
C	63.6527537	-58.9511672	0.0000000
C	72.0910770	-48.2678960	0.0000000
C	78.7542787	-36.3961091	0.0000000
C	83.4782887	-23.6281291	0.0000000
C	86.1467862	10.2783462	0.0000000
C	85.1066460	-16.8455328	0.0000000
C	81.4236200	-29.9517490	0.0000000
C	75.7356743	-42.3204537	0.0000000
C	68.1828650	-53.6470884	0.0000000
C	58.9511672	-63.6527537	0.0000000
C	48.2678960	-72.0910770	0.0000000
C	36.3961091	-78.7542787	0.0000000
C	23.6281291	-83.4782887	0.0000000
C	10.2783462	-86.1467862	-0.0000000
C	-3.3245237	-86.6940639	0.0000000
C	-16.8455328	-85.1066460	0.0000000
C	-29.9517490	-81.4236200	-0.0000000
C	-42.3204537	-75.7356743	-0.0000000
C	-53.6470884	-68.1828650	0.0000000
C	-63.6527537	-58.9511672	-0.0000000
C	-72.0910770	-48.2678960	0.0000000
C	-78.7542787	-36.3961091	-0.0000000
C	-83.4782887	-23.6281291	-0.0000000
C	-86.1467862	-10.2783462	0.0000000
C	-86.6940639	3.3245237	0.0000000
C	-85.1066460	16.8455328	0.0000000
C	-81.4236200	29.9517490	-0.0000000
C	-75.7356743	42.3204537	-0.0000000
C	-68.1828650	53.6470884	-0.0000000
C	-58.9511672	63.6527537	-0.0000000
C	-48.2678960	72.0910770	-0.0000000
C	-36.3961091	78.7542787	-0.0000000
C	-23.6281291	83.4782887	0.0000000
C	-10.2783462	86.1467862	0.0000000
C	3.3245237	86.6940639	0.0000000
C	16.8455328	85.1066460	0.0000000
C	29.9517490	81.4236200	0.0000000
C	42.3204537	75.7356743	-0.0000000
C	53.6470884	68.1828650	-0.0000000
C	63.6527537	58.9511672	-0.0000000
C	72.0910770	48.2678960	-0.0000000
C	78.7542787	36.3961091	-0.0000000
C	83.4782887	23.6281291	-0.0000000
C	86.6658727	3.9855704	1.2577416

C	84.9753914	17.4940309	1.2577416
C	81.1925340	30.5717302	1.2577416
C	75.4104469	42.8966521	1.2577416
C	67.7715044	54.1653161	1.2577416
C	58.4638024	64.1002502	1.2577416
C	47.7165277	72.4568234	1.2577416
C	35.7943136	79.0292691	1.2577416
C	22.9907248	83.6557520	1.2577416
C	9.6210280	86.2223526	1.2577416
C	-3.9855704	86.6658727	1.2577416
C	-17.4940309	84.9753914	1.2577416
C	-30.5717302	81.1925340	1.2577416
C	-42.8966521	75.4104469	1.2577416
C	-54.1653161	67.7715044	1.2577416
C	-64.1002502	58.4638024	1.2577416
C	-72.4568234	47.7165277	1.2577416
C	-79.0292691	35.7943136	1.2577416
C	-83.6557520	22.9907248	1.2577416
C	-86.2223526	9.6210280	1.2577416
C	-86.6658727	-3.9855704	1.2577416
C	-84.9753914	-17.4940309	1.2577416
C	-81.1925340	-30.5717302	1.2577416
C	-75.4104469	-42.8966521	1.2577416
C	-67.7715044	-54.1653161	1.2577416
C	-58.4638024	-64.1002502	1.2577416
C	-47.7165277	-72.4568234	1.2577416
C	-35.7943136	-79.0292691	1.2577416
C	-22.9907248	-83.6557520	1.2577416
C	-9.6210280	-86.2223526	1.2577416
C	3.9855704	-86.6658727	1.2577416
C	17.4940309	-84.9753914	1.2577416
C	30.5717302	-81.1925340	1.2577416
C	42.8966521	-75.4104469	1.2577416
C	54.1653161	-67.7715044	1.2577416
C	64.1002502	-58.4638024	1.2577416
C	72.4568234	-47.7165277	1.2577416
C	79.0292691	-35.7943136	1.2577416
C	83.6557520	-22.9907248	1.2577416
C	86.2223526	9.6210280	-1.2577416
C	86.6658727	-3.9855704	-1.2577416
C	84.9753914	-17.4940309	-1.2577416
C	81.1925340	-30.5717302	-1.2577416
C	75.4104469	-42.8966521	-1.2577416
C	67.7715044	-54.1653161	-1.2577416
C	58.4638024	-64.1002502	-1.2577416
C	47.7165277	-72.4568234	-1.2577416
C	35.7943136	-79.0292691	-1.2577416
C	22.9907248	-83.6557520	-1.2577416
C	9.6210280	-86.2223526	-1.2577416

C	-3.9855704	-86.6658727	-1.2577416
C	-17.4940309	-84.9753914	-1.2577416
C	-30.5717302	-81.1925340	-1.2577416
C	-42.8966521	-75.4104469	-1.2577416
C	-54.1653161	-67.7715044	-1.2577416
C	-64.1002502	-58.4638024	-1.2577416
C	-72.4568234	-47.7165277	-1.2577416
C	-79.0292691	-35.7943136	-1.2577416
C	-83.6557520	-22.9907248	-1.2577416
C	-86.2223526	-9.6210280	-1.2577416
C	-86.6658727	3.9855704	-1.2577416
C	-84.9753914	17.4940309	-1.2577416
C	-81.1925340	30.5717302	-1.2577416
C	-75.4104469	42.8966521	-1.2577416
C	-67.7715044	54.1653161	-1.2577416
C	-58.4638024	64.1002502	-1.2577416
C	-47.7165277	72.4568234	-1.2577416
C	-35.7943136	79.0292691	-1.2577416
C	-22.9907248	83.6557520	-1.2577416
C	-9.6210280	86.2223526	-1.2577416
C	3.9855704	86.6658727	-1.2577416
C	17.4940309	84.9753914	-1.2577416
C	30.5717302	81.1925340	-1.2577416
C	42.8966521	75.4104469	-1.2577416
C	54.1653161	67.7715044	-1.2577416
C	64.1002502	58.4638024	-1.2577416
C	72.4568234	47.7165277	-1.2577416
C	79.0292691	35.7943136	-1.2577416
C	83.6557520	22.9907248	-1.2577416
C	86.2223526	-9.6210280	-1.2577416
C	86.6658727	3.9855704	-1.2577416
C	84.9753914	17.4940309	-1.2577416
C	81.1925340	30.5717302	-1.2577416
C	75.4104469	42.8966521	-1.2577416
C	67.7715044	54.1653161	-1.2577416
C	58.4638024	64.1002502	-1.2577416
C	47.7165277	72.4568234	-1.2577416
C	35.7943136	79.0292691	-1.2577416
C	22.9907248	83.6557520	-1.2577416
C	9.6210280	86.2223526	-1.2577416
C	-3.9855704	86.6658727	-1.2577416
C	-17.4940309	84.9753914	-1.2577416
C	-30.5717302	81.1925340	-1.2577416
C	-42.8966521	75.4104469	-1.2577416
C	-54.1653161	67.7715044	-1.2577416
C	-64.1002502	58.4638024	-1.2577416
C	-72.4568234	47.7165277	-1.2577416
C	-79.0292691	35.7943136	-1.2577416
C	-83.6557520	22.9907248	-1.2577416

C	-86.2223526	9.6210280	-1.2577416
C	-86.6658727	-3.9855704	-1.2577416
C	-84.9753914	-17.4940309	-1.2577416
C	-81.1925340	-30.5717302	-1.2577416
C	-75.4104469	-42.8966521	-1.2577416
C	-67.7715044	-54.1653161	-1.2577416
C	-58.4638024	-64.1002502	-1.2577416
C	-47.7165277	-72.4568234	-1.2577416
C	-35.7943136	-79.0292691	-1.2577416
C	-22.9907248	-83.6557520	-1.2577416
C	-9.6210280	-86.2223526	-1.2577416
C	3.9855704	-86.6658727	-1.2577416
C	17.4940309	-84.9753914	-1.2577416
C	30.5717302	-81.1925340	-1.2577416
C	42.8966521	-75.4104469	-1.2577416
C	54.1653161	-67.7715044	-1.2577416
C	64.1002502	-58.4638024	-1.2577416
C	72.4568234	-47.7165277	-1.2577416
C	79.0292691	-35.7943136	-1.2577416
C	83.6557520	-22.9907248	-1.2577416
C	86.2223526	9.6210280	1.2577416
C	84.9753914	-17.4940309	1.2577416
C	81.1925340	-30.5717302	1.2577416
C	75.4104469	-42.8966521	1.2577416
C	67.7715044	-54.1653161	1.2577416
C	58.4638024	-64.1002502	1.2577416
C	47.7165277	-72.4568234	1.2577416
C	35.7943136	-79.0292691	1.2577416
C	22.9907248	-83.6557520	1.2577416
C	9.6210280	-86.2223526	1.2577416
C	-3.9855704	-86.6658727	1.2577416
C	-17.4940309	-84.9753914	1.2577416
C	-30.5717302	-81.1925340	1.2577416
C	-42.8966521	-75.4104469	1.2577416
C	-54.1653161	-67.7715044	1.2577416
C	-64.1002502	-58.4638024	1.2577416
C	-72.4568234	-47.7165277	1.2577416
C	-79.0292691	-35.7943136	1.2577416
C	-83.6557520	-22.9907248	1.2577416
C	-86.2223526	-9.6210280	1.2577416
C	-86.6658727	3.9855704	1.2577416
C	-84.9753914	17.4940309	1.2577416
C	-81.1925340	30.5717302	1.2577416
C	-75.4104469	42.8966521	1.2577416
C	-67.7715044	54.1653161	1.2577416
C	-58.4638024	64.1002502	1.2577416
C	-47.7165277	72.4568234	1.2577416
C	-35.7943136	79.0292691	1.2577416
C	-22.9907248	83.6557520	1.2577416

C	-9.6210280	86.2223526	1.2577416
C	3.9855704	86.6658727	1.2577416
C	17.4940309	84.9753914	1.2577416
C	30.5717302	81.1925340	1.2577416
C	42.8966521	75.4104469	1.2577416
C	54.1653161	67.7715044	1.2577416
C	64.1002502	58.4638024	1.2577416
C	72.4568234	47.7165277	1.2577416
C	79.0292691	35.7943136	1.2577416
C	83.6557520	22.9907248	1.2577416
C	86.7210546	3.3174889	2.5406830
C	85.1344049	16.8428068	2.5406830
C	81.4514636	29.9533990	2.5406830
C	75.7629170	42.3264391	2.5406830
C	68.2088359	53.6572618	2.5406830
C	58.9752269	63.6668646	2.5406830
C	48.2894521	72.1087780	2.5406830
C	36.4146307	78.7751338	2.5406830
C	23.6431603	83.5017845	2.5406830
C	10.2895167	86.1723441	2.5406830
C	-3.3174889	86.7210546	2.5406830
C	-16.8428068	85.1344049	2.5406830
C	-29.9533990	81.4514636	2.5406830
C	-42.3264391	75.7629170	2.5406830
C	-53.6572618	68.2088359	2.5406830
C	-63.6668646	58.9752269	2.5406830
C	-72.1087780	48.2894521	2.5406830
C	-78.7751338	36.4146307	2.5406830
C	-83.5017845	23.6431603	2.5406830
C	-86.1723441	10.2895167	2.5406830
C	-86.7210546	-3.3174889	2.5406830
C	-85.1344049	-16.8428068	2.5406830
C	-81.4514636	-29.9533990	2.5406830
C	-75.7629170	-42.3264391	2.5406830
C	-68.2088359	-53.6572618	2.5406830
C	-58.9752269	-63.6668646	2.5406830
C	-48.2894521	-72.1087780	2.5406830
C	-36.4146307	-78.7751338	2.5406830
C	-23.6431603	-83.5017845	2.5406830
C	-10.2895167	-86.1723441	2.5406830
C	3.3174889	-86.7210546	2.5406830
C	16.8428068	-85.1344049	2.5406830
C	29.9533990	-81.4514636	2.5406830
C	42.3264391	-75.7629170	2.5406830
C	53.6572618	-68.2088359	2.5406830
C	63.6668646	-58.9752269	2.5406830
C	72.1087780	-48.2894521	2.5406830
C	78.7751338	-36.4146307	2.5406830
C	83.5017845	-23.6431603	2.5406830

C	86.1723441	10.2895167	-2.5406830
C	86.7210546	-3.3174889	-2.5406830
C	85.1344049	-16.8428068	-2.5406830
C	81.4514636	-29.9533990	-2.5406830
C	75.7629170	-42.3264391	-2.5406830
C	68.2088359	-53.6572618	-2.5406830
C	58.9752269	-63.6668646	-2.5406830
C	48.2894521	-72.1087780	-2.5406830
C	36.4146307	-78.7751338	-2.5406830
C	23.6431603	-83.5017845	-2.5406830
C	10.2895167	-86.1723441	-2.5406830
C	-3.3174889	-86.7210546	-2.5406830
C	-16.8428068	-85.1344049	-2.5406830
C	-29.9533990	-81.4514636	-2.5406830
C	-42.3264391	-75.7629170	-2.5406830
C	-53.6572618	-68.2088359	-2.5406830
C	-63.6668646	-58.9752269	-2.5406830
C	-72.1087780	-48.2894521	-2.5406830
C	-78.7751338	-36.4146307	-2.5406830
C	-83.5017845	-23.6431603	-2.5406830
C	-86.1723441	-10.2895167	-2.5406830
C	-86.7210546	3.3174889	-2.5406830
C	-85.1344049	16.8428068	-2.5406830
C	-81.4514636	29.9533990	-2.5406830
C	-75.7629170	42.3264391	-2.5406830
C	-68.2088359	53.6572618	-2.5406830
C	-58.9752269	63.6668646	-2.5406830
C	-48.2894521	72.1087780	-2.5406830
C	-36.4146307	78.7751338	-2.5406830
C	-23.6431603	83.5017845	-2.5406830
C	-10.2895167	86.1723441	-2.5406830
C	3.3174889	86.7210546	-2.5406830
C	16.8428068	85.1344049	-2.5406830
C	29.9533990	81.4514636	-2.5406830
C	42.3264391	75.7629170	-2.5406830
C	53.6572618	68.2088359	-2.5406830
C	63.6668646	58.9752269	-2.5406830
C	72.1087780	48.2894521	-2.5406830
C	78.7751338	36.4146307	-2.5406830
C	83.5017845	23.6431603	-2.5406830
C	86.1723441	-10.2895167	-2.5406830
C	86.7210546	3.3174889	-2.5406830
C	85.1344049	16.8428068	-2.5406830
C	81.4514636	29.9533990	-2.5406830
C	75.7629170	42.3264391	-2.5406830
C	68.2088359	53.6572618	-2.5406830
C	58.9752269	63.6668646	-2.5406830
C	48.2894521	72.1087780	-2.5406830
C	36.4146307	78.7751338	-2.5406830

C	23.6431603	83.5017845	-2.5406830
C	10.2895167	86.1723441	-2.5406830
C	-3.3174889	86.7210546	-2.5406830
C	-16.8428068	85.1344049	-2.5406830
C	-29.9533990	81.4514636	-2.5406830
C	-42.3264391	75.7629170	-2.5406830
C	-53.6572618	68.2088359	-2.5406830
C	-63.6668646	58.9752269	-2.5406830
C	-72.1087780	48.2894521	-2.5406830
C	-78.7751338	36.4146307	-2.5406830
C	-83.5017845	23.6431603	-2.5406830
C	-86.1723441	10.2895167	-2.5406830
C	-86.7210546	-3.3174889	-2.5406830
C	-85.1344049	-16.8428068	-2.5406830
C	-81.4514636	-29.9533990	-2.5406830
C	-75.7629170	-42.3264391	-2.5406830
C	-68.2088359	-53.6572618	-2.5406830
C	-58.9752269	-63.6668646	-2.5406830
C	-48.2894521	-72.1087780	-2.5406830
C	-36.4146307	-78.7751338	-2.5406830
C	-23.6431603	-83.5017845	-2.5406830
C	-10.2895167	-86.1723441	-2.5406830
C	3.3174889	-86.7210546	-2.5406830
C	16.8428068	-85.1344049	-2.5406830
C	29.9533990	-81.4514636	-2.5406830
C	42.3264391	-75.7629170	-2.5406830
C	53.6572618	-68.2088359	-2.5406830
C	63.6668646	-58.9752269	-2.5406830
C	72.1087780	-48.2894521	-2.5406830
C	78.7751338	-36.4146307	-2.5406830
C	83.5017845	-23.6431603	-2.5406830
C	86.1723441	10.2895167	2.5406830
C	85.1344049	-16.8428068	2.5406830
C	81.4514636	-29.9533990	2.5406830
C	75.7629170	-42.3264391	2.5406830
C	68.2088359	-53.6572618	2.5406830
C	58.9752269	-63.6668646	2.5406830
C	48.2894521	-72.1087780	2.5406830
C	36.4146307	-78.7751338	2.5406830
C	23.6431603	-83.5017845	2.5406830
C	10.2895167	-86.1723441	2.5406830
C	-3.3174889	-86.7210546	2.5406830
C	-16.8428068	-85.1344049	2.5406830
C	-29.9533990	-81.4514636	2.5406830
C	-42.3264391	-75.7629170	2.5406830
C	-53.6572618	-68.2088359	2.5406830
C	-63.6668646	-58.9752269	2.5406830
C	-72.1087780	-48.2894521	2.5406830
C	-78.7751338	-36.4146307	2.5406830

C	-83.5017845	-23.6431603	2.5406830
C	-86.1723441	-10.2895167	2.5406830
C	-86.7210546	3.3174889	2.5406830
C	-85.1344049	16.8428068	2.5406830
C	-81.4514636	29.9533990	2.5406830
C	-75.7629170	42.3264391	2.5406830
C	-68.2088359	53.6572618	2.5406830
C	-58.9752269	63.6668646	2.5406830
C	-48.2894521	72.1087780	2.5406830
C	-36.4146307	78.7751338	2.5406830
C	-23.6431603	83.5017845	2.5406830
C	-10.2895167	86.1723441	2.5406830
C	3.3174889	86.7210546	2.5406830
C	16.8428068	85.1344049	2.5406830
C	29.9533990	81.4514636	2.5406830
C	42.3264391	75.7629170	2.5406830
C	53.6572618	68.2088359	2.5406830
C	63.6668646	58.9752269	2.5406830
C	72.1087780	48.2894521	2.5406830
C	78.7751338	36.4146307	2.5406830
C	83.5017845	23.6431603	2.5406830
C	86.6733817	4.2951210	3.4960576
C	84.9343836	17.8009450	3.4960576
C	81.1040191	30.8684507	3.4960576
C	75.2766044	43.1758727	3.4960576
C	67.5956300	54.4201614	3.4960576
C	58.2502268	64.3244451	3.4960576
C	47.4705097	72.6448475	3.4960576
C	35.5219111	79.1764927	3.4960576
C	22.6986451	83.7585498	3.4960576
C	9.3164632	86.2781935	3.4960576
C	-4.2951210	86.6733817	3.4960576
C	-17.8009450	84.9343836	3.4960576
C	-30.8684507	81.1040191	3.4960576
C	-43.1758727	75.2766044	3.4960576
C	-54.4201614	67.5956300	3.4960576
C	-64.3244451	58.2502268	3.4960576
C	-72.6448475	47.4705097	3.4960576
C	-79.1764927	35.5219111	3.4960576
C	-83.7585498	22.6986451	3.4960576
C	-86.2781935	9.3164632	3.4960576
C	-86.6733817	-4.2951210	3.4960576
C	-84.9343836	-17.8009450	3.4960576
C	-81.1040191	-30.8684507	3.4960576
C	-75.2766044	-43.1758727	3.4960576
C	-67.5956300	-54.4201614	3.4960576
C	-58.2502268	-64.3244451	3.4960576
C	-47.4705097	-72.6448475	3.4960576
C	-35.5219111	-79.1764927	3.4960576

C	-22.6986451	-83.7585498	3.4960576
C	-9.3164632	-86.2781935	3.4960576
C	4.2951210	-86.6733817	3.4960576
C	17.8009450	-84.9343836	3.4960576
C	30.8684507	-81.1040191	3.4960576
C	43.1758727	-75.2766044	3.4960576
C	54.4201614	-67.5956300	3.4960576
C	64.3244451	-58.2502268	3.4960576
C	72.6448475	-47.4705097	3.4960576
C	79.1764927	-35.5219111	3.4960576
C	83.7585498	-22.6986451	3.4960576
C	86.2781935	9.3164632	-3.4960576
C	86.6733817	-4.2951210	-3.4960576
C	84.9343836	-17.8009450	-3.4960576
C	81.1040191	-30.8684507	-3.4960576
C	75.2766044	-43.1758727	-3.4960576
C	67.5956300	-54.4201614	-3.4960576
C	58.2502268	-64.3244451	-3.4960576
C	47.4705097	-72.6448475	-3.4960576
C	35.5219111	-79.1764927	-3.4960576
C	22.6986451	-83.7585498	-3.4960576
C	9.3164632	-86.2781935	-3.4960576
C	-4.2951210	-86.6733817	-3.4960576
C	-17.8009450	-84.9343836	-3.4960576
C	-30.8684507	-81.1040191	-3.4960576
C	-43.1758727	-75.2766044	-3.4960576
C	-54.4201614	-67.5956300	-3.4960576
C	-64.3244451	-58.2502268	-3.4960576
C	-72.6448475	-47.4705097	-3.4960576
C	-79.1764927	-35.5219111	-3.4960576
C	-83.7585498	-22.6986451	-3.4960576
C	-86.2781935	-9.3164632	-3.4960576
C	-86.6733817	4.2951210	-3.4960576
C	-84.9343836	17.8009450	-3.4960576
C	-81.1040191	30.8684507	-3.4960576
C	-75.2766044	43.1758727	-3.4960576
C	-67.5956300	54.4201614	-3.4960576
C	-58.2502268	64.3244451	-3.4960576
C	-47.4705097	72.6448475	-3.4960576
C	-35.5219111	79.1764927	-3.4960576
C	-22.6986451	83.7585498	-3.4960576
C	-9.3164632	86.2781935	-3.4960576
C	4.2951210	86.6733817	-3.4960576
C	17.8009450	84.9343836	-3.4960576
C	30.8684507	81.1040191	-3.4960576
C	43.1758727	75.2766044	-3.4960576
C	54.4201614	67.5956300	-3.4960576
C	64.3244451	58.2502268	-3.4960576
C	72.6448475	47.4705097	-3.4960576

C	79.1764927	35.5219111	-3.4960576
C	83.7585498	22.6986451	-3.4960576
C	86.2781935	-9.3164632	-3.4960576
C	86.6733817	4.2951210	-3.4960576
C	84.9343836	17.8009450	-3.4960576
C	81.1040191	30.8684507	-3.4960576
C	75.2766044	43.1758727	-3.4960576
C	67.5956300	54.4201614	-3.4960576
C	58.2502268	64.3244451	-3.4960576
C	47.4705097	72.6448475	-3.4960576
C	35.5219111	79.1764927	-3.4960576
C	22.6986451	83.7585498	-3.4960576
C	9.3164632	86.2781935	-3.4960576
C	-4.2951210	86.6733817	-3.4960576
C	-17.8009450	84.9343836	-3.4960576
C	-30.8684507	81.1040191	-3.4960576
C	-43.1758727	75.2766044	-3.4960576
C	-54.4201614	67.5956300	-3.4960576
C	-64.3244451	58.2502268	-3.4960576
C	-72.6448475	47.4705097	-3.4960576
C	-79.1764927	35.5219111	-3.4960576
C	-83.7585498	22.6986451	-3.4960576
C	-86.2781935	9.3164632	-3.4960576
C	-86.6733817	-4.2951210	-3.4960576
C	-84.9343836	-17.8009450	-3.4960576
C	-81.1040191	-30.8684507	-3.4960576
C	-75.2766044	-43.1758727	-3.4960576
C	-67.5956300	-54.4201614	-3.4960576
C	-58.2502268	-64.3244451	-3.4960576
C	-47.4705097	-72.6448475	-3.4960576
C	-35.5219111	-79.1764927	-3.4960576
C	-22.6986451	-83.7585498	-3.4960576
C	-9.3164632	-86.2781935	-3.4960576
C	4.2951210	-86.6733817	-3.4960576
C	17.8009450	-84.9343836	-3.4960576
C	30.8684507	-81.1040191	-3.4960576
C	43.1758727	-75.2766044	-3.4960576
C	54.4201614	-67.5956300	-3.4960576
C	64.3244451	-58.2502268	-3.4960576
C	72.6448475	-47.4705097	-3.4960576
C	79.1764927	-35.5219111	-3.4960576
C	83.7585498	-22.6986451	-3.4960576
C	86.2781935	9.3164632	3.4960576
C	84.9343836	-17.8009450	3.4960576
C	81.1040191	-30.8684507	3.4960576
C	75.2766044	-43.1758727	3.4960576
C	67.5956300	-54.4201614	3.4960576
C	58.2502268	-64.3244451	3.4960576
C	47.4705097	-72.6448475	3.4960576

C	35.5219111	-79.1764927	3.4960576
C	22.6986451	-83.7585498	3.4960576
C	9.3164632	-86.2781935	3.4960576
C	-4.2951210	-86.6733817	3.4960576
C	-17.8009450	-84.9343836	3.4960576
C	-30.8684507	-81.1040191	3.4960576
C	-43.1758727	-75.2766044	3.4960576
C	-54.4201614	-67.5956300	3.4960576
C	-64.3244451	-58.2502268	3.4960576
C	-72.6448475	-47.4705097	3.4960576
C	-79.1764927	-35.5219111	3.4960576
C	-83.7585498	-22.6986451	3.4960576
C	-86.2781935	-9.3164632	3.4960576
C	-86.6733817	4.2951210	3.4960576
C	-84.9343836	17.8009450	3.4960576
C	-81.1040191	30.8684507	3.4960576
C	-75.2766044	43.1758727	3.4960576
C	-67.5956300	54.4201614	3.4960576
C	-58.2502268	64.3244451	3.4960576
C	-47.4705097	72.6448475	3.4960576
C	-35.5219111	79.1764927	3.4960576
C	-22.6986451	83.7585498	3.4960576
C	-9.3164632	86.2781935	3.4960576
C	4.2951210	86.6733817	3.4960576
C	17.8009450	84.9343836	3.4960576
C	30.8684507	81.1040191	3.4960576
C	43.1758727	75.2766044	3.4960576
C	54.4201614	67.5956300	3.4960576
C	64.3244451	58.2502268	3.4960576
C	72.6448475	47.4705097	3.4960576
C	79.1764927	35.5219111	3.4960576
C	83.7585498	22.6986451	3.4960576
C	86.5843049	5.5582567	2.7983138
C	84.6488055	19.0345948	2.7983138
C	80.6289716	32.0422380	2.7983138
C	74.6237848	44.2608949	2.7983138
C	66.7811128	55.3897017	2.7983138
C	57.2940681	65.1546302	2.7983138
C	46.3962533	73.3152355	2.7983138
C	34.3560088	79.6705763	2.7983138
C	21.4698054	84.0641632	2.7983138
C	8.0549440	86.3878114	2.7983138
C	-5.5582567	86.5843049	2.7983138
C	-19.0345948	84.6488055	2.7983138
C	-32.0422380	80.6289716	2.7983138
C	-44.2608949	74.6237848	2.7983138
C	-55.3897017	66.7811128	2.7983138
C	-65.1546302	57.2940681	2.7983138
C	-73.3152355	46.3962533	2.7983138

C	-79.6705763	34.3560088	2.7983138
C	-84.0641632	21.4698054	2.7983138
C	-86.3878114	8.0549440	2.7983138
C	-86.5843049	-5.5582567	2.7983138
C	-84.6488055	-19.0345948	2.7983138
C	-80.6289716	-32.0422380	2.7983138
C	-74.6237848	-44.2608949	2.7983138
C	-66.7811128	-55.3897017	2.7983138
C	-57.2940681	-65.1546302	2.7983138
C	-46.3962533	-73.3152355	2.7983138
C	-34.3560088	-79.6705763	2.7983138
C	-21.4698054	-84.0641632	2.7983138
C	-8.0549440	-86.3878114	2.7983138
C	5.5582567	-86.5843049	2.7983138
C	19.0345948	-84.6488055	2.7983138
C	32.0422380	-80.6289716	2.7983138
C	44.2608949	-74.6237848	2.7983138
C	55.3897017	-66.7811128	2.7983138
C	65.1546302	-57.2940681	2.7983138
C	73.3152355	-46.3962533	2.7983138
C	79.6705763	-34.3560088	2.7983138
C	84.0641632	-21.4698054	2.7983138
C	86.3878114	8.0549440	-2.7983138
C	86.5843049	-5.5582567	-2.7983138
C	84.6488055	-19.0345948	-2.7983138
C	80.6289716	-32.0422380	-2.7983138
C	74.6237848	-44.2608949	-2.7983138
C	66.7811128	-55.3897017	-2.7983138
C	57.2940681	-65.1546302	-2.7983138
C	46.3962533	-73.3152355	-2.7983138
C	34.3560088	-79.6705763	-2.7983138
C	21.4698054	-84.0641632	-2.7983138
C	8.0549440	-86.3878114	-2.7983138
C	-5.5582567	-86.5843049	-2.7983138
C	-19.0345948	-84.6488055	-2.7983138
C	-32.0422380	-80.6289716	-2.7983138
C	-44.2608949	-74.6237848	-2.7983138
C	-55.3897017	-66.7811128	-2.7983138
C	-65.1546302	-57.2940681	-2.7983138
C	-73.3152355	-46.3962533	-2.7983138
C	-79.6705763	-34.3560088	-2.7983138
C	-84.0641632	-21.4698054	-2.7983138
C	-86.3878114	-8.0549440	-2.7983138
C	-86.5843049	5.5582567	-2.7983138
C	-84.6488055	19.0345948	-2.7983138
C	-80.6289716	32.0422380	-2.7983138
C	-74.6237848	44.2608949	-2.7983138
C	-66.7811128	55.3897017	-2.7983138
C	-57.2940681	65.1546302	-2.7983138

C	-46.3962533	73.3152355	-2.7983138
C	-34.3560088	79.6705763	-2.7983138
C	-21.4698054	84.0641632	-2.7983138
C	-8.0549440	86.3878114	-2.7983138
C	5.5582567	86.5843049	-2.7983138
C	19.0345948	84.6488055	-2.7983138
C	32.0422380	80.6289716	-2.7983138
C	44.2608949	74.6237848	-2.7983138
C	55.3897017	66.7811128	-2.7983138
C	65.1546302	57.2940681	-2.7983138
C	73.3152355	46.3962533	-2.7983138
C	79.6705763	34.3560088	-2.7983138
C	84.0641632	21.4698054	-2.7983138
C	86.3878114	-8.0549440	-2.7983138
C	86.5843049	5.5582567	-2.7983138
C	84.6488055	19.0345948	-2.7983138
C	80.6289716	32.0422380	-2.7983138
C	74.6237848	44.2608949	-2.7983138
C	66.7811128	55.3897017	-2.7983138
C	57.2940681	65.1546302	-2.7983138
C	46.3962533	73.3152355	-2.7983138
C	34.3560088	79.6705763	-2.7983138
C	21.4698054	84.0641632	-2.7983138
C	8.0549440	86.3878114	-2.7983138
C	-5.5582567	86.5843049	-2.7983138
C	-19.0345948	84.6488055	-2.7983138
C	-32.0422380	80.6289716	-2.7983138
C	-44.2608949	74.6237848	-2.7983138
C	-55.3897017	66.7811128	-2.7983138
C	-65.1546302	57.2940681	-2.7983138
C	-73.3152355	46.3962533	-2.7983138
C	-79.6705763	34.3560088	-2.7983138
C	-84.0641632	21.4698054	-2.7983138
C	-86.3878114	8.0549440	-2.7983138
C	-86.5843049	-5.5582567	-2.7983138
C	-84.6488055	-19.0345948	-2.7983138
C	-80.6289716	-32.0422380	-2.7983138
C	-74.6237848	-44.2608949	-2.7983138
C	-66.7811128	-55.3897017	-2.7983138
C	-57.2940681	-65.1546302	-2.7983138
C	-46.3962533	-73.3152355	-2.7983138
C	-34.3560088	-79.6705763	-2.7983138
C	-21.4698054	-84.0641632	-2.7983138
C	-8.0549440	-86.3878114	-2.7983138
C	5.5582567	-86.5843049	-2.7983138
C	19.0345948	-84.6488055	-2.7983138
C	32.0422380	-80.6289716	-2.7983138
C	44.2608949	-74.6237848	-2.7983138
C	55.3897017	-66.7811128	-2.7983138

C	65.1546302	-57.2940681	-2.7983138
C	73.3152355	-46.3962533	-2.7983138
C	79.6705763	-34.3560088	-2.7983138
C	84.0641632	-21.4698054	-2.7983138
C	86.3878114	8.0549440	2.7983138
C	84.6488055	-19.0345948	2.7983138
C	80.6289716	-32.0422380	2.7983138
C	74.6237848	-44.2608949	2.7983138
C	66.7811128	-55.3897017	2.7983138
C	57.2940681	-65.1546302	2.7983138
C	46.3962533	-73.3152355	2.7983138
C	34.3560088	-79.6705763	2.7983138
C	21.4698054	-84.0641632	2.7983138
C	8.0549440	-86.3878114	2.7983138
C	-5.5582567	-86.5843049	2.7983138
C	-19.0345948	-84.6488055	2.7983138
C	-32.0422380	-80.6289716	2.7983138
C	-44.2608949	-74.6237848	2.7983138
C	-55.3897017	-66.7811128	2.7983138
C	-65.1546302	-57.2940681	2.7983138
C	-73.3152355	-46.3962533	2.7983138
C	-79.6705763	-34.3560088	2.7983138
C	-84.0641632	-21.4698054	2.7983138
C	-86.3878114	-8.0549440	2.7983138
C	-86.5843049	5.5582567	2.7983138
C	-84.6488055	19.0345948	2.7983138
C	-80.6289716	32.0422380	2.7983138
C	-74.6237848	44.2608949	2.7983138
C	-66.7811128	55.3897017	2.7983138
C	-57.2940681	65.1546302	2.7983138
C	-46.3962533	73.3152355	2.7983138
C	-34.3560088	79.6705763	2.7983138
C	-21.4698054	84.0641632	2.7983138
C	-8.0549440	86.3878114	2.7983138
C	5.5582567	86.5843049	2.7983138
C	19.0345948	84.6488055	2.7983138
C	32.0422380	80.6289716	2.7983138
C	44.2608949	74.6237848	2.7983138
C	55.3897017	66.7811128	2.7983138
C	65.1546302	57.2940681	2.7983138
C	73.3152355	46.3962533	2.7983138
C	79.6705763	34.3560088	2.7983138
C	84.0641632	21.4698054	2.7983138
N	86.5845744	5.3370239	1.4413028
N	84.6836801	18.8161278	1.4413028
N	80.6975926	31.8319163	1.4413028
N	74.7244626	44.0638973	1.4413028
N	66.9113682	55.2108789	1.4413028
N	57.4506939	64.9983855	1.4413028

N	46.5753929	73.1854161	1.4413028
N	34.5532511	79.5703788	1.4413028
N	21.6802936	83.9960548	1.4413028
N	8.2734953	86.3534691	1.4413028
N	-5.3370239	86.5845744	1.4413028
N	-18.8161278	84.6836801	1.4413028
N	-31.8319163	80.6975926	1.4413028
N	-44.0638973	74.7244626	1.4413028
N	-55.2108789	66.9113682	1.4413028
N	-64.9983855	57.4506939	1.4413028
N	-73.1854161	46.5753929	1.4413028
N	-79.5703788	34.5532511	1.4413028
N	-83.9960548	21.6802936	1.4413028
N	-86.3534691	8.2734953	1.4413028
N	-86.5845744	-5.3370239	1.4413028
N	-84.6836801	-18.8161278	1.4413028
N	-80.6975926	-31.8319163	1.4413028
N	-74.7244626	-44.0638973	1.4413028
N	-66.9113682	-55.2108789	1.4413028
N	-57.4506939	-64.9983855	1.4413028
N	-46.5753929	-73.1854161	1.4413028
N	-34.5532511	-79.5703788	1.4413028
N	-21.6802936	-83.9960548	1.4413028
N	-8.2734953	-86.3534691	1.4413028
N	5.3370239	-86.5845744	1.4413028
N	18.8161278	-84.6836801	1.4413028
N	31.8319163	-80.6975926	1.4413028
N	44.0638973	-74.7244626	1.4413028
N	55.2108789	-66.9113682	1.4413028
N	64.9983855	-57.4506939	1.4413028
N	73.1854161	-46.5753929	1.4413028
N	79.5703788	-34.5532511	1.4413028
N	83.9960548	-21.6802936	1.4413028
N	86.3534691	8.2734953	-1.4413028
N	86.5845744	-5.3370239	-1.4413028
N	84.6836801	-18.8161278	-1.4413028
N	80.6975926	-31.8319163	-1.4413028
N	74.7244626	-44.0638973	-1.4413028
N	66.9113682	-55.2108789	-1.4413028
N	57.4506939	-64.9983855	-1.4413028
N	46.5753929	-73.1854161	-1.4413028
N	34.5532511	-79.5703788	-1.4413028
N	21.6802936	-83.9960548	-1.4413028
N	8.2734953	-86.3534691	-1.4413028
N	-5.3370239	-86.5845744	-1.4413028
N	-18.8161278	-84.6836801	-1.4413028
N	-31.8319163	-80.6975926	-1.4413028
N	-44.0638973	-74.7244626	-1.4413028
N	-55.2108789	-66.9113682	-1.4413028

N	-64.9983855	-57.4506939	-1.4413028
N	-73.1854161	-46.5753929	-1.4413028
N	-79.5703788	-34.5532511	-1.4413028
N	-83.9960548	-21.6802936	-1.4413028
N	-86.3534691	-8.2734953	-1.4413028
N	-86.5845744	5.3370239	-1.4413028
N	-84.6836801	18.8161278	-1.4413028
N	-80.6975926	31.8319163	-1.4413028
N	-74.7244626	44.0638973	-1.4413028
N	-66.9113682	55.2108789	-1.4413028
N	-57.4506939	64.9983855	-1.4413028
N	-46.5753929	73.1854161	-1.4413028
N	-34.5532511	79.5703788	-1.4413028
N	-21.6802936	83.9960548	-1.4413028
N	-8.2734953	86.3534691	-1.4413028
N	5.3370239	86.5845744	-1.4413028
N	18.8161278	84.6836801	-1.4413028
N	31.8319163	80.6975926	-1.4413028
N	44.0638973	74.7244626	-1.4413028
N	55.2108789	66.9113682	-1.4413028
N	64.9983855	57.4506939	-1.4413028
N	73.1854161	46.5753929	-1.4413028
N	79.5703788	34.5532511	-1.4413028
N	83.9960548	21.6802936	-1.4413028
N	86.3534691	-8.2734953	-1.4413028
N	86.5845744	5.3370239	-1.4413028
N	84.6836801	18.8161278	-1.4413028
N	80.6975926	31.8319163	-1.4413028
N	74.7244626	44.0638973	-1.4413028
N	66.9113682	55.2108789	-1.4413028
N	57.4506939	64.9983855	-1.4413028
N	46.5753929	73.1854161	-1.4413028
N	34.5532511	79.5703788	-1.4413028
N	21.6802936	83.9960548	-1.4413028
N	8.2734953	86.3534691	-1.4413028
N	-5.3370239	86.5845744	-1.4413028
N	-18.8161278	84.6836801	-1.4413028
N	-31.8319163	80.6975926	-1.4413028
N	-44.0638973	74.7244626	-1.4413028
N	-55.2108789	66.9113682	-1.4413028
N	-64.9983855	57.4506939	-1.4413028
N	-73.1854161	46.5753929	-1.4413028
N	-79.5703788	34.5532511	-1.4413028
N	-83.9960548	21.6802936	-1.4413028
N	-86.3534691	8.2734953	-1.4413028
N	-86.5845744	-5.3370239	-1.4413028
N	-84.6836801	-18.8161278	-1.4413028
N	-80.6975926	-31.8319163	-1.4413028
N	-74.7244626	-44.0638973	-1.4413028

N	-66.9113682	-55.2108789	-1.4413028
N	-57.4506939	-64.9983855	-1.4413028
N	-46.5753929	-73.1854161	-1.4413028
N	-34.5532511	-79.5703788	-1.4413028
N	-21.6802936	-83.9960548	-1.4413028
N	-8.2734953	-86.3534691	-1.4413028
N	5.3370239	-86.5845744	-1.4413028
N	18.8161278	-84.6836801	-1.4413028
N	31.8319163	-80.6975926	-1.4413028
N	44.0638973	-74.7244626	-1.4413028
N	55.2108789	-66.9113682	-1.4413028
N	64.9983855	-57.4506939	-1.4413028
N	73.1854161	-46.5753929	-1.4413028
N	79.5703788	-34.5532511	-1.4413028
N	83.9960548	-21.6802936	-1.4413028
N	86.3534691	8.2734953	1.4413028
N	84.6836801	-18.8161278	1.4413028
N	80.6975926	-31.8319163	1.4413028
N	74.7244626	-44.0638973	1.4413028
N	66.9113682	-55.2108789	1.4413028
N	57.4506939	-64.9983855	1.4413028
N	46.5753929	-73.1854161	1.4413028
N	34.5532511	-79.5703788	1.4413028
N	21.6802936	-83.9960548	1.4413028
N	8.2734953	-86.3534691	1.4413028
N	-5.3370239	-86.5845744	1.4413028
N	-18.8161278	-84.6836801	1.4413028
N	-31.8319163	-80.6975926	1.4413028
N	-44.0638973	-74.7244626	1.4413028
N	-55.2108789	-66.9113682	1.4413028
N	-64.9983855	-57.4506939	1.4413028
N	-73.1854161	-46.5753929	1.4413028
N	-79.5703788	-34.5532511	1.4413028
N	-83.9960548	-21.6802936	1.4413028
N	-86.3534691	-8.2734953	1.4413028
N	-86.5845744	5.3370239	1.4413028
N	-84.6836801	18.8161278	1.4413028
N	-80.6975926	31.8319163	1.4413028
N	-74.7244626	44.0638973	1.4413028
N	-66.9113682	55.2108789	1.4413028
N	-57.4506939	64.9983855	1.4413028
N	-46.5753929	73.1854161	1.4413028
N	-34.5532511	79.5703788	1.4413028
N	-21.6802936	83.9960548	1.4413028
N	-8.2734953	86.3534691	1.4413028
N	5.3370239	86.5845744	1.4413028
N	18.8161278	84.6836801	1.4413028
N	31.8319163	80.6975926	1.4413028
N	44.0638973	74.7244626	1.4413028

N	55.2108789	66.9113682	1.4413028
N	64.9983855	57.4506939	1.4413028
N	73.1854161	46.5753929	1.4413028
N	79.5703788	34.5532511	1.4413028
N	83.9960548	21.6802936	1.4413028
C	86.4988001	6.8076032	3.4162659
C	84.3689126	20.2551838	3.4162659
C	80.1615824	33.2040146	3.4162659
C	73.9804080	45.3352523	3.4162659
C	65.9775905	56.3501857	3.4162659
C	56.3501857	65.9775905	3.4162659
C	45.3352523	73.9804080	3.4162659
C	33.2040146	80.1615824	3.4162659
C	20.2551838	84.3689126	3.4162659
C	6.8076032	86.4988001	3.4162659
C	-6.8076032	86.4988001	3.4162659
C	-20.2551838	84.3689126	3.4162659
C	-33.2040146	80.1615824	3.4162659
C	-45.3352523	73.9804080	3.4162659
C	-56.3501857	65.9775905	3.4162659
C	-65.9775905	56.3501857	3.4162659
C	-73.9804080	45.3352523	3.4162659
C	-80.1615824	33.2040146	3.4162659
C	-84.3689126	20.2551838	3.4162659
C	-86.4988001	6.8076032	3.4162659
C	-86.4988001	-6.8076032	3.4162659
C	-84.3689126	-20.2551838	3.4162659
C	-80.1615824	-33.2040146	3.4162659
C	-73.9804080	-45.3352523	3.4162659
C	-65.9775905	-56.3501857	3.4162659
C	-56.3501857	-65.9775905	3.4162659
C	-45.3352523	-73.9804080	3.4162659
C	-33.2040146	-80.1615824	3.4162659
C	-20.2551838	-84.3689126	3.4162659
C	-6.8076032	-86.4988001	3.4162659
C	6.8076032	-86.4988001	3.4162659
C	20.2551838	-84.3689126	3.4162659
C	33.2040146	-80.1615824	3.4162659
C	45.3352523	-73.9804080	3.4162659
C	56.3501857	-65.9775905	3.4162659
C	65.9775905	-56.3501857	3.4162659
C	73.9804080	-45.3352523	3.4162659
C	80.1615824	-33.2040146	3.4162659
C	84.3689126	-20.2551838	3.4162659
C	86.4988001	6.8076032	-3.4162659
C	86.4988001	-6.8076032	-3.4162659
C	84.3689126	-20.2551838	-3.4162659
C	80.1615824	-33.2040146	-3.4162659
C	73.9804080	-45.3352523	-3.4162659

C	65.9775905	-56.3501857	-3.4162659
C	56.3501857	-65.9775905	-3.4162659
C	45.3352523	-73.9804080	-3.4162659
C	33.2040146	-80.1615824	-3.4162659
C	20.2551838	-84.3689126	-3.4162659
C	6.8076032	-86.4988001	-3.4162659
C	-6.8076032	-86.4988001	-3.4162659
C	-20.2551838	-84.3689126	-3.4162659
C	-33.2040146	-80.1615824	-3.4162659
C	-45.3352523	-73.9804080	-3.4162659
C	-56.3501857	-65.9775905	-3.4162659
C	-65.9775905	-56.3501857	-3.4162659
C	-73.9804080	-45.3352523	-3.4162659
C	-80.1615824	-33.2040146	-3.4162659
C	-84.3689126	-20.2551838	-3.4162659
C	-86.4988001	-6.8076032	-3.4162659
C	-86.4988001	6.8076032	-3.4162659
C	-84.3689126	20.2551838	-3.4162659
C	-80.1615824	33.2040146	-3.4162659
C	-73.9804080	45.3352523	-3.4162659
C	-65.9775905	56.3501857	-3.4162659
C	-56.3501857	65.9775905	-3.4162659
C	-45.3352523	73.9804080	-3.4162659
C	-33.2040146	80.1615824	-3.4162659
C	-20.2551838	84.3689126	-3.4162659
C	-6.8076032	86.4988001	-3.4162659
C	6.8076032	86.4988001	-3.4162659
C	20.2551838	84.3689126	-3.4162659
C	33.2040146	80.1615824	-3.4162659
C	45.3352523	73.9804080	-3.4162659
C	56.3501857	65.9775905	-3.4162659
C	65.9775905	56.3501857	-3.4162659
C	73.9804080	45.3352523	-3.4162659
C	80.1615824	33.2040146	-3.4162659
C	84.3689126	20.2551838	-3.4162659
H	86.5106341	6.8085346	4.5085013
H	84.3804552	20.2579550	4.5085013
H	80.1725494	33.2085573	4.5085013
H	73.9905294	45.3414547	4.5085013
H	65.9866170	56.3578951	4.5085013
H	56.3578951	65.9866170	4.5085013
H	45.3414547	73.9905294	4.5085013
H	33.2085573	80.1725494	4.5085013
H	20.2579550	84.3804552	4.5085013
H	6.8085346	86.5106341	4.5085013
H	-6.8085346	86.5106341	4.5085013
H	-20.2579550	84.3804552	4.5085013
H	-33.2085573	80.1725494	4.5085013
H	-45.3414547	73.9905294	4.5085013

H	-56.3578951	65.9866170	4.5085013
H	-65.9866170	56.3578951	4.5085013
H	-73.9905294	45.3414547	4.5085013
H	-80.1725494	33.2085573	4.5085013
H	-84.3804552	20.2579550	4.5085013
H	-86.5106341	6.8085346	4.5085013
H	-86.5106341	-6.8085346	4.5085013
H	-84.3804552	-20.2579550	4.5085013
H	-80.1725494	-33.2085573	4.5085013
H	-73.9905294	-45.3414547	4.5085013
H	-65.9866170	-56.3578951	4.5085013
H	-56.3578951	-65.9866170	4.5085013
H	-45.3414547	-73.9905294	4.5085013
H	-33.2085573	-80.1725494	4.5085013
H	-20.2579550	-84.3804552	4.5085013
H	-6.8085346	-86.5106341	4.5085013
H	6.8085346	-86.5106341	4.5085013
H	20.2579550	-84.3804552	4.5085013
H	33.2085573	-80.1725494	4.5085013
H	45.3414547	-73.9905294	4.5085013
H	56.3578951	-65.9866170	4.5085013
H	65.9866170	-56.3578951	4.5085013
H	73.9905294	-45.3414547	4.5085013
H	80.1725494	-33.2085573	4.5085013
H	84.3804552	-20.2579550	4.5085013
H	86.5106341	6.8085346	-4.5085013
H	86.5106341	-6.8085346	-4.5085013
H	84.3804552	-20.2579550	-4.5085013
H	80.1725494	-33.2085573	-4.5085013
H	73.9905294	-45.3414547	-4.5085013
H	65.9866170	-56.3578951	-4.5085013
H	56.3578951	-65.9866170	-4.5085013
H	45.3414547	-73.9905294	-4.5085013
H	33.2085573	-80.1725494	-4.5085013
H	20.2579550	-84.3804552	-4.5085013
H	6.8085346	-86.5106341	-4.5085013
H	-6.8085346	-86.5106341	-4.5085013
H	-20.2579550	-84.3804552	-4.5085013
H	-33.2085573	-80.1725494	-4.5085013
H	-45.3414547	-73.9905294	-4.5085013
H	-56.3578951	-65.9866170	-4.5085013
H	-65.9866170	-56.3578951	-4.5085013
H	-73.9905294	-45.3414547	-4.5085013
H	-80.1725494	-33.2085573	-4.5085013
H	-84.3804552	-20.2579550	-4.5085013
H	-86.5106341	-6.8085346	-4.5085013
H	-86.5106341	6.8085346	-4.5085013
H	-84.3804552	20.2579550	-4.5085013
H	-80.1725494	33.2085573	-4.5085013

H	-73.9905294	45.3414547	-4.5085013
H	-65.9866170	56.3578951	-4.5085013
H	-56.3578951	65.9866170	-4.5085013
H	-45.3414547	73.9905294	-4.5085013
H	-33.2085573	80.1725494	-4.5085013
H	-20.2579550	84.3804552	-4.5085013
H	-6.8085346	86.5106341	-4.5085013
H	6.8085346	86.5106341	-4.5085013
H	20.2579550	84.3804552	-4.5085013
H	33.2085573	80.1725494	-4.5085013
H	45.3414547	73.9905294	-4.5085013
H	56.3578951	65.9866170	-4.5085013
H	65.9866170	56.3578951	-4.5085013
H	73.9905294	45.3414547	-4.5085013
H	80.1725494	33.2085573	-4.5085013
H	84.3804552	20.2579550	-4.5085013
H	86.7892879	2.2409441	2.6782730
H	85.3702069	15.7901902	2.6782730
H	81.8490280	28.9506294	2.6782730
H	76.3124545	41.3982080	2.6782730
H	68.8968150	52.8264253	2.6782730
H	59.7847073	62.9538808	2.6782730
H	49.2005017	71.5312028	2.6782730
H	37.4048164	78.3471891	2.6782730
H	24.6881004	83.2340076	2.6782730
H	11.3634815	86.0713287	2.6782730
H	-2.2409441	86.7892879	2.6782730
H	-15.7901902	85.3702069	2.6782730
H	-28.9506294	81.8490280	2.6782730
H	-41.3982080	76.3124545	2.6782730
H	-52.8264253	68.8968150	2.6782730
H	-62.9538808	59.7847073	2.6782730
H	-71.5312028	49.2005017	2.6782730
H	-78.3471891	37.4048164	2.6782730
H	-83.2340076	24.6881004	2.6782730
H	-86.0713287	11.3634815	2.6782730
H	-86.7892879	-2.2409441	2.6782730
H	-85.3702069	-15.7901902	2.6782730
H	-81.8490280	-28.9506294	2.6782730
H	-76.3124545	-41.3982080	2.6782730
H	-68.8968150	-52.8264253	2.6782730
H	-59.7847073	-62.9538808	2.6782730
H	-49.2005017	-71.5312028	2.6782730
H	-37.4048164	-78.3471891	2.6782730
H	-24.6881004	-83.2340076	2.6782730
H	-11.3634815	-86.0713287	2.6782730
H	2.2409441	-86.7892879	2.6782730
H	15.7901902	-85.3702069	2.6782730
H	28.9506294	-81.8490280	2.6782730

H	41.3982080	-76.3124545	2.6782730
H	52.8264253	-68.8968150	2.6782730
H	62.9538808	-59.7847073	2.6782730
H	71.5312028	-49.2005017	2.6782730
H	78.3471891	-37.4048164	2.6782730
H	83.2340076	-24.6881004	2.6782730
H	86.0713287	11.3634815	-2.6782730
H	86.7892879	-2.2409441	-2.6782730
H	85.3702069	-15.7901902	-2.6782730
H	81.8490280	-28.9506294	-2.6782730
H	76.3124545	-41.3982080	-2.6782730
H	68.8968150	-52.8264253	-2.6782730
H	59.7847073	-62.9538808	-2.6782730
H	49.2005017	-71.5312028	-2.6782730
H	37.4048164	-78.3471891	-2.6782730
H	24.6881004	-83.2340076	-2.6782730
H	11.3634815	-86.0713287	-2.6782730
H	-2.2409441	-86.7892879	-2.6782730
H	-15.7901902	-85.3702069	-2.6782730
H	-28.9506294	-81.8490280	-2.6782730
H	-41.3982080	-76.3124545	-2.6782730
H	-52.8264253	-68.8968150	-2.6782730
H	-62.9538808	-59.7847073	-2.6782730
H	-71.5312028	-49.2005017	-2.6782730
H	-78.3471891	-37.4048164	-2.6782730
H	-83.2340076	-24.6881004	-2.6782730
H	-86.0713287	-11.3634815	-2.6782730
H	-86.7892879	2.2409441	-2.6782730
H	-85.3702069	15.7901902	-2.6782730
H	-81.8490280	28.9506294	-2.6782730
H	-76.3124545	41.3982080	-2.6782730
H	-68.8968150	52.8264253	-2.6782730
H	-59.7847073	62.9538808	-2.6782730
H	-49.2005017	71.5312028	-2.6782730
H	-37.4048164	78.3471891	-2.6782730
H	-24.6881004	83.2340076	-2.6782730
H	-11.3634815	86.0713287	-2.6782730
H	2.2409441	86.7892879	-2.6782730
H	15.7901902	85.3702069	-2.6782730
H	28.9506294	81.8490280	-2.6782730
H	41.3982080	76.3124545	-2.6782730
H	52.8264253	68.8968150	-2.6782730
H	62.9538808	59.7847073	-2.6782730
H	71.5312028	49.2005017	-2.6782730
H	78.3471891	37.4048164	-2.6782730
H	83.2340076	24.6881004	-2.6782730
H	86.0713287	-11.3634815	-2.6782730
H	86.7892879	2.2409441	-2.6782730
H	85.3702069	15.7901902	-2.6782730

H	81.8490280	28.9506294	-2.6782730
H	76.3124545	41.3982080	-2.6782730
H	68.8968150	52.8264253	-2.6782730
H	59.7847073	62.9538808	-2.6782730
H	49.2005017	71.5312028	-2.6782730
H	37.4048164	78.3471891	-2.6782730
H	24.6881004	83.2340076	-2.6782730
H	11.3634815	86.0713287	-2.6782730
H	-2.2409441	86.7892879	-2.6782730
H	-15.7901902	85.3702069	-2.6782730
H	-28.9506294	81.8490280	-2.6782730
H	-41.3982080	76.3124545	-2.6782730
H	-52.8264253	68.8968150	-2.6782730
H	-62.9538808	59.7847073	-2.6782730
H	-71.5312028	49.2005017	-2.6782730
H	-78.3471891	37.4048164	-2.6782730
H	-83.2340076	24.6881004	-2.6782730
H	-86.0713287	11.3634815	-2.6782730
H	-86.7892879	-2.2409441	-2.6782730
H	-85.3702069	-15.7901902	-2.6782730
H	-81.8490280	-28.9506294	-2.6782730
H	-76.3124545	-41.3982080	-2.6782730
H	-68.8968150	-52.8264253	-2.6782730
H	-59.7847073	-62.9538808	-2.6782730
H	-49.2005017	-71.5312028	-2.6782730
H	-37.4048164	-78.3471891	-2.6782730
H	-24.6881004	-83.2340076	-2.6782730
H	-11.3634815	-86.0713287	-2.6782730
H	2.2409441	-86.7892879	-2.6782730
H	15.7901902	-85.3702069	-2.6782730
H	28.9506294	-81.8490280	-2.6782730
H	41.3982080	-76.3124545	-2.6782730
H	52.8264253	-68.8968150	-2.6782730
H	62.9538808	-59.7847073	-2.6782730
H	71.5312028	-49.2005017	-2.6782730
H	78.3471891	-37.4048164	-2.6782730
H	83.2340076	-24.6881004	-2.6782730
H	86.0713287	11.3634815	2.6782730
H	85.3702069	-15.7901902	2.6782730
H	81.8490280	-28.9506294	2.6782730
H	76.3124545	-41.3982080	2.6782730
H	68.8968150	-52.8264253	2.6782730
H	59.7847073	-62.9538808	2.6782730
H	49.2005017	-71.5312028	2.6782730
H	37.4048164	-78.3471891	2.6782730
H	24.6881004	-83.2340076	2.6782730
H	11.3634815	-86.0713287	2.6782730
H	-2.2409441	-86.7892879	2.6782730
H	-15.7901902	-85.3702069	2.6782730

H	-28.9506294	-81.8490280	2.6782730
H	-41.3982080	-76.3124545	2.6782730
H	-52.8264253	-68.8968150	2.6782730
H	-62.9538808	-59.7847073	2.6782730
H	-71.5312028	-49.2005017	2.6782730
H	-78.3471891	-37.4048164	2.6782730
H	-83.2340076	-24.6881004	2.6782730
H	-86.0713287	-11.3634815	2.6782730
H	-86.7892879	2.2409441	2.6782730
H	-85.3702069	15.7901902	2.6782730
H	-81.8490280	28.9506294	2.6782730
H	-76.3124545	41.3982080	2.6782730
H	-68.8968150	52.8264253	2.6782730
H	-59.7847073	62.9538808	2.6782730
H	-49.2005017	71.5312028	2.6782730
H	-37.4048164	78.3471891	2.6782730
H	-24.6881004	83.2340076	2.6782730
H	-11.3634815	86.0713287	2.6782730
H	2.2409441	86.7892879	2.6782730
H	15.7901902	85.3702069	2.6782730
H	28.9506294	81.8490280	2.6782730
H	41.3982080	76.3124545	2.6782730
H	52.8264253	68.8968150	2.6782730
H	62.9538808	59.7847073	2.6782730
H	71.5312028	49.2005017	2.6782730
H	78.3471891	37.4048164	2.6782730
H	83.2340076	24.6881004	2.6782730
H	86.6937744	4.1786035	4.5787072
H	84.9727526	17.6890521	4.5787072
H	81.1594196	30.7639377	4.5787072
H	75.3476724	43.0813129	4.5787072
H	67.6806153	54.3378833	4.5787072
H	58.3470369	64.2564746	4.5787072
H	47.5767609	72.5928583	4.5787072
H	35.6349870	79.1417649	4.5787072
H	22.8157616	83.7419386	4.5787072
H	9.4347363	86.2801078	4.5787072
H	-4.1786035	86.6937744	4.5787072
H	-17.6890521	84.9727526	4.5787072
H	-30.7639377	81.1594196	4.5787072
H	-43.0813129	75.3476724	4.5787072
H	-54.3378833	67.6806153	4.5787072
H	-64.2564746	58.3470369	4.5787072
H	-72.5928583	47.5767609	4.5787072
H	-79.1417649	35.6349870	4.5787072
H	-83.7419386	22.8157616	4.5787072
H	-86.2801078	9.4347363	4.5787072
H	-86.6937744	-4.1786035	4.5787072
H	-84.9727526	-17.6890521	4.5787072

H	-81.1594196	-30.7639377	4.5787072
H	-75.3476724	-43.0813129	4.5787072
H	-67.6806153	-54.3378833	4.5787072
H	-58.3470369	-64.2564746	4.5787072
H	-47.5767609	-72.5928583	4.5787072
H	-35.6349870	-79.1417649	4.5787072
H	-22.8157616	-83.7419386	4.5787072
H	-9.4347363	-86.2801078	4.5787072
H	4.1786035	-86.6937744	4.5787072
H	17.6890521	-84.9727526	4.5787072
H	30.7639377	-81.1594196	4.5787072
H	43.0813129	-75.3476724	4.5787072
H	54.3378833	-67.6806153	4.5787072
H	64.2564746	-58.3470369	4.5787072
H	72.5928583	-47.5767609	4.5787072
H	79.1417649	-35.6349870	4.5787072
H	83.7419386	-22.8157616	4.5787072
H	86.2801078	9.4347363	-4.5787072
H	86.6937744	-4.1786035	-4.5787072
H	84.9727526	-17.6890521	-4.5787072
H	81.1594196	-30.7639377	-4.5787072
H	75.3476724	-43.0813129	-4.5787072
H	67.6806153	-54.3378833	-4.5787072
H	58.3470369	-64.2564746	-4.5787072
H	47.5767609	-72.5928583	-4.5787072
H	35.6349870	-79.1417649	-4.5787072
H	22.8157616	-83.7419386	-4.5787072
H	9.4347363	-86.2801078	-4.5787072
H	-4.1786035	-86.6937744	-4.5787072
H	-17.6890521	-84.9727526	-4.5787072
H	-30.7639377	-81.1594196	-4.5787072
H	-43.0813129	-75.3476724	-4.5787072
H	-54.3378833	-67.6806153	-4.5787072
H	-64.2564746	-58.3470369	-4.5787072
H	-72.5928583	-47.5767609	-4.5787072
H	-79.1417649	-35.6349870	-4.5787072
H	-83.7419386	-22.8157616	-4.5787072
H	-86.2801078	-9.4347363	-4.5787072
H	-86.6937744	4.1786035	-4.5787072
H	-84.9727526	17.6890521	-4.5787072
H	-81.1594196	30.7639377	-4.5787072
H	-75.3476724	43.0813129	-4.5787072
H	-67.6806153	54.3378833	-4.5787072
H	-58.3470369	64.2564746	-4.5787072
H	-47.5767609	72.5928583	-4.5787072
H	-35.6349870	79.1417649	-4.5787072
H	-22.8157616	83.7419386	-4.5787072
H	-9.4347363	86.2801078	-4.5787072
H	4.1786035	86.6937744	-4.5787072

H	17.6890521	84.9727526	-4.5787072
H	30.7639377	81.1594196	-4.5787072
H	43.0813129	75.3476724	-4.5787072
H	54.3378833	67.6806153	-4.5787072
H	64.2564746	58.3470369	-4.5787072
H	72.5928583	47.5767609	-4.5787072
H	79.1417649	35.6349870	-4.5787072
H	83.7419386	22.8157616	-4.5787072
H	86.2801078	-9.4347363	-4.5787072
H	86.6937744	4.1786035	-4.5787072
H	84.9727526	17.6890521	-4.5787072
H	81.1594196	30.7639377	-4.5787072
H	75.3476724	43.0813129	-4.5787072
H	67.6806153	54.3378833	-4.5787072
H	58.3470369	64.2564746	-4.5787072
H	47.5767609	72.5928583	-4.5787072
H	35.6349870	79.1417649	-4.5787072
H	22.8157616	83.7419386	-4.5787072
H	9.4347363	86.2801078	-4.5787072
H	-4.1786035	86.6937744	-4.5787072
H	-17.6890521	84.9727526	-4.5787072
H	-30.7639377	81.1594196	-4.5787072
H	-43.0813129	75.3476724	-4.5787072
H	-54.3378833	67.6806153	-4.5787072
H	-64.2564746	58.3470369	-4.5787072
H	-72.5928583	47.5767609	-4.5787072
H	-79.1417649	35.6349870	-4.5787072
H	-83.7419386	22.8157616	-4.5787072
H	-86.2801078	9.4347363	-4.5787072
H	-86.6937744	-4.1786035	-4.5787072
H	-84.9727526	-17.6890521	-4.5787072
H	-81.1594196	-30.7639377	-4.5787072
H	-75.3476724	-43.0813129	-4.5787072
H	-67.6806153	-54.3378833	-4.5787072
H	-58.3470369	-64.2564746	-4.5787072
H	-47.5767609	-72.5928583	-4.5787072
H	-35.6349870	-79.1417649	-4.5787072
H	-22.8157616	-83.7419386	-4.5787072
H	-9.4347363	-86.2801078	-4.5787072
H	4.1786035	-86.6937744	-4.5787072
H	17.6890521	-84.9727526	-4.5787072
H	30.7639377	-81.1594196	-4.5787072
H	43.0813129	-75.3476724	-4.5787072
H	54.3378833	-67.6806153	-4.5787072
H	64.2564746	-58.3470369	-4.5787072
H	72.5928583	-47.5767609	-4.5787072
H	79.1417649	-35.6349870	-4.5787072
H	83.7419386	-22.8157616	-4.5787072
H	86.2801078	9.4347363	4.5787072

H	84.9727526	-17.6890521	4.5787072
H	81.1594196	-30.7639377	4.5787072
H	75.3476724	-43.0813129	4.5787072
H	67.6806153	-54.3378833	4.5787072
H	58.3470369	-64.2564746	4.5787072
H	47.5767609	-72.5928583	4.5787072
H	35.6349870	-79.1417649	4.5787072
H	22.8157616	-83.7419386	4.5787072
H	9.4347363	-86.2801078	4.5787072
H	-4.1786035	-86.6937744	4.5787072
H	-17.6890521	-84.9727526	4.5787072
H	-30.7639377	-81.1594196	4.5787072
H	-43.0813129	-75.3476724	4.5787072
H	-54.3378833	-67.6806153	4.5787072
H	-64.2564746	-58.3470369	4.5787072
H	-72.5928583	-47.5767609	4.5787072
H	-79.1417649	-35.6349870	4.5787072
H	-83.7419386	-22.8157616	4.5787072
H	-86.2801078	-9.4347363	4.5787072
H	-86.6937744	4.1786035	4.5787072
H	-84.9727526	17.6890521	4.5787072
H	-81.1594196	30.7639377	4.5787072
H	-75.3476724	43.0813129	4.5787072
H	-67.6806153	54.3378833	4.5787072
H	-58.3470369	64.2564746	4.5787072
H	-47.5767609	72.5928583	4.5787072
H	-35.6349870	79.1417649	4.5787072
H	-22.8157616	83.7419386	4.5787072
H	-9.4347363	86.2801078	4.5787072
H	4.1786035	86.6937744	4.5787072
H	17.6890521	84.9727526	4.5787072
H	30.7639377	81.1594196	4.5787072
H	43.0813129	75.3476724	4.5787072
H	54.3378833	67.6806153	4.5787072
H	64.2564746	58.3470369	4.5787072
H	72.5928583	47.5767609	4.5787072
H	79.1417649	35.6349870	4.5787072
H	83.7419386	22.8157616	4.5787072
C	85.9738821	11.6846152	-0.0000000
C	83.0875245	24.9900364	-0.0000000
C	78.1552762	37.6801200	0.0000000
C	71.2985856	49.4423941	0.0000000
C	62.6862872	59.9872322	0.0000000
C	52.5304444	69.0549857	0.0000000
C	41.0811278	76.4223762	-0.0000000
C	28.6202574	81.9079942	-0.0000000
C	15.4546613	85.3767655	0.0000000
C	1.9085201	86.7432775	-0.0000000
C	-11.6846152	85.9738821	-0.0000000

C	-24.9900364	83.0875245	-0.0000000
C	-37.6801200	78.1552762	-0.0000000
C	-49.4423941	71.2985856	0.0000000
C	-59.9872322	62.6862872	0.0000000
C	-69.0549857	52.5304444	-0.0000000
C	-76.4223762	41.0811278	0.0000000
C	-81.9079942	28.6202574	0.0000000
C	-85.3767655	15.4546613	0.0000000
C	-86.7432775	1.9085201	-0.0000000
C	-85.9738821	-11.6846152	-0.0000000
C	-83.0875245	-24.9900364	0.0000000
C	-78.1552762	-37.6801200	-0.0000000
C	-71.2985856	-49.4423941	0.0000000
C	-62.6862872	-59.9872322	0.0000000
C	-52.5304444	-69.0549857	-0.0000000
C	-41.0811278	-76.4223762	-0.0000000
C	-28.6202574	-81.9079942	0.0000000
C	-15.4546613	-85.3767655	-0.0000000
C	-1.9085201	-86.7432775	0.0000000
C	11.6846152	-85.9738821	0.0000000
C	24.9900364	-83.0875245	0.0000000
C	37.6801200	-78.1552762	-0.0000000
C	49.4423941	-71.2985856	-0.0000000
C	59.9872322	-62.6862872	-0.0000000
C	69.0549857	-52.5304444	-0.0000000
C	76.4223762	-41.0811278	0.0000000
C	81.9079942	-28.6202574	0.0000000
C	85.3767655	-15.4546613	-0.0000000
C	85.9738821	-11.6846152	0.0000000
C	83.0875245	-24.9900364	-0.0000000
C	78.1552762	-37.6801200	0.0000000
C	71.2985856	-49.4423941	0.0000000
C	62.6862872	-59.9872322	-0.0000000
C	52.5304444	-69.0549857	0.0000000
C	41.0811278	-76.4223762	-0.0000000
C	28.6202574	-81.9079942	-0.0000000
C	15.4546613	-85.3767655	0.0000000
C	1.9085201	-86.7432775	0.0000000
C	-11.6846152	-85.9738821	-0.0000000
C	-24.9900364	-83.0875245	0.0000000
C	-37.6801200	-78.1552762	-0.0000000
C	-49.4423941	-71.2985856	0.0000000
C	-59.9872322	-62.6862872	-0.0000000
C	-69.0549857	-52.5304444	0.0000000
C	-76.4223762	-41.0811278	-0.0000000
C	-81.9079942	-28.6202574	-0.0000000
C	-85.3767655	-15.4546613	-0.0000000
C	-86.7432775	-1.9085201	-0.0000000
C	-85.9738821	11.6846152	-0.0000000

C	-83.0875245	24.9900364	-0.0000000
C	-78.1552762	37.6801200	-0.0000000
C	-71.2985856	49.4423941	0.0000000
C	-62.6862872	59.9872322	-0.0000000
C	-52.5304444	69.0549857	0.0000000
C	-41.0811278	76.4223762	-0.0000000
C	-28.6202574	81.9079942	-0.0000000
C	-15.4546613	85.3767655	0.0000000
C	-1.9085201	86.7432775	0.0000000
C	11.6846152	85.9738821	-0.0000000
C	24.9900364	83.0875245	0.0000000
C	37.6801200	78.1552762	0.0000000
C	49.4423941	71.2985856	0.0000000
C	59.9872322	62.6862872	-0.0000000
C	69.0549857	52.5304444	-0.0000000
C	76.4223762	41.0811278	0.0000000
C	81.9079942	28.6202574	-0.0000000
C	85.3767655	15.4546613	0.0000000
C	85.8004735	12.9013824	-0.0000000
C	82.7259065	26.1646962	0.0000000
C	77.6143530	38.7837483	-0.0000000
C	70.5916766	50.4478158	0.0000000
C	61.8307989	60.8696906	-0.0000000
C	51.5474417	69.7927517	-0.0000000
C	39.9948153	76.9972836	0.0000000
C	27.4573839	82.3058868	0.0000000
C	14.2438606	85.5878459	-0.0000000
C	0.6796062	86.7623482	0.0000000
C	-12.9013824	85.8004735	0.0000000
C	-26.1646962	82.7259065	0.0000000
C	-38.7837483	77.6143530	-0.0000000
C	-50.4478158	70.5916766	0.0000000
C	-60.8696906	61.8307989	-0.0000000
C	-69.7927517	51.5474417	-0.0000000
C	-76.9972836	39.9948153	0.0000000
C	-82.3058868	27.4573839	0.0000000
C	-85.5878459	14.2438606	0.0000000
C	-86.7623482	0.6796062	0.0000000
C	-85.8004735	-12.9013824	-0.0000000
C	-82.7259065	-26.1646962	0.0000000
C	-77.6143530	-38.7837483	0.0000000
C	-70.5916766	-50.4478158	0.0000000
C	-61.8307989	-60.8696906	0.0000000
C	-51.5474417	-69.7927517	0.0000000
C	-39.9948153	-76.9972836	0.0000000
C	-27.4573839	-82.3058868	-0.0000000
C	-14.2438606	-85.5878459	-0.0000000
C	-0.6796062	-86.7623482	0.0000000
C	12.9013824	-85.8004735	0.0000000

C	26.1646962	-82.7259065	0.0000000
C	38.7837483	-77.6143530	-0.0000000
C	50.4478158	-70.5916766	0.0000000
C	60.8696906	-61.8307989	-0.0000000
C	69.7927517	-51.5474417	-0.0000000
C	76.9972836	-39.9948153	-0.0000000
C	82.3058868	-27.4573839	-0.0000000
C	85.5878459	-14.2438606	-0.0000000
C	85.8004735	-12.9013824	-0.0000000
C	82.7259065	-26.1646962	0.0000000
C	77.6143530	-38.7837483	0.0000000
C	70.5916766	-50.4478158	-0.0000000
C	61.8307989	-60.8696906	-0.0000000
C	51.5474417	-69.7927517	-0.0000000
C	39.9948153	-76.9972836	0.0000000
C	27.4573839	-82.3058868	0.0000000
C	14.2438606	-85.5878459	0.0000000
C	0.6796062	-86.7623482	0.0000000
C	-12.9013824	-85.8004735	-0.0000000
C	-26.1646962	-82.7259065	0.0000000
C	-38.7837483	-77.6143530	-0.0000000
C	-50.4478158	-70.5916766	-0.0000000
C	-60.8696906	-61.8307989	-0.0000000
C	-69.7927517	-51.5474417	-0.0000000
C	-76.9972836	-39.9948153	-0.0000000
C	-82.3058868	-27.4573839	0.0000000
C	-85.5878459	-14.2438606	0.0000000
C	-86.7623482	-0.6796062	0.0000000
C	-85.8004735	12.9013824	-0.0000000
C	-82.7259065	26.1646962	-0.0000000
C	-77.6143530	38.7837483	0.0000000
C	-70.5916766	50.4478158	0.0000000
C	-61.8307989	60.8696906	0.0000000
C	-51.5474417	69.7927517	0.0000000
C	-39.9948153	76.9972836	-0.0000000
C	-27.4573839	82.3058868	0.0000000
C	-14.2438606	85.5878459	0.0000000
C	-0.6796062	86.7623482	0.0000000
C	12.9013824	85.8004735	0.0000000
C	26.1646962	82.7259065	0.0000000
C	38.7837483	77.6143530	0.0000000
C	50.4478158	70.5916766	0.0000000
C	60.8696906	61.8307989	-0.0000000
C	69.7927517	51.5474417	-0.0000000
C	76.9972836	39.9948153	-0.0000000
C	82.3058868	27.4573839	0.0000000
C	85.5878459	14.2438606	-0.0000000

Horizontal P_{40}

```
1560
Energy =
Zn   86.2751806   -6.7900040    0.0000000
C    89.9587609   -4.5529546    0.0000000
C    89.0812442   -3.5037871    0.0000000
C    87.7509444   -4.0740047    0.0000000
C    89.1657038   -5.7620358    0.0000000
N    87.8295925   -5.4376306    0.0000000
C    89.6847703   -7.0583445    0.0000000
H    90.7737123   -7.1440461    0.0000000
C    88.9693070   -8.2574936    0.0000000
C    89.5634583   -9.5757505    0.0000000
C    88.5326194  -10.4747271    0.0000000
C    87.3078994   -9.7034251    0.0000000
N    87.5988973   -8.3688910    0.0000000
C    86.0021351  -10.2613131    0.0000000
C    84.7988046   -9.5048507    0.0000000
C    83.4680383  -10.0747609    0.0000000
C    82.5907128   -9.0256832    0.0000000
C    83.3838092   -7.8172832    0.0000000
N    84.7201068   -8.1415393    0.0000000
C    82.8644880   -6.5215766    0.0000000
H    81.7755513   -6.4358755    0.0000000
C    83.5801087   -5.3230621    0.0000000
C    82.9858120   -4.0054719    0.0000000
C    84.0164480   -3.1065540    0.0000000
C    85.2416768   -3.8776254    0.0000000
N    84.9506790   -5.2118412    0.0000000
H    91.0473109   -4.5187992    0.0000000
H    89.3005485   -2.4385632    0.0000000
H    90.6332634   -9.7797721    0.0000000
H    88.5825859  -11.5611431    0.0000000
H    83.2475447  -11.1393570    0.0000000
H    81.5021668   -9.0596764    0.0000000
H    81.9159855   -3.8016111    0.0000000
H    83.9652087   -2.0205721    0.0000000
C    86.5485292   -3.3187187    0.0000000
C    86.6479110   -1.9067519    0.0000000
C    86.6877662   -0.6789585    0.0000000
C    86.6877662    0.6789585    0.0000000
C    86.6479110    1.9067519    0.0000000
Zn   86.2751806    6.7900040    0.0000000
Zn   84.1507993   20.2028195    0.0000000
Zn   79.9543461   33.1181745    0.0000000
Zn   73.7891515   45.2180502    0.0000000
Zn   65.8070231   56.2045074    0.0000000
Zn   56.2045074   65.8070231    0.0000000
Zn   45.2180502   73.7891515    0.0000000
```

Zn	33.1181745	79.9543461	0.0000000
Zn	20.2028195	84.1507993	0.0000000
Zn	6.7900040	86.2751806	0.0000000
Zn	-6.7900040	86.2751806	0.0000000
Zn	-20.2028195	84.1507993	0.0000000
Zn	-33.1181745	79.9543461	0.0000000
Zn	-45.2180502	73.7891515	0.0000000
Zn	-56.2045074	65.8070231	0.0000000
Zn	-65.8070231	56.2045074	0.0000000
Zn	-73.7891515	45.2180502	0.0000000
Zn	-79.9543461	33.1181745	0.0000000
Zn	-84.1507993	20.2028195	0.0000000
Zn	-86.2751806	6.7900040	0.0000000
Zn	-86.2751806	-6.7900040	0.0000000
Zn	-84.1507993	-20.2028195	0.0000000
Zn	-79.9543461	-33.1181745	0.0000000
Zn	-73.7891515	-45.2180502	0.0000000
Zn	-65.8070231	-56.2045074	0.0000000
Zn	-56.2045074	-65.8070231	0.0000000
Zn	-45.2180502	-73.7891515	0.0000000
Zn	-33.1181745	-79.9543461	0.0000000
Zn	-20.2028195	-84.1507993	0.0000000
Zn	-6.7900040	-86.2751806	0.0000000
Zn	6.7900040	-86.2751806	0.0000000
Zn	20.2028195	-84.1507993	0.0000000
Zn	33.1181745	-79.9543461	0.0000000
Zn	45.2180502	-73.7891515	0.0000000
Zn	56.2045074	-65.8070231	0.0000000
Zn	65.8070231	-56.2045074	0.0000000
Zn	73.7891515	-45.2180502	0.0000000
Zn	79.9543461	-33.1181745	0.0000000
Zn	84.1507993	-20.2028195	0.0000000
C	89.5634583	9.5757505	0.0000000
C	86.9628061	23.4686688	0.0000000
C	82.2208410	36.7837106	0.0000000
C	75.4543260	49.1930153	0.0000000
C	66.8298750	60.3910248	0.0000000
C	56.5598506	70.1020068	0.0000000
C	44.8971351	78.0868448	0.0000000
C	32.1289031	84.1489254	0.0000000
C	18.5695508	88.1389803	0.0000000
C	4.5529546	89.9587609	0.0000000
C	-9.5757505	89.5634583	0.0000000
C	-23.4686688	86.9628061	0.0000000
C	-36.7837106	82.2208410	0.0000000
C	-49.1930153	75.4543260	0.0000000
C	-60.3910248	66.8298750	0.0000000
C	-70.1020068	56.5598506	0.0000000
C	-78.0868448	44.8971351	0.0000000

C	-84.1489254	32.1289031	0.0000000
C	-88.1389803	18.5695508	0.0000000
C	-89.9587609	4.5529546	0.0000000
C	-89.5634583	-9.5757505	0.0000000
C	-86.9628061	-23.4686688	0.0000000
C	-82.2208410	-36.7837106	0.0000000
C	-75.4543260	-49.1930153	0.0000000
C	-66.8298750	-60.3910248	0.0000000
C	-56.5598506	-70.1020068	0.0000000
C	-44.8971351	-78.0868448	0.0000000
C	-32.1289031	-84.1489254	0.0000000
C	-18.5695508	-88.1389803	0.0000000
C	-4.5529546	-89.9587609	0.0000000
C	9.5757505	-89.5634583	0.0000000
C	23.4686688	-86.9628061	0.0000000
C	36.7837106	-82.2208410	0.0000000
C	49.1930153	-75.4543260	0.0000000
C	60.3910248	-66.8298750	0.0000000
C	70.1020068	-56.5598506	0.0000000
C	78.0868448	-44.8971351	0.0000000
C	84.1489254	-32.1289031	0.0000000
C	88.1389803	-18.5695508	0.0000000
C	88.5326194	10.4747271	0.0000000
C	85.8040276	24.1953188	0.0000000
C	80.9626558	37.3201414	0.0000000
C	74.1277148	49.5260183	0.0000000
C	65.4675035	60.5124003	0.0000000
C	55.1952649	70.0087661	0.0000000
C	43.5639357	77.7812838	0.0000000
C	30.8599179	83.6385681	0.0000000
C	17.3960264	87.4363932	0.0000000
C	3.5037871	89.0812442	0.0000000
C	-10.4747271	88.5326194	0.0000000
C	-24.1953188	85.8040276	0.0000000
C	-37.3201414	80.9626558	0.0000000
C	-49.5260183	74.1277148	0.0000000
C	-60.5124003	65.4675035	0.0000000
C	-70.0087661	55.1952649	0.0000000
C	-77.7812838	43.5639357	0.0000000
C	-83.6385681	30.8599179	0.0000000
C	-87.4363932	17.3960264	0.0000000
C	-89.0812442	3.5037871	0.0000000
C	-88.5326194	-10.4747271	0.0000000
C	-85.8040276	-24.1953188	0.0000000
C	-80.9626558	-37.3201414	0.0000000
C	-74.1277148	-49.5260183	0.0000000
C	-65.4675035	-60.5124003	0.0000000
C	-55.1952649	-70.0087661	0.0000000
C	-43.5639357	-77.7812838	0.0000000

C	-30.8599179	-83.6385681	0.0000000
C	-17.3960264	-87.4363932	0.0000000
C	-3.5037871	-89.0812442	0.0000000
C	10.4747271	-88.5326194	0.0000000
C	24.1953188	-85.8040276	0.0000000
C	37.3201414	-80.9626558	0.0000000
C	49.5260183	-74.1277148	0.0000000
C	60.5124003	-65.4675035	0.0000000
C	70.0087661	-55.1952649	0.0000000
C	77.7812838	-43.5639357	0.0000000
C	83.6385681	-30.8599179	0.0000000
C	87.4363932	-17.3960264	0.0000000
C	87.3078994	9.7034251	0.0000000
C	84.7150442	23.2419244	0.0000000
C	80.0362234	36.2081303	0.0000000
C	73.3866452	48.2827720	0.0000000
C	64.9300442	59.1685315	0.0000000
C	54.8746500	68.5973654	0.0000000
C	43.4680599	76.3371046	0.0000000
C	30.9911418	82.1971708	0.0000000
C	17.7511190	86.0332699	0.0000000
C	4.0740047	87.7509444	0.0000000
C	-9.7034251	87.3078994	0.0000000
C	-23.2419244	84.7150442	0.0000000
C	-36.2081303	80.0362234	0.0000000
C	-48.2827720	73.3866452	0.0000000
C	-59.1685315	64.9300442	0.0000000
C	-68.5973654	54.8746500	0.0000000
C	-76.3371046	43.4680599	0.0000000
C	-82.1971708	30.9911418	0.0000000
C	-86.0332699	17.7511190	0.0000000
C	-87.7509444	4.0740047	0.0000000
C	-87.3078994	-9.7034251	0.0000000
C	-84.7150442	-23.2419244	0.0000000
C	-80.0362234	-36.2081303	0.0000000
C	-73.3866452	-48.2827720	0.0000000
C	-64.9300442	-59.1685315	0.0000000
C	-54.8746500	-68.5973654	0.0000000
C	-43.4680599	-76.3371046	0.0000000
C	-30.9911418	-82.1971708	0.0000000
C	-17.7511190	-86.0332699	0.0000000
C	-4.0740047	-87.7509444	0.0000000
C	9.7034251	-87.3078994	0.0000000
C	23.2419244	-84.7150442	0.0000000
C	36.2081303	-80.0362234	0.0000000
C	48.2827720	-73.3866452	0.0000000
C	59.1685315	-64.9300442	0.0000000
C	68.5973654	-54.8746500	0.0000000
C	76.3371046	-43.4680599	0.0000000

C	82.1971708	-30.9911418	0.0000000
C	86.0332699	-17.7511190	0.0000000
C	88.9693070	8.2574936	0.0000000
C	86.5821906	22.0736961	0.0000000
C	82.0631334	35.3463710	0.0000000
C	75.5234094	47.7487008	0.0000000
C	67.1240484	58.9752992	0.0000000
C	57.0718706	68.7497300	0.0000000
C	45.6143939	76.8313143	0.0000000
C	33.0337395	83.0210567	0.0000000
C	19.6396848	87.1665451	0.0000000
C	5.7620358	89.1657038	0.0000000
C	-8.2574936	88.9693070	0.0000000
C	-22.0736961	86.5821906	0.0000000
C	-35.3463710	82.0631334	0.0000000
C	-47.7487008	75.5234094	0.0000000
C	-58.9752992	67.1240484	0.0000000
C	-68.7497300	57.0718706	0.0000000
C	-76.8313143	45.6143939	0.0000000
C	-83.0210567	33.0337395	0.0000000
C	-87.1665451	19.6396848	0.0000000
C	-89.1657038	5.7620358	0.0000000
C	-88.9693070	-8.2574936	0.0000000
C	-86.5821906	-22.0736961	0.0000000
C	-82.0631334	-35.3463710	0.0000000
C	-75.5234094	-47.7487008	0.0000000
C	-67.1240484	-58.9752992	0.0000000
C	-57.0718706	-68.7497300	0.0000000
C	-45.6143939	-76.8313143	0.0000000
C	-33.0337395	-83.0210567	0.0000000
C	-19.6396848	-87.1665451	0.0000000
C	-5.7620358	-89.1657038	0.0000000
C	8.2574936	-88.9693070	0.0000000
C	22.0736961	-86.5821906	0.0000000
C	35.3463710	-82.0631334	0.0000000
C	47.7487008	-75.5234094	0.0000000
C	58.9752992	-67.1240484	0.0000000
C	68.7497300	-57.0718706	0.0000000
C	76.8313143	-45.6143939	0.0000000
C	83.0210567	-33.0337395	0.0000000
C	87.1665451	-19.6396848	0.0000000
N	87.5988973	8.3688910	0.0000000
N	85.2112265	21.9693427	0.0000000
N	80.7253725	35.0288362	0.0000000
N	74.2517920	47.2258036	0.0000000
N	65.9498859	58.2599149	0.0000000
N	56.0240747	67.8594738	0.0000000
N	44.7187649	75.7881073	0.0000000
N	32.3123307	81.8505860	0.0000000

N	19.1102597	85.8976316	0.0000000
N	5.4376306	87.8295925	0.0000000
N	-8.3688910	87.5988973	0.0000000
N	-21.9693427	85.2112265	0.0000000
N	-35.0288362	80.7253725	0.0000000
N	-47.2258036	74.2517920	0.0000000
N	-58.2599149	65.9498859	0.0000000
N	-67.8594738	56.0240747	0.0000000
N	-75.7881073	44.7187649	0.0000000
N	-81.8505860	32.3123307	0.0000000
N	-85.8976316	19.1102597	0.0000000
N	-87.8295925	5.4376306	0.0000000
N	-87.5988973	-8.3688910	0.0000000
N	-85.2112265	-21.9693427	0.0000000
N	-80.7253725	-35.0288362	0.0000000
N	-74.2517920	-47.2258036	0.0000000
N	-65.9498859	-58.2599149	0.0000000
N	-56.0240747	-67.8594738	0.0000000
N	-44.7187649	-75.7881073	0.0000000
N	-32.3123307	-81.8505860	0.0000000
N	-19.1102597	-85.8976316	0.0000000
N	-5.4376306	-87.8295925	0.0000000
N	8.3688910	-87.5988973	0.0000000
N	21.9693427	-85.2112265	0.0000000
N	35.0288362	-80.7253725	0.0000000
N	47.2258036	-74.2517920	0.0000000
N	58.2599149	-65.9498859	0.0000000
N	67.8594738	-56.0240747	0.0000000
N	75.7881073	-44.7187649	0.0000000
N	81.8505860	-32.3123307	0.0000000
N	85.8976316	-19.1102597	0.0000000
C	89.6847703	7.0583445	0.0000000
C	87.4764336	21.0012336	0.0000000
C	83.1141368	34.4270027	0.0000000
C	76.7052941	47.0050647	0.0000000
C	68.4077125	58.4257060	0.0000000
C	58.4257060	68.4077125	0.0000000
C	47.0050647	76.7052941	0.0000000
C	34.4270027	83.1141368	0.0000000
C	21.0012336	87.4764336	0.0000000
C	7.0583445	89.6847703	0.0000000
C	-7.0583445	89.6847703	0.0000000
C	-21.0012336	87.4764336	0.0000000
C	-34.4270027	83.1141368	0.0000000
C	-47.0050647	76.7052941	0.0000000
C	-58.4257060	68.4077125	0.0000000
C	-68.4077125	58.4257060	0.0000000
C	-76.7052941	47.0050647	0.0000000
C	-83.1141368	34.4270027	0.0000000

C	-87.4764336	21.0012336	0.0000000
C	-89.6847703	7.0583445	0.0000000
C	-89.6847703	-7.0583445	0.0000000
C	-87.4764336	-21.0012336	0.0000000
C	-83.1141368	-34.4270027	0.0000000
C	-76.7052941	-47.0050647	0.0000000
C	-68.4077125	-58.4257060	0.0000000
C	-58.4257060	-68.4077125	0.0000000
C	-47.0050647	-76.7052941	0.0000000
C	-34.4270027	-83.1141368	0.0000000
C	-21.0012336	-87.4764336	0.0000000
C	-7.0583445	-89.6847703	0.0000000
C	7.0583445	-89.6847703	0.0000000
C	21.0012336	-87.4764336	0.0000000
C	34.4270027	-83.1141368	0.0000000
C	47.0050647	-76.7052941	0.0000000
C	58.4257060	-68.4077125	0.0000000
C	68.4077125	-58.4257060	0.0000000
C	76.7052941	-47.0050647	0.0000000
C	83.1141368	-34.4270027	0.0000000
C	87.4764336	-21.0012336	0.0000000
H	90.7737123	7.1440461	0.0000000
H	88.5385623	21.2562282	0.0000000
H	84.1232989	34.8450113	0.0000000
H	77.6366408	47.5757947	0.0000000
H	69.2383110	59.1351041	0.0000000
H	59.1351041	69.2383110	0.0000000
H	47.5757947	77.6366408	0.0000000
H	34.8450113	84.1232989	0.0000000
H	21.2562282	88.5385623	0.0000000
H	7.1440461	90.7737123	0.0000000
H	-7.1440461	90.7737123	0.0000000
H	-21.2562282	88.5385623	0.0000000
H	-34.8450113	84.1232989	0.0000000
H	-47.5757947	77.6366408	0.0000000
H	-59.1351041	69.2383110	0.0000000
H	-69.2383110	59.1351041	0.0000000
H	-77.6366408	47.5757947	0.0000000
H	-84.1232989	34.8450113	0.0000000
H	-88.5385623	21.2562282	0.0000000
H	-90.7737123	7.1440461	0.0000000
H	-90.7737123	-7.1440461	0.0000000
H	-88.5385623	-21.2562282	0.0000000
H	-84.1232989	-34.8450113	0.0000000
H	-77.6366408	-47.5757947	0.0000000
H	-69.2383110	-59.1351041	0.0000000
H	-59.1351041	-69.2383110	0.0000000
H	-47.5757947	-77.6366408	0.0000000
H	-34.8450113	-84.1232989	0.0000000

H	-21.2562282	-88.5385623	0.0000000
H	-7.1440461	-90.7737123	0.0000000
H	7.1440461	-90.7737123	0.0000000
H	21.2562282	-88.5385623	0.0000000
H	34.8450113	-84.1232989	0.0000000
H	47.5757947	-77.6366408	0.0000000
H	59.1351041	-69.2383110	0.0000000
H	69.2383110	-59.1351041	0.0000000
H	77.6366408	-47.5757947	0.0000000
H	84.1232989	-34.8450113	0.0000000
H	88.5385623	-21.2562282	0.0000000
C	89.1657038	5.7620358	0.0000000
C	87.1665451	19.6396848	0.0000000
C	83.0210567	33.0337395	0.0000000
C	76.8313143	45.6143939	0.0000000
C	68.7497300	57.0718706	0.0000000
C	58.9752992	67.1240484	0.0000000
C	47.7487008	75.5234094	0.0000000
C	35.3463710	82.0631334	0.0000000
C	22.0736961	86.5821906	0.0000000
C	8.2574936	88.9693070	0.0000000
C	-5.7620358	89.1657038	0.0000000
C	-19.6396848	87.1665451	0.0000000
C	-33.0337395	83.0210567	0.0000000
C	-45.6143939	76.8313143	0.0000000
C	-57.0718706	68.7497300	0.0000000
C	-67.1240484	58.9752992	0.0000000
C	-75.5234094	47.7487008	0.0000000
C	-82.0631334	35.3463710	0.0000000
C	-86.5821906	22.0736961	0.0000000
C	-88.9693070	8.2574936	0.0000000
C	-89.1657038	-5.7620358	0.0000000
C	-87.1665451	-19.6396848	0.0000000
C	-83.0210567	-33.0337395	0.0000000
C	-76.8313143	-45.6143939	0.0000000
C	-68.7497300	-57.0718706	0.0000000
C	-58.9752992	-67.1240484	0.0000000
C	-47.7487008	-75.5234094	0.0000000
C	-35.3463710	-82.0631334	0.0000000
C	-22.0736961	-86.5821906	0.0000000
C	-8.2574936	-88.9693070	0.0000000
C	5.7620358	-89.1657038	0.0000000
C	19.6396848	-87.1665451	0.0000000
C	33.0337395	-83.0210567	0.0000000
C	45.6143939	-76.8313143	0.0000000
C	57.0718706	-68.7497300	0.0000000
C	67.1240484	-58.9752992	0.0000000
C	75.5234094	-47.7487008	0.0000000
C	82.0631334	-35.3463710	0.0000000

C	86.5821906	-22.0736961	0.0000000
C	89.9587609	4.5529546	0.0000000
C	88.1389803	18.5695508	0.0000000
C	84.1489254	32.1289031	0.0000000
C	78.0868448	44.8971351	0.0000000
C	70.1020068	56.5598506	0.0000000
C	60.3910248	66.8298750	0.0000000
C	49.1930153	75.4543260	0.0000000
C	36.7837106	82.2208410	0.0000000
C	23.4686688	86.9628061	0.0000000
C	9.5757505	89.5634583	0.0000000
C	-4.5529546	89.9587609	0.0000000
C	-18.5695508	88.1389803	0.0000000
C	-32.1289031	84.1489254	0.0000000
C	-44.8971351	78.0868448	0.0000000
C	-56.5598506	70.1020068	0.0000000
C	-66.8298750	60.3910248	0.0000000
C	-75.4543260	49.1930153	0.0000000
C	-82.2208410	36.7837106	0.0000000
C	-86.9628061	23.4686688	0.0000000
C	-89.5634583	9.5757505	0.0000000
C	-89.9587609	-4.5529546	0.0000000
C	-88.1389803	-18.5695508	0.0000000
C	-84.1489254	-32.1289031	0.0000000
C	-78.0868448	-44.8971351	0.0000000
C	-70.1020068	-56.5598506	0.0000000
C	-60.3910248	-66.8298750	0.0000000
C	-49.1930153	-75.4543260	0.0000000
C	-36.7837106	-82.2208410	0.0000000
C	-23.4686688	-86.9628061	0.0000000
C	-9.5757505	-89.5634583	0.0000000
C	4.5529546	-89.9587609	0.0000000
C	18.5695508	-88.1389803	0.0000000
C	32.1289031	-84.1489254	0.0000000
C	44.8971351	-78.0868448	0.0000000
C	56.5598506	-70.1020068	0.0000000
C	66.8298750	-60.3910248	0.0000000
C	75.4543260	-49.1930153	0.0000000
C	82.2208410	-36.7837106	0.0000000
C	86.9628061	-23.4686688	0.0000000
C	89.0812442	3.5037871	0.0000000
C	87.4363932	17.3960264	0.0000000
C	83.6385681	30.8599179	0.0000000
C	77.7812838	43.5639357	0.0000000
C	70.0087661	55.1952649	0.0000000
C	60.5124003	65.4675035	0.0000000
C	49.5260183	74.1277148	0.0000000
C	37.3201414	80.9626558	0.0000000
C	24.1953188	85.8040276	0.0000000

C	10.4747271	88.5326194	0.0000000
C	-3.5037871	89.0812442	0.0000000
C	-17.3960264	87.4363932	0.0000000
C	-30.8599179	83.6385681	0.0000000
C	-43.5639357	77.7812838	0.0000000
C	-55.1952649	70.0087661	0.0000000
C	-65.4675035	60.5124003	0.0000000
C	-74.1277148	49.5260183	0.0000000
C	-80.9626558	37.3201414	0.0000000
C	-85.8040276	24.1953188	0.0000000
C	-88.5326194	10.4747271	0.0000000
C	-89.0812442	-3.5037871	0.0000000
C	-87.4363932	-17.3960264	0.0000000
C	-83.6385681	-30.8599179	0.0000000
C	-77.7812838	-43.5639357	0.0000000
C	-70.0087661	-55.1952649	0.0000000
C	-60.5124003	-65.4675035	0.0000000
C	-49.5260183	-74.1277148	0.0000000
C	-37.3201414	-80.9626558	0.0000000
C	-24.1953188	-85.8040276	0.0000000
C	-10.4747271	-88.5326194	0.0000000
C	3.5037871	-89.0812442	0.0000000
C	17.3960264	-87.4363932	0.0000000
C	30.8599179	-83.6385681	0.0000000
C	43.5639357	-77.7812838	0.0000000
C	55.1952649	-70.0087661	0.0000000
C	65.4675035	-60.5124003	0.0000000
C	74.1277148	-49.5260183	0.0000000
C	80.9626558	-37.3201414	0.0000000
C	85.8040276	-24.1953188	0.0000000
C	87.7509444	4.0740047	0.0000000
C	86.0332699	17.7511190	0.0000000
C	82.1971708	30.9911418	0.0000000
C	76.3371046	43.4680599	0.0000000
C	68.5973654	54.8746500	0.0000000
C	59.1685315	64.9300442	0.0000000
C	48.2827720	73.3866452	0.0000000
C	36.2081303	80.0362234	0.0000000
C	23.2419244	84.7150442	0.0000000
C	9.7034251	87.3078994	0.0000000
C	-4.0740047	87.7509444	0.0000000
C	-17.7511190	86.0332699	0.0000000
C	-30.9911418	82.1971708	0.0000000
C	-43.4680599	76.3371046	0.0000000
C	-54.8746500	68.5973654	0.0000000
C	-64.9300442	59.1685315	0.0000000
C	-73.3866452	48.2827720	0.0000000
C	-80.0362234	36.2081303	0.0000000
C	-84.7150442	23.2419244	0.0000000

C	-87.3078994	9.7034251	0.0000000
C	-87.7509444	-4.0740047	0.0000000
C	-86.0332699	-17.7511190	0.0000000
C	-82.1971708	-30.9911418	0.0000000
C	-76.3371046	-43.4680599	0.0000000
C	-68.5973654	-54.8746500	0.0000000
C	-59.1685315	-64.9300442	0.0000000
C	-48.2827720	-73.3866452	0.0000000
C	-36.2081303	-80.0362234	0.0000000
C	-23.2419244	-84.7150442	0.0000000
C	-9.7034251	-87.3078994	0.0000000
C	4.0740047	-87.7509444	0.0000000
C	17.7511190	-86.0332699	0.0000000
C	30.9911418	-82.1971708	0.0000000
C	43.4680599	-76.3371046	0.0000000
C	54.8746500	-68.5973654	0.0000000
C	64.9300442	-59.1685315	0.0000000
C	73.3866452	-48.2827720	0.0000000
C	80.0362234	-36.2081303	0.0000000
C	84.7150442	-23.2419244	0.0000000
N	87.8295925	5.4376306	0.0000000
N	85.8976316	19.1102597	0.0000000
N	81.8505860	32.3123307	0.0000000
N	75.7881073	44.7187649	0.0000000
N	67.8594738	56.0240747	0.0000000
N	58.2599149	65.9498859	0.0000000
N	47.2258036	74.2517920	0.0000000
N	35.0288362	80.7253725	0.0000000
N	21.9693427	85.2112265	0.0000000
N	8.3688910	87.5988973	0.0000000
N	-5.4376306	87.8295925	0.0000000
N	-19.1102597	85.8976316	0.0000000
N	-32.3123307	81.8505860	0.0000000
N	-44.7187649	75.7881073	0.0000000
N	-56.0240747	67.8594738	0.0000000
N	-65.9498859	58.2599149	0.0000000
N	-74.2517920	47.2258036	0.0000000
N	-80.7253725	35.0288362	0.0000000
N	-85.2112265	21.9693427	0.0000000
N	-87.5988973	8.3688910	0.0000000
N	-87.8295925	-5.4376306	0.0000000
N	-85.8976316	-19.1102597	0.0000000
N	-81.8505860	-32.3123307	0.0000000
N	-75.7881073	-44.7187649	0.0000000
N	-67.8594738	-56.0240747	0.0000000
N	-58.2599149	-65.9498859	0.0000000
N	-47.2258036	-74.2517920	0.0000000
N	-35.0288362	-80.7253725	0.0000000
N	-21.9693427	-85.2112265	0.0000000

N	-8.3688910	-87.5988973	0.0000000
N	5.4376306	-87.8295925	0.0000000
N	19.1102597	-85.8976316	0.0000000
N	32.3123307	-81.8505860	0.0000000
N	44.7187649	-75.7881073	0.0000000
N	56.0240747	-67.8594738	0.0000000
N	65.9498859	-58.2599149	0.0000000
N	74.2517920	-47.2258036	0.0000000
N	80.7253725	-35.0288362	0.0000000
N	85.2112265	-21.9693427	0.0000000
C	86.5485292	3.3187187	0.0000000
C	84.9638112	16.8170326	0.0000000
C	81.2870022	29.9012554	0.0000000
C	75.6086374	42.2492100	0.0000000
C	68.0685370	53.5568489	0.0000000
C	58.8523634	63.5457404	0.0000000
C	48.1870492	71.9699248	0.0000000
C	36.3352100	78.6219709	0.0000000
C	23.5886773	83.3380831	0.0000000
C	10.2613131	86.0021351	0.0000000
C	-3.3187187	86.5485292	0.0000000
C	-16.8170326	84.9638112	0.0000000
C	-29.9012554	81.2870022	0.0000000
C	-42.2492100	75.6086374	0.0000000
C	-53.5568489	68.0685370	0.0000000
C	-63.5457404	58.8523634	0.0000000
C	-71.9699248	48.1870492	0.0000000
C	-78.6219709	36.3352100	0.0000000
C	-83.3380831	23.5886773	0.0000000
C	-86.0021351	10.2613131	0.0000000
C	-86.5485292	-3.3187187	0.0000000
C	-84.9638112	-16.8170326	0.0000000
C	-81.2870022	-29.9012554	0.0000000
C	-75.6086374	-42.2492100	0.0000000
C	-68.0685370	-53.5568489	0.0000000
C	-58.8523634	-63.5457404	0.0000000
C	-48.1870492	-71.9699248	0.0000000
C	-36.3352100	-78.6219709	0.0000000
C	-23.5886773	-83.3380831	0.0000000
C	-10.2613131	-86.0021351	0.0000000
C	3.3187187	-86.5485292	0.0000000
C	16.8170326	-84.9638112	0.0000000
C	29.9012554	-81.2870022	0.0000000
C	42.2492100	-75.6086374	0.0000000
C	53.5568489	-68.0685370	0.0000000
C	63.5457404	-58.8523634	0.0000000
C	71.9699248	-48.1870492	0.0000000
C	78.6219709	-36.3352100	0.0000000
C	83.3380831	-23.5886773	0.0000000

C	85.2416768	3.8776254	0.0000000
C	83.5856161	17.1646215	0.0000000
C	79.8714000	30.0289676	0.0000000
C	74.1904851	42.1539009	0.0000000
C	66.6827542	53.2408653	0.0000000
C	57.5330725	63.0168629	0.0000000
C	46.9667357	71.2411762	0.0000000
C	35.2439220	77.7112952	0.0000000
C	22.6532859	82.2679043	0.0000000
C	9.5048507	84.7988046	0.0000000
C	-3.8776254	85.2416768	0.0000000
C	-17.1646215	83.5856161	0.0000000
C	-30.0289676	79.8714000	0.0000000
C	-42.1539009	74.1904851	0.0000000
C	-53.2408653	66.6827542	0.0000000
C	-63.0168629	57.5330725	0.0000000
C	-71.2411762	46.9667357	0.0000000
C	-77.7112952	35.2439220	0.0000000
C	-82.2679043	22.6532859	0.0000000
C	-84.7988046	9.5048507	0.0000000
C	-85.2416768	-3.8776254	0.0000000
C	-83.5856161	-17.1646215	0.0000000
C	-79.8714000	-30.0289676	0.0000000
C	-74.1904851	-42.1539009	0.0000000
C	-66.6827542	-53.2408653	0.0000000
C	-57.5330725	-63.0168629	0.0000000
C	-46.9667357	-71.2411762	0.0000000
C	-35.2439220	-77.7112952	0.0000000
C	-22.6532859	-82.2679043	0.0000000
C	-9.5048507	-84.7988046	0.0000000
C	3.8776254	-85.2416768	0.0000000
C	17.1646215	-83.5856161	0.0000000
C	30.0289676	-79.8714000	0.0000000
C	42.1539009	-74.1904851	0.0000000
C	53.2408653	-66.6827542	0.0000000
C	63.0168629	-57.5330725	0.0000000
C	71.2411762	-46.9667357	0.0000000
C	77.7112952	-35.2439220	0.0000000
C	82.2679043	-22.6532859	0.0000000
C	84.0164480	3.1065540	0.0000000
C	82.4960940	16.2113753	0.0000000
C	78.9444124	28.9170187	0.0000000
C	73.4488573	40.9106291	0.0000000
C	66.1447476	51.8968841	0.0000000
C	57.2119347	61.6052655	0.0000000
C	46.8703741	69.7967209	0.0000000
C	35.3747094	76.2695493	0.0000000
C	23.0080018	80.8643684	0.0000000
C	10.0747609	83.4680383	0.0000000

C	-3.1065540	84.0164480	0.0000000
C	-16.2113753	82.4960940	0.0000000
C	-28.9170187	78.9444124	0.0000000
C	-40.9106291	73.4488573	0.0000000
C	-51.8968841	66.1447476	0.0000000
C	-61.6052655	57.2119347	0.0000000
C	-69.7967209	46.8703741	0.0000000
C	-76.2695493	35.3747094	0.0000000
C	-80.8643684	23.0080018	0.0000000
C	-83.4680383	10.0747609	0.0000000
C	-84.0164480	-3.1065540	0.0000000
C	-82.4960940	-16.2113753	0.0000000
C	-78.9444124	-28.9170187	0.0000000
C	-73.4488573	-40.9106291	0.0000000
C	-66.1447476	-51.8968841	0.0000000
C	-57.2119347	-61.6052655	0.0000000
C	-46.8703741	-69.7967209	0.0000000
C	-35.3747094	-76.2695493	0.0000000
C	-23.0080018	-80.8643684	0.0000000
C	-10.0747609	-83.4680383	0.0000000
C	3.1065540	-84.0164480	0.0000000
C	16.2113753	-82.4960940	0.0000000
C	28.9170187	-78.9444124	0.0000000
C	40.9106291	-73.4488573	0.0000000
C	51.8968841	-66.1447476	0.0000000
C	61.6052655	-57.2119347	0.0000000
C	69.7967209	-46.8703741	0.0000000
C	76.2695493	-35.3747094	0.0000000
C	80.8643684	-23.0080018	0.0000000
C	82.9858120	4.0054719	0.0000000
C	81.3375251	16.9379990	0.0000000
C	77.6864384	29.4534563	0.0000000
C	72.1224538	41.2436719	0.0000000
C	64.7825749	52.0183313	0.0000000
C	55.8475341	61.5121268	0.0000000
C	45.5373417	69.4912895	0.0000000
C	34.1058687	75.7593461	0.0000000
C	21.8345961	80.1619562	0.0000000
C	9.0256832	82.5907128	0.0000000
C	-4.0054719	82.9858120	0.0000000
C	-16.9379990	81.3375251	0.0000000
C	-29.4534563	77.6864384	0.0000000
C	-41.2436719	72.1224538	0.0000000
C	-52.0183313	64.7825749	0.0000000
C	-61.5121268	55.8475341	0.0000000
C	-69.4912895	45.5373417	0.0000000
C	-75.7593461	34.1058687	0.0000000
C	-80.1619562	21.8345961	0.0000000
C	-82.5907128	9.0256832	0.0000000

C	-82.9858120	-4.0054719	0.0000000
C	-81.3375251	-16.9379990	0.0000000
C	-77.6864384	-29.4534563	0.0000000
C	-72.1224538	-41.2436719	0.0000000
C	-64.7825749	-52.0183313	0.0000000
C	-55.8475341	-61.5121268	0.0000000
C	-45.5373417	-69.4912895	0.0000000
C	-34.1058687	-75.7593461	0.0000000
C	-21.8345961	-80.1619562	0.0000000
C	-9.0256832	-82.5907128	0.0000000
C	4.0054719	-82.9858120	0.0000000
C	16.9379990	-81.3375251	0.0000000
C	29.4534563	-77.6864384	0.0000000
C	41.2436719	-72.1224538	0.0000000
C	52.0183313	-64.7825749	0.0000000
C	61.5121268	-55.8475341	0.0000000
C	69.4912895	-45.5373417	0.0000000
C	75.7593461	-34.1058687	0.0000000
C	80.1619562	-21.8345961	0.0000000
C	83.5801087	5.3230621	0.0000000
C	81.7183885	18.3323359	0.0000000
C	77.8444904	30.8902068	0.0000000
C	72.0538025	42.6874583	0.0000000
C	64.4889109	53.4336029	0.0000000
C	55.3360883	62.8640349	0.0000000
C	44.8207076	70.7465457	0.0000000
C	33.2016923	76.8870417	0.0000000
C	20.7651411	81.1343236	0.0000000
C	7.8172832	83.3838092	0.0000000
C	-5.3230621	83.5801087	0.0000000
C	-18.3323359	81.7183885	0.0000000
C	-30.8902068	77.8444904	0.0000000
C	-42.6874583	72.0538025	0.0000000
C	-53.4336029	64.4889109	0.0000000
C	-62.8640349	55.3360883	0.0000000
C	-70.7465457	44.8207076	0.0000000
C	-76.8870417	33.2016923	0.0000000
C	-81.1343236	20.7651411	0.0000000
C	-83.3838092	7.8172832	0.0000000
C	-83.5801087	-5.3230621	0.0000000
C	-81.7183885	-18.3323359	0.0000000
C	-77.8444904	-30.8902068	0.0000000
C	-72.0538025	-42.6874583	0.0000000
C	-64.4889109	-53.4336029	0.0000000
C	-55.3360883	-62.8640349	0.0000000
C	-44.8207076	-70.7465457	0.0000000
C	-33.2016923	-76.8870417	0.0000000
C	-20.7651411	-81.1343236	0.0000000
C	-7.8172832	-83.3838092	0.0000000

C	5.3230621	-83.5801087	0.0000000
C	18.3323359	-81.7183885	0.0000000
C	30.8902068	-77.8444904	0.0000000
C	42.6874583	-72.0538025	0.0000000
C	53.4336029	-64.4889109	0.0000000
C	62.8640349	-55.3360883	0.0000000
C	70.7465457	-44.8207076	0.0000000
C	76.8870417	-33.2016923	0.0000000
C	81.1343236	-20.7651411	0.0000000
N	84.9506790	5.2118412	0.0000000
N	83.0894836	18.4368888	0.0000000
N	79.1823493	31.2079590	0.0000000
N	73.3254828	43.2105857	0.0000000
N	65.6630996	54.1492244	0.0000000
N	56.3838729	63.7545294	0.0000000
N	45.7162882	71.7899864	0.0000000
N	33.9230167	78.0577356	0.0000000
N	21.2944480	82.4034443	0.0000000
N	8.1415393	84.7201068	0.0000000
N	-5.2118412	84.9506790	0.0000000
N	-18.4368888	83.0894836	0.0000000
N	-31.2079590	79.1823493	0.0000000
N	-43.2105857	73.3254828	0.0000000
N	-54.1492244	65.6630996	0.0000000
N	-63.7545294	56.3838729	0.0000000
N	-71.7899864	45.7162882	0.0000000
N	-78.0577356	33.9230167	0.0000000
N	-82.4034443	21.2944480	0.0000000
N	-84.7201068	8.1415393	0.0000000
N	-84.9506790	-5.2118412	0.0000000
N	-83.0894836	-18.4368888	0.0000000
N	-79.1823493	-31.2079590	0.0000000
N	-73.3254828	-43.2105857	0.0000000
N	-65.6630996	-54.1492244	0.0000000
N	-56.3838729	-63.7545294	0.0000000
N	-45.7162882	-71.7899864	0.0000000
N	-33.9230167	-78.0577356	0.0000000
N	-21.2944480	-82.4034443	0.0000000
N	-8.1415393	-84.7201068	0.0000000
N	5.2118412	-84.9506790	0.0000000
N	18.4368888	-83.0894836	0.0000000
N	31.2079590	-79.1823493	0.0000000
N	43.2105857	-73.3254828	0.0000000
N	54.1492244	-65.6630996	0.0000000
N	63.7545294	-56.3838729	0.0000000
N	71.7899864	-45.7162882	0.0000000
N	78.0577356	-33.9230167	0.0000000
N	82.4034443	-21.2944480	0.0000000
C	82.8644880	6.5215766	0.0000000

C	80.8240893	19.4041471	0.0000000
C	76.7935333	31.8089230	0.0000000
C	70.8720656	43.4304577	0.0000000
C	63.2054925	53.9825903	0.0000000
C	53.9825903	63.2054925	0.0000000
C	43.4304577	70.8720656	0.0000000
C	31.8089230	76.7935333	0.0000000
C	19.4041471	80.8240893	0.0000000
C	6.5215766	82.8644880	0.0000000
C	-6.5215766	82.8644880	0.0000000
C	-19.4041471	80.8240893	0.0000000
C	-31.8089230	76.7935333	0.0000000
C	-43.4304577	70.8720656	0.0000000
C	-53.9825903	63.2054925	0.0000000
C	-63.2054925	53.9825903	0.0000000
C	-70.8720656	43.4304577	0.0000000
C	-76.7935333	31.8089230	0.0000000
C	-80.8240893	19.4041471	0.0000000
C	-82.8644880	6.5215766	0.0000000
C	-82.8644880	-6.5215766	0.0000000
C	-80.8240893	-19.4041471	0.0000000
C	-76.7935333	-31.8089230	0.0000000
C	-70.8720656	-43.4304577	0.0000000
C	-63.2054925	-53.9825903	0.0000000
C	-53.9825903	-63.2054925	0.0000000
C	-43.4304577	-70.8720656	0.0000000
C	-31.8089230	-76.7935333	0.0000000
C	-19.4041471	-80.8240893	0.0000000
C	-6.5215766	-82.8644880	0.0000000
C	6.5215766	-82.8644880	0.0000000
C	19.4041471	-80.8240893	0.0000000
C	31.8089230	-76.7935333	0.0000000
C	43.4304577	-70.8720656	0.0000000
C	53.9825903	-63.2054925	0.0000000
C	63.2054925	-53.9825903	0.0000000
C	70.8720656	-43.4304577	0.0000000
C	76.7935333	-31.8089230	0.0000000
C	80.8240893	-19.4041471	0.0000000
H	81.7755513	6.4358755	0.0000000
H	79.7619658	19.1491538	0.0000000
H	75.7843760	31.3909164	0.0000000
H	69.9407234	42.8597304	0.0000000
H	62.3748980	53.2731956	0.0000000
H	53.2731956	62.3748980	0.0000000
H	42.8597304	69.9407234	0.0000000
H	31.3909164	75.7843760	0.0000000
H	19.1491538	79.7619658	0.0000000
H	6.4358755	81.7755513	0.0000000
H	-6.4358755	81.7755513	0.0000000

H	-19.1491538	79.7619658	0.0000000
H	-31.3909164	75.7843760	0.0000000
H	-42.8597304	69.9407234	0.0000000
H	-53.2731956	62.3748980	0.0000000
H	-62.3748980	53.2731956	0.0000000
H	-69.9407234	42.8597304	0.0000000
H	-75.7843760	31.3909164	0.0000000
H	-79.7619658	19.1491538	0.0000000
H	-81.7755513	6.4358755	0.0000000
H	-81.7755513	-6.4358755	0.0000000
H	-79.7619658	-19.1491538	0.0000000
H	-75.7843760	-31.3909164	0.0000000
H	-69.9407234	-42.8597304	0.0000000
H	-62.3748980	-53.2731956	0.0000000
H	-53.2731956	-62.3748980	0.0000000
H	-42.8597304	-69.9407234	0.0000000
H	-31.3909164	-75.7843760	0.0000000
H	-19.1491538	-79.7619658	0.0000000
H	-6.4358755	-81.7755513	0.0000000
H	6.4358755	-81.7755513	0.0000000
H	19.1491538	-79.7619658	0.0000000
H	31.3909164	-75.7843760	0.0000000
H	42.8597304	-69.9407234	0.0000000
H	53.2731956	-62.3748980	0.0000000
H	62.3748980	-53.2731956	0.0000000
H	69.9407234	-42.8597304	0.0000000
H	75.7843760	-31.3909164	0.0000000
H	79.7619658	-19.1491538	0.0000000
C	83.3838092	7.8172832	0.0000000
C	81.1343236	20.7651411	0.0000000
C	76.8870417	33.2016923	0.0000000
C	70.7465457	44.8207076	0.0000000
C	62.8640349	55.3360883	0.0000000
C	53.4336029	64.4889109	0.0000000
C	42.6874583	72.0538025	0.0000000
C	30.8902068	77.8444904	0.0000000
C	18.3323359	81.7183885	0.0000000
C	5.3230621	83.5801087	0.0000000
C	-7.8172832	83.3838092	0.0000000
C	-20.7651411	81.1343236	0.0000000
C	-33.2016923	76.8870417	0.0000000
C	-44.8207076	70.7465457	0.0000000
C	-55.3360883	62.8640349	0.0000000
C	-64.4889109	53.4336029	0.0000000
C	-72.0538025	42.6874583	0.0000000
C	-77.8444904	30.8902068	0.0000000
C	-81.7183885	18.3323359	0.0000000
C	-83.5801087	5.3230621	0.0000000
C	-83.3838092	-7.8172832	0.0000000

C	-81.1343236	-20.7651411	0.0000000
C	-76.8870417	-33.2016923	0.0000000
C	-70.7465457	-44.8207076	0.0000000
C	-62.8640349	-55.3360883	0.0000000
C	-53.4336029	-64.4889109	0.0000000
C	-42.6874583	-72.0538025	0.0000000
C	-30.8902068	-77.8444904	0.0000000
C	-18.3323359	-81.7183885	0.0000000
C	-5.3230621	-83.5801087	0.0000000
C	7.8172832	-83.3838092	0.0000000
C	20.7651411	-81.1343236	0.0000000
C	33.2016923	-76.8870417	0.0000000
C	44.8207076	-70.7465457	0.0000000
C	55.3360883	-62.8640349	0.0000000
C	64.4889109	-53.4336029	0.0000000
C	72.0538025	-42.6874583	0.0000000
C	77.8444904	-30.8902068	0.0000000
C	81.7183885	-18.3323359	0.0000000
C	82.5907128	9.0256832	0.0000000
C	80.1619562	21.8345961	0.0000000
C	75.7593461	34.1058687	0.0000000
C	69.4912895	45.5373417	0.0000000
C	61.5121268	55.8475341	0.0000000
C	52.0183313	64.7825749	0.0000000
C	41.2436719	72.1224538	0.0000000
C	29.4534563	77.6864384	0.0000000
C	16.9379990	81.3375251	0.0000000
C	4.0054719	82.9858120	0.0000000
C	-9.0256832	82.5907128	0.0000000
C	-21.8345961	80.1619562	0.0000000
C	-34.1058687	75.7593461	0.0000000
C	-45.5373417	69.4912895	0.0000000
C	-55.8475341	61.5121268	0.0000000
C	-64.7825749	52.0183313	0.0000000
C	-72.1224538	41.2436719	0.0000000
C	-77.6864384	29.4534563	0.0000000
C	-81.3375251	16.9379990	0.0000000
C	-82.9858120	4.0054719	0.0000000
C	-82.5907128	-9.0256832	0.0000000
C	-80.1619562	-21.8345961	0.0000000
C	-75.7593461	-34.1058687	0.0000000
C	-69.4912895	-45.5373417	0.0000000
C	-61.5121268	-55.8475341	0.0000000
C	-52.0183313	-64.7825749	0.0000000
C	-41.2436719	-72.1224538	0.0000000
C	-29.4534563	-77.6864384	0.0000000
C	-16.9379990	-81.3375251	0.0000000
C	-4.0054719	-82.9858120	0.0000000
C	9.0256832	-82.5907128	0.0000000

C	21.8345961	-80.1619562	0.0000000
C	34.1058687	-75.7593461	0.0000000
C	45.5373417	-69.4912895	0.0000000
C	55.8475341	-61.5121268	0.0000000
C	64.7825749	-52.0183313	0.0000000
C	72.1224538	-41.2436719	0.0000000
C	77.6864384	-29.4534563	0.0000000
C	81.3375251	-16.9379990	0.0000000
C	83.4680383	10.0747609	0.0000000
C	80.8643684	23.0080018	0.0000000
C	76.2695493	35.3747094	0.0000000
C	69.7967209	46.8703741	0.0000000
C	61.6052655	57.2119347	0.0000000
C	51.8968841	66.1447476	0.0000000
C	40.9106291	73.4488573	0.0000000
C	28.9170187	78.9444124	0.0000000
C	16.2113753	82.4960940	0.0000000
C	3.1065540	84.0164480	0.0000000
C	-10.0747609	83.4680383	0.0000000
C	-23.0080018	80.8643684	0.0000000
C	-35.3747094	76.2695493	0.0000000
C	-46.8703741	69.7967209	0.0000000
C	-57.2119347	61.6052655	0.0000000
C	-66.1447476	51.8968841	0.0000000
C	-73.4488573	40.9106291	0.0000000
C	-78.9444124	28.9170187	0.0000000
C	-82.4960940	16.2113753	0.0000000
C	-84.0164480	3.1065540	0.0000000
C	-83.4680383	-10.0747609	0.0000000
C	-80.8643684	-23.0080018	0.0000000
C	-76.2695493	-35.3747094	0.0000000
C	-69.7967209	-46.8703741	0.0000000
C	-61.6052655	-57.2119347	0.0000000
C	-51.8968841	-66.1447476	0.0000000
C	-40.9106291	-73.4488573	0.0000000
C	-28.9170187	-78.9444124	0.0000000
C	-16.2113753	-82.4960940	0.0000000
C	-3.1065540	-84.0164480	0.0000000
C	10.0747609	-83.4680383	0.0000000
C	23.0080018	-80.8643684	0.0000000
C	35.3747094	-76.2695493	0.0000000
C	46.8703741	-69.7967209	0.0000000
C	57.2119347	-61.6052655	0.0000000
C	66.1447476	-51.8968841	0.0000000
C	73.4488573	-40.9106291	0.0000000
C	78.9444124	-28.9170187	0.0000000
C	82.4960940	-16.2113753	0.0000000
C	84.7988046	9.5048507	0.0000000
C	82.2679043	22.6532859	0.0000000

C	77.7112952	35.2439220	0.0000000
C	71.2411762	46.9667357	0.0000000
C	63.0168629	57.5330725	0.0000000
C	53.2408653	66.6827542	0.0000000
C	42.1539009	74.1904851	0.0000000
C	30.0289676	79.8714000	0.0000000
C	17.1646215	83.5856161	0.0000000
C	3.8776254	85.2416768	0.0000000
C	-9.5048507	84.7988046	0.0000000
C	-22.6532859	82.2679043	0.0000000
C	-35.2439220	77.7112952	0.0000000
C	-46.9667357	71.2411762	0.0000000
C	-57.5330725	63.0168629	0.0000000
C	-66.6827542	53.2408653	0.0000000
C	-74.1904851	42.1539009	0.0000000
C	-79.8714000	30.0289676	0.0000000
C	-83.5856161	17.1646215	0.0000000
C	-85.2416768	3.8776254	0.0000000
C	-84.7988046	-9.5048507	0.0000000
C	-82.2679043	-22.6532859	0.0000000
C	-77.7112952	-35.2439220	0.0000000
C	-71.2411762	-46.9667357	0.0000000
C	-63.0168629	-57.5330725	0.0000000
C	-53.2408653	-66.6827542	0.0000000
C	-42.1539009	-74.1904851	0.0000000
C	-30.0289676	-79.8714000	0.0000000
C	-17.1646215	-83.5856161	0.0000000
C	-3.8776254	-85.2416768	0.0000000
C	9.5048507	-84.7988046	0.0000000
C	22.6532859	-82.2679043	0.0000000
C	35.2439220	-77.7112952	0.0000000
C	46.9667357	-71.2411762	0.0000000
C	57.5330725	-63.0168629	0.0000000
C	66.6827542	-53.2408653	0.0000000
C	74.1904851	-42.1539009	0.0000000
C	79.8714000	-30.0289676	0.0000000
C	83.5856161	-17.1646215	0.0000000
N	84.7201068	8.1415393	0.0000000
N	82.4034443	21.2944480	0.0000000
N	78.0577356	33.9230167	0.0000000
N	71.7899864	45.7162882	0.0000000
N	63.7545294	56.3838729	0.0000000
N	54.1492244	65.6630996	0.0000000
N	43.2105857	73.3254828	0.0000000
N	31.2079590	79.1823493	0.0000000
N	18.4368888	83.0894836	0.0000000
N	5.2118412	84.9506790	0.0000000
N	-8.1415393	84.7201068	0.0000000
N	-21.2944480	82.4034443	0.0000000

N	-33.9230167	78.0577356	0.0000000
N	-45.7162882	71.7899864	0.0000000
N	-56.3838729	63.7545294	0.0000000
N	-65.6630996	54.1492244	0.0000000
N	-73.3254828	43.2105857	0.0000000
N	-79.1823493	31.2079590	0.0000000
N	-83.0894836	18.4368888	0.0000000
N	-84.9506790	5.2118412	0.0000000
N	-84.7201068	-8.1415393	0.0000000
N	-82.4034443	-21.2944480	0.0000000
N	-78.0577356	-33.9230167	0.0000000
N	-71.7899864	-45.7162882	0.0000000
N	-63.7545294	-56.3838729	0.0000000
N	-54.1492244	-65.6630996	0.0000000
N	-43.2105857	-73.3254828	0.0000000
N	-31.2079590	-79.1823493	0.0000000
N	-18.4368888	-83.0894836	0.0000000
N	-5.2118412	-84.9506790	0.0000000
N	8.1415393	-84.7201068	0.0000000
N	21.2944480	-82.4034443	0.0000000
N	33.9230167	-78.0577356	0.0000000
N	45.7162882	-71.7899864	0.0000000
N	56.3838729	-63.7545294	0.0000000
N	65.6630996	-54.1492244	0.0000000
N	73.3254828	-43.2105857	0.0000000
N	79.1823493	-31.2079590	0.0000000
N	83.0894836	-18.4368888	0.0000000
H	90.6332634	9.7797721	0.0000000
H	87.9875241	23.8375330	0.0000000
H	83.1752399	37.3083346	0.0000000
H	76.3149054	49.8604813	0.0000000
H	67.5754445	61.1848974	0.0000000
H	57.1720520	71.0027383	0.0000000
H	45.3608937	79.0722561	0.0000000
H	32.4327998	85.1947526	0.0000000
H	18.7061026	89.2194715	0.0000000
H	4.5187992	91.0473109	0.0000000
H	-9.7797721	90.6332634	0.0000000
H	-23.8375330	87.9875241	0.0000000
H	-37.3083346	83.1752399	0.0000000
H	-49.8604813	76.3149054	0.0000000
H	-61.1848974	67.5754445	0.0000000
H	-71.0027383	57.1720520	0.0000000
H	-79.0722561	45.3608937	0.0000000
H	-85.1947526	32.4327998	0.0000000
H	-89.2194715	18.7061026	0.0000000
H	-91.0473109	4.5187992	0.0000000
H	-90.6332634	-9.7797721	0.0000000
H	-87.9875241	-23.8375330	0.0000000

H	-83.1752399	-37.3083346	0.0000000
H	-76.3149054	-49.8604813	0.0000000
H	-67.5754445	-61.1848974	0.0000000
H	-57.1720520	-71.0027383	0.0000000
H	-45.3608937	-79.0722561	0.0000000
H	-32.4327998	-85.1947526	0.0000000
H	-18.7061026	-89.2194715	0.0000000
H	-4.5187992	-91.0473109	0.0000000
H	9.7797721	-90.6332634	0.0000000
H	23.8375330	-87.9875241	0.0000000
H	37.3083346	-83.1752399	0.0000000
H	49.8604813	-76.3149054	0.0000000
H	61.1848974	-67.5754445	0.0000000
H	71.0027383	-57.1720520	0.0000000
H	79.0722561	-45.3608937	0.0000000
H	85.1947526	-32.4327998	0.0000000
H	89.2194715	-18.7061026	0.0000000
H	88.5825859	11.5611431	0.0000000
H	85.6834261	25.2761756	0.0000000
H	80.6744559	38.3688249	0.0000000
H	73.6790129	50.5167063	0.0000000
H	64.8693480	61.4206988	0.0000000
H	54.4623845	70.8123099	0.0000000
H	42.7143764	78.4602868	0.0000000
H	29.9145985	84.1763111	0.0000000
H	16.3782240	87.8196352	0.0000000
H	2.4385632	89.3005485	0.0000000
H	-11.5611431	88.5825859	0.0000000
H	-25.2761756	85.6834261	0.0000000
H	-38.3688249	80.6744559	0.0000000
H	-50.5167063	73.6790129	0.0000000
H	-61.4206988	64.8693480	0.0000000
H	-70.8123099	54.4623845	0.0000000
H	-78.4602868	42.7143764	0.0000000
H	-84.1763111	29.9145985	0.0000000
H	-87.8196352	16.3782240	0.0000000
H	-89.3005485	2.4385632	0.0000000
H	-88.5825859	-11.5611431	0.0000000
H	-85.6834261	-25.2761756	0.0000000
H	-80.6744559	-38.3688249	0.0000000
H	-73.6790129	-50.5167063	0.0000000
H	-64.8693480	-61.4206988	0.0000000
H	-54.4623845	-70.8123099	0.0000000
H	-42.7143764	-78.4602868	0.0000000
H	-29.9145985	-84.1763111	0.0000000
H	-16.3782240	-87.8196352	0.0000000
H	-2.4385632	-89.3005485	0.0000000
H	11.5611431	-88.5825859	0.0000000
H	25.2761756	-85.6834261	0.0000000

H	38.3688249	-80.6744559	0.0000000
H	50.5167063	-73.6790129	0.0000000
H	61.4206988	-64.8693480	0.0000000
H	70.8123099	-54.4623845	0.0000000
H	78.4602868	-42.7143764	0.0000000
H	84.1763111	-29.9145985	0.0000000
H	87.8196352	-16.3782240	0.0000000
H	91.0473109	4.5187992	0.0000000
H	89.2194715	18.7061026	0.0000000
H	85.1947526	32.4327998	0.0000000
H	79.0722561	45.3608937	0.0000000
H	71.0027383	57.1720520	0.0000000
H	61.1848974	67.5754445	0.0000000
H	49.8604813	76.3149054	0.0000000
H	37.3083346	83.1752399	0.0000000
H	23.8375330	87.9875241	0.0000000
H	9.7797721	90.6332634	0.0000000
H	-4.5187992	91.0473109	0.0000000
H	-18.7061026	89.2194715	0.0000000
H	-32.4327998	85.1947526	0.0000000
H	-45.3608937	79.0722561	0.0000000
H	-57.1720520	71.0027383	0.0000000
H	-67.5754445	61.1848974	0.0000000
H	-76.3149054	49.8604813	0.0000000
H	-83.1752399	37.3083346	0.0000000
H	-87.9875241	23.8375330	0.0000000
H	-90.6332634	9.7797721	0.0000000
H	-91.0473109	-4.5187992	0.0000000
H	-89.2194715	-18.7061026	0.0000000
H	-85.1947526	-32.4327998	0.0000000
H	-79.0722561	-45.3608937	0.0000000
H	-71.0027383	-57.1720520	0.0000000
H	-61.1848974	-67.5754445	0.0000000
H	-49.8604813	-76.3149054	0.0000000
H	-37.3083346	-83.1752399	0.0000000
H	-23.8375330	-87.9875241	0.0000000
H	-9.7797721	-90.6332634	0.0000000
H	4.5187992	-91.0473109	0.0000000
H	18.7061026	-89.2194715	0.0000000
H	32.4327998	-85.1947526	0.0000000
H	45.3608937	-79.0722561	0.0000000
H	57.1720520	-71.0027383	0.0000000
H	67.5754445	-61.1848974	0.0000000
H	76.3149054	-49.8604813	0.0000000
H	83.1752399	-37.3083346	0.0000000
H	87.9875241	-23.8375330	0.0000000
H	89.3005485	2.4385632	0.0000000
H	87.8196352	16.3782240	0.0000000
H	84.1763111	29.9145985	0.0000000

H	78.4602868	42.7143764	0.0000000
H	70.8123099	54.4623845	0.0000000
H	61.4206988	64.8693480	0.0000000
H	50.5167063	73.6790129	0.0000000
H	38.3688249	80.6744559	0.0000000
H	25.2761756	85.6834261	0.0000000
H	11.5611431	88.5825859	0.0000000
H	-2.4385632	89.3005485	0.0000000
H	-16.3782240	87.8196352	0.0000000
H	-29.9145985	84.1763111	0.0000000
H	-42.7143764	78.4602868	0.0000000
H	-54.4623845	70.8123099	0.0000000
H	-64.8693480	61.4206988	0.0000000
H	-73.6790129	50.5167063	0.0000000
H	-80.6744559	38.3688249	0.0000000
H	-85.6834261	25.2761756	0.0000000
H	-88.5825859	11.5611431	0.0000000
H	-89.3005485	-2.4385632	0.0000000
H	-87.8196352	-16.3782240	0.0000000
H	-84.1763111	-29.9145985	0.0000000
H	-78.4602868	-42.7143764	0.0000000
H	-70.8123099	-54.4623845	0.0000000
H	-61.4206988	-64.8693480	0.0000000
H	-50.5167063	-73.6790129	0.0000000
H	-38.3688249	-80.6744559	0.0000000
H	-25.2761756	-85.6834261	0.0000000
H	-11.5611431	-88.5825859	0.0000000
H	2.4385632	-89.3005485	0.0000000
H	16.3782240	-87.8196352	0.0000000
H	29.9145985	-84.1763111	0.0000000
H	42.7143764	-78.4602868	0.0000000
H	54.4623845	-70.8123099	0.0000000
H	64.8693480	-61.4206988	0.0000000
H	73.6790129	-50.5167063	0.0000000
H	80.6744559	-38.3688249	0.0000000
H	85.6834261	-25.2761756	0.0000000
H	83.9652087	2.0205721	0.0000000
H	82.6153705	15.1307480	0.0000000
H	79.2312677	27.8683547	0.0000000
H	73.8962282	39.9197500	0.0000000
H	66.7416182	50.9881886	0.0000000
H	57.9436082	60.8011287	0.0000000
H	47.7188342	69.1169433	0.0000000
H	36.3190641	75.7308693	0.0000000
H	24.0249981	80.4800500	0.0000000
H	11.1393570	83.2475447	0.0000000
H	-2.0205721	83.9652087	0.0000000
H	-15.1307480	82.6153705	0.0000000
H	-27.8683547	79.2312677	0.0000000

H	-39.9197500	73.8962282	0.0000000
H	-50.9881886	66.7416182	0.0000000
H	-60.8011287	57.9436082	0.0000000
H	-69.1169433	47.7188342	0.0000000
H	-75.7308693	36.3190641	0.0000000
H	-80.4800500	24.0249981	0.0000000
H	-83.2475447	11.1393570	0.0000000
H	-83.9652087	-2.0205721	0.0000000
H	-82.6153705	-15.1307480	0.0000000
H	-79.2312677	-27.8683547	0.0000000
H	-73.8962282	-39.9197500	0.0000000
H	-66.7416182	-50.9881886	0.0000000
H	-57.9436082	-60.8011287	0.0000000
H	-47.7188342	-69.1169433	0.0000000
H	-36.3190641	-75.7308693	0.0000000
H	-24.0249981	-80.4800500	0.0000000
H	-11.1393570	-83.2475447	0.0000000
H	2.0205721	-83.9652087	0.0000000
H	15.1307480	-82.6153705	0.0000000
H	27.8683547	-79.2312677	0.0000000
H	39.9197500	-73.8962282	0.0000000
H	50.9881886	-66.7416182	0.0000000
H	60.8011287	-57.9436082	0.0000000
H	69.1169433	-47.7188342	0.0000000
H	75.7308693	-36.3190641	0.0000000
H	80.4800500	-24.0249981	0.0000000
H	81.9159855	3.8016111	0.0000000
H	80.3127608	16.5692904	0.0000000
H	76.7319694	28.9289787	0.0000000
H	71.2617822	40.5763395	0.0000000
H	64.0368934	51.2245762	0.0000000
H	55.2352038	60.6114939	0.0000000
H	45.0734402	68.5059553	0.0000000
H	33.8018189	74.7135729	0.0000000
H	21.6978846	79.0814943	0.0000000
H	9.0596764	81.5021668	0.0000000
H	-3.8016111	81.9159855	0.0000000
H	-16.5692904	80.3127608	0.0000000
H	-28.9289787	76.7319694	0.0000000
H	-40.5763395	71.2617822	0.0000000
H	-51.2245762	64.0368934	0.0000000
H	-60.6114939	55.2352038	0.0000000
H	-68.5059553	45.0734402	0.0000000
H	-74.7135729	33.8018189	0.0000000
H	-79.0814943	21.6978846	0.0000000
H	-81.5021668	9.0596764	0.0000000
H	-81.9159855	-3.8016111	0.0000000
H	-80.3127608	-16.5692904	0.0000000
H	-76.7319694	-28.9289787	0.0000000

H	-71.2617822	-40.5763395	0.0000000
H	-64.0368934	-51.2245762	0.0000000
H	-55.2352038	-60.6114939	0.0000000
H	-45.0734402	-68.5059553	0.0000000
H	-33.8018189	-74.7135729	0.0000000
H	-21.6978846	-79.0814943	0.0000000
H	-9.0596764	-81.5021668	0.0000000
H	3.8016111	-81.9159855	0.0000000
H	16.5692904	-80.3127608	0.0000000
H	28.9289787	-76.7319694	0.0000000
H	40.5763395	-71.2617822	0.0000000
H	51.2245762	-64.0368934	0.0000000
H	60.6114939	-55.2352038	0.0000000
H	68.5059553	-45.0734402	0.0000000
H	74.7135729	-33.8018189	0.0000000
H	79.0814943	-21.6978846	0.0000000
H	81.5021668	9.0596764	0.0000000
H	79.0814943	21.6978846	0.0000000
H	74.7135729	33.8018189	0.0000000
H	68.5059553	45.0734402	0.0000000
H	60.6114939	55.2352038	0.0000000
H	51.2245762	64.0368934	0.0000000
H	40.5763395	71.2617822	0.0000000
H	28.9289787	76.7319694	0.0000000
H	16.5692904	80.3127608	0.0000000
H	3.8016111	81.9159855	0.0000000
H	-9.0596764	81.5021668	0.0000000
H	-21.6978846	79.0814943	0.0000000
H	-33.8018189	74.7135729	0.0000000
H	-45.0734402	68.5059553	0.0000000
H	-55.2352038	60.6114939	0.0000000
H	-64.0368934	51.2245762	0.0000000
H	-71.2617822	40.5763395	0.0000000
H	-76.7319694	28.9289787	0.0000000
H	-80.3127608	16.5692904	0.0000000
H	-81.9159855	3.8016111	0.0000000
H	-81.5021668	-9.0596764	0.0000000
H	-79.0814943	-21.6978846	0.0000000
H	-74.7135729	-33.8018189	0.0000000
H	-68.5059553	-45.0734402	0.0000000
H	-60.6114939	-55.2352038	0.0000000
H	-51.2245762	-64.0368934	0.0000000
H	-40.5763395	-71.2617822	0.0000000
H	-28.9289787	-76.7319694	0.0000000
H	-16.5692904	-80.3127608	0.0000000
H	-3.8016111	-81.9159855	0.0000000
H	9.0596764	-81.5021668	0.0000000
H	21.6978846	-79.0814943	0.0000000
H	33.8018189	-74.7135729	0.0000000

H	45.0734402	-68.5059553	0.0000000
H	55.2352038	-60.6114939	0.0000000
H	64.0368934	-51.2245762	0.0000000
H	71.2617822	-40.5763395	0.0000000
H	76.7319694	-28.9289787	0.0000000
H	80.3127608	-16.5692904	0.0000000
H	83.2475447	11.1393570	0.0000000
H	80.4800500	24.0249981	0.0000000
H	75.7308693	36.3190641	0.0000000
H	69.1169433	47.7188342	0.0000000
H	60.8011287	57.9436082	0.0000000
H	50.9881886	66.7416182	0.0000000
H	39.9197500	73.8962282	0.0000000
H	27.8683547	79.2312677	0.0000000
H	15.1307480	82.6153705	0.0000000
H	2.0205721	83.9652087	0.0000000
H	-11.1393570	83.2475447	0.0000000
H	-24.0249981	80.4800500	0.0000000
H	-36.3190641	75.7308693	0.0000000
H	-47.7188342	69.1169433	0.0000000
H	-57.9436082	60.8011287	0.0000000
H	-66.7416182	50.9881886	0.0000000
H	-73.8962282	39.9197500	0.0000000
H	-79.2312677	27.8683547	0.0000000
H	-82.6153705	15.1307480	0.0000000
H	-83.9652087	2.0205721	0.0000000
H	-83.2475447	-11.1393570	0.0000000
H	-80.4800500	-24.0249981	0.0000000
H	-75.7308693	-36.3190641	0.0000000
H	-69.1169433	-47.7188342	0.0000000
H	-60.8011287	-57.9436082	0.0000000
H	-50.9881886	-66.7416182	0.0000000
H	-39.9197500	-73.8962282	0.0000000
H	-27.8683547	-79.2312677	0.0000000
H	-15.1307480	-82.6153705	0.0000000
H	-2.0205721	-83.9652087	0.0000000
H	11.1393570	-83.2475447	0.0000000
H	24.0249981	-80.4800500	0.0000000
H	36.3190641	-75.7308693	0.0000000
H	47.7188342	-69.1169433	0.0000000
H	57.9436082	-60.8011287	0.0000000
H	66.7416182	-50.9881886	0.0000000
H	73.8962282	-39.9197500	0.0000000
H	79.2312677	-27.8683547	0.0000000
H	82.6153705	-15.1307480	0.0000000
C	86.0021351	10.2613131	0.0000000
C	83.3380831	23.5886773	0.0000000
C	78.6219709	36.3352100	0.0000000
C	71.9699248	48.1870492	0.0000000

C	63.5457404	58.8523634	0.0000000
C	53.5568489	68.0685370	0.0000000
C	42.2492100	75.6086374	0.0000000
C	29.9012554	81.2870022	0.0000000
C	16.8170326	84.9638112	0.0000000
C	3.3187187	86.5485292	0.0000000
C	-10.2613131	86.0021351	0.0000000
C	-23.5886773	83.3380831	0.0000000
C	-36.3352100	78.6219709	0.0000000
C	-48.1870492	71.9699248	0.0000000
C	-58.8523634	63.5457404	0.0000000
C	-68.0685370	53.5568489	0.0000000
C	-75.6086374	42.2492100	0.0000000
C	-81.2870022	29.9012554	0.0000000
C	-84.9638112	16.8170326	0.0000000
C	-86.5485292	3.3187187	0.0000000
C	-86.0021351	-10.2613131	0.0000000
C	-83.3380831	-23.5886773	0.0000000
C	-78.6219709	-36.3352100	0.0000000
C	-71.9699248	-48.1870492	0.0000000
C	-63.5457404	-58.8523634	0.0000000
C	-53.5568489	-68.0685370	0.0000000
C	-42.2492100	-75.6086374	0.0000000
C	-29.9012554	-81.2870022	0.0000000
C	-16.8170326	-84.9638112	0.0000000
C	-3.3187187	-86.5485292	0.0000000
C	10.2613131	-86.0021351	0.0000000
C	23.5886773	-83.3380831	0.0000000
C	36.3352100	-78.6219709	0.0000000
C	48.1870492	-71.9699248	0.0000000
C	58.8523634	-63.5457404	0.0000000
C	68.0685370	-53.5568489	0.0000000
C	75.6086374	-42.2492100	0.0000000
C	81.2870022	-29.9012554	0.0000000
C	84.9638112	-16.8170326	0.0000000
C	85.8794131	11.6714430	0.0000000
C	82.9962791	24.9622482	0.0000000
C	78.0695013	37.6384000	0.0000000
C	71.2203932	49.3877695	0.0000000
C	62.6176027	59.9210482	0.0000000
C	52.4729589	68.9788719	0.0000000
C	41.0362568	76.3382067	0.0000000
C	28.5891059	81.8178416	0.0000000
C	15.4379962	85.2828497	0.0000000
C	1.9067519	86.6479110	0.0000000
C	-11.6714430	85.8794131	0.0000000
C	-24.9622482	82.9962791	0.0000000
C	-37.6384000	78.0695013	0.0000000
C	-49.3877695	71.2203932	0.0000000

C	-59.9210482	62.6176027	0.0000000
C	-68.9788719	52.4729589	0.0000000
C	-76.3382067	41.0362568	0.0000000
C	-81.8178416	28.5891059	0.0000000
C	-85.2828497	15.4379962	0.0000000
C	-86.6479110	1.9067519	0.0000000
C	-85.8794131	-11.6714430	0.0000000
C	-82.9962791	-24.9622482	0.0000000
C	-78.0695013	-37.6384000	0.0000000
C	-71.2203932	-49.3877695	0.0000000
C	-62.6176027	-59.9210482	0.0000000
C	-52.4729589	-68.9788719	0.0000000
C	-41.0362568	-76.3382067	0.0000000
C	-28.5891059	-81.8178416	0.0000000
C	-15.4379962	-85.2828497	0.0000000
C	-1.9067519	-86.6479110	0.0000000
C	11.6714430	-85.8794131	0.0000000
C	24.9622482	-82.9962791	0.0000000
C	37.6384000	-78.0695013	0.0000000
C	49.3877695	-71.2203932	0.0000000
C	59.9210482	-62.6176027	0.0000000
C	68.9788719	-52.4729589	0.0000000
C	76.3382067	-41.0362568	0.0000000
C	81.8178416	-28.5891059	0.0000000
C	85.2828497	-15.4379962	0.0000000
C	85.7267084	12.8903549	0.0000000
C	82.6547746	26.1422650	0.0000000
C	77.5476059	38.7504658	0.0000000
C	70.5309578	50.4045015	0.0000000
C	61.7776035	60.8174111	0.0000000
C	51.5030795	69.7327942	0.0000000
C	39.9603787	76.9311245	0.0000000
C	27.4337209	82.2351552	0.0000000
C	14.2315537	85.5142834	0.0000000
C	0.6789585	86.6877662	0.0000000
C	-12.8903549	85.7267084	0.0000000
C	-26.1422650	82.6547746	0.0000000
C	-38.7504658	77.5476059	0.0000000
C	-50.4045015	70.5309578	0.0000000
C	-60.8174111	61.7776035	0.0000000
C	-69.7327942	51.5030795	0.0000000
C	-76.9311245	39.9603787	0.0000000
C	-82.2351552	27.4337209	0.0000000
C	-85.5142834	14.2315537	0.0000000
C	-86.6877662	0.6789585	0.0000000
C	-85.7267084	-12.8903549	0.0000000
C	-82.6547746	-26.1422650	0.0000000
C	-77.5476059	-38.7504658	0.0000000
C	-70.5309578	-50.4045015	0.0000000

C	-61.7776035	-60.8174111	0.0000000
C	-51.5030795	-69.7327942	0.0000000
C	-39.9603787	-76.9311245	0.0000000
C	-27.4337209	-82.2351552	0.0000000
C	-14.2315537	-85.5142834	0.0000000
C	-0.6789585	-86.6877662	0.0000000
C	12.8903549	-85.7267084	0.0000000
C	26.1422650	-82.6547746	0.0000000
C	38.7504658	-77.5476059	0.0000000
C	50.4045015	-70.5309578	0.0000000
C	60.8174111	-61.7776035	0.0000000
C	69.7327942	-51.5030795	0.0000000
C	76.9311245	-39.9603787	0.0000000
C	82.2351552	-27.4337209	0.0000000
C	85.5142834	-14.2315537	0.0000000
C	85.5142834	14.2315537	0.0000000
C	82.2351552	27.4337209	0.0000000
C	76.9311245	39.9603787	0.0000000
C	69.7327942	51.5030795	0.0000000
C	60.8174111	61.7776035	0.0000000
C	50.4045015	70.5309578	0.0000000
C	38.7504658	77.5476059	0.0000000
C	26.1422650	82.6547746	0.0000000
C	12.8903549	85.7267084	0.0000000
C	-0.6789585	86.6877662	0.0000000
C	-14.2315537	85.5142834	0.0000000
C	-27.4337209	82.2351552	0.0000000
C	-39.9603787	76.9311245	0.0000000
C	-51.5030795	69.7327942	0.0000000
C	-61.7776035	60.8174111	0.0000000
C	-70.5309578	50.4045015	0.0000000
C	-77.5476059	38.7504658	0.0000000
C	-82.6547746	26.1422650	0.0000000
C	-85.7267084	12.8903549	0.0000000
C	-86.6877662	-0.6789585	0.0000000
C	-85.5142834	-14.2315537	0.0000000
C	-82.2351552	-27.4337209	0.0000000
C	-76.9311245	-39.9603787	0.0000000
C	-69.7327942	-51.5030795	0.0000000
C	-60.8174111	-61.7776035	0.0000000
C	-50.4045015	-70.5309578	0.0000000
C	-38.7504658	-77.5476059	0.0000000
C	-26.1422650	-82.6547746	0.0000000
C	-12.8903549	-85.7267084	0.0000000
C	0.6789585	-86.6877662	0.0000000
C	14.2315537	-85.5142834	0.0000000
C	27.4337209	-82.2351552	0.0000000
C	39.9603787	-76.9311245	0.0000000
C	51.5030795	-69.7327942	0.0000000

C	61.7776035	-60.8174111	0.0000000
C	70.5309578	-50.4045015	0.0000000
C	77.5476059	-38.7504658	0.0000000
C	82.6547746	-26.1422650	0.0000000
C	85.7267084	-12.8903549	0.0000000
C	85.2828497	15.4379962	0.0000000
C	81.8178416	28.5891059	0.0000000
C	76.3382067	41.0362568	0.0000000
C	68.9788719	52.4729589	0.0000000
C	59.9210482	62.6176027	0.0000000
C	49.3877695	71.2203932	0.0000000
C	37.6384000	78.0695013	0.0000000
C	24.9622482	82.9962791	0.0000000
C	11.6714430	85.8794131	0.0000000
C	-1.9067519	86.6479110	0.0000000
C	-15.4379962	85.2828497	0.0000000
C	-28.5891059	81.8178416	0.0000000
C	-41.0362568	76.3382067	0.0000000
C	-52.4729589	68.9788719	0.0000000
C	-62.6176027	59.9210482	0.0000000
C	-71.2203932	49.3877695	0.0000000
C	-78.0695013	37.6384000	0.0000000
C	-82.9962791	24.9622482	0.0000000
C	-85.8794131	11.6714430	0.0000000
C	-86.6479110	-1.9067519	0.0000000
C	-85.2828497	-15.4379962	0.0000000
C	-81.8178416	-28.5891059	0.0000000
C	-76.3382067	-41.0362568	0.0000000
C	-68.9788719	-52.4729589	0.0000000
C	-59.9210482	-62.6176027	0.0000000
C	-49.3877695	-71.2203932	0.0000000
C	-37.6384000	-78.0695013	0.0000000
C	-24.9622482	-82.9962791	0.0000000
C	-11.6714430	-85.8794131	0.0000000
C	1.9067519	-86.6479110	0.0000000
C	15.4379962	-85.2828497	0.0000000
C	28.5891059	-81.8178416	0.0000000
C	41.0362568	-76.3382067	0.0000000
C	52.4729589	-68.9788719	0.0000000
C	62.6176027	-59.9210482	0.0000000
C	71.2203932	-49.3877695	0.0000000
C	78.0695013	-37.6384000	0.0000000
C	82.9962791	-24.9622482	0.0000000
C	85.8794131	-11.6714430	0.0000000
